# Microevolution of the noble crayfish (*Astacus astacus*) in the Southern Balkan Peninsula

**DOI:** 10.1186/s12862-017-0971-6

**Published:** 2017-05-30

**Authors:** Anastasia Laggis, Athanasios D. Baxevanis, Alexandra Charalampidou, Stefania Maniatsi, Alexander Triantafyllidis, Theodore J. Abatzopoulos

**Affiliations:** 1 Department of Genetics, Development and Molecular Biology, School of Biology, Aristotle University of Thessaloniki, 54124 Thessaloniki, Macedonia Greece; 2 Scientific Computing Office, Information Technology (IT) Center, School of Sciences, Aristotle University of Thessaloniki, 541 24 Thessaloniki, Macedonia Greece

**Keywords:** Noble crayfish, mtDNA, 16S, COI, Microsatellites, Europe, Balkans, Phylogeny, Populations

## Abstract

**Background:**

The noble crayfish (*Astacus astacus*) displays a complex historical and contemporary genetic status in Europe. The species divergence has been shaped by geological events (i.e. Pleistocene glaciations) and humanly induced impacts (i.e. translocations, pollution, etc.) on its populations due to species commercial value and its niche degradation. Until now, limited genetic information has been procured for the Balkan area and especially for the southernmost distribution of this species (i.e. Greece). It is well known that the rich habitat diversity of the Balkan Peninsula offers suitable conditions for genetically diversified populations. Thus, the present manuscript revisits the phylogenetic relationships of the noble crayfish in Europe and identifies the genetic make-up and the biogeographical patterns of the species in its southern range limit.

**Results:**

Mitochondrial markers (i.e. COI and 16S) were used in order to elucidate the genetic structure and diversity of the noble crayfish in Europe. Two of the six European haplotypic lineages, were found exclusively in Greece. These two lineages exhibited greater haplotypic richness when compared with the rest four (of “Central European” origin) while they showed high genetic diversity. Divergence time analysis identified that the majority of this divergence was captured through Pleistocene, suggesting a southern glacial refugium (Greece, southern Balkans). Furthermore, six microsatellite markers were used in order to define the factors affecting the genetic structure and demographic history of the species in Greece. The population structure analysis revealed six to nine genetic clusters and eight putative genetic barriers. Evidence of bottleneck effects in the last ~5000 years (due to climatic and geological events and human activities) is also afforded. Findings from several other research fields (e.g. life sciences, geology or even archaeology) have been utilized to perceive the genetic make-up of the noble crayfish.

**Conclusions:**

The southernmost part of Balkans has played a major role as a glacial refugium for *A. astacus*. Such refugia have served as centres of expansion to northern regions. Recent history of the noble crayfish in southern Balkans reveals the influence of environmental (climate, geology and/or topology) and anthropogenic factors.

**Electronic supplementary material:**

The online version of this article (doi:10.1186/s12862-017-0971-6) contains supplementary material, which is available to authorized users.

## Background

Quaternary climatic oscillations have influenced the flora (e.g. [1–3]) and fauna (e.g. [4–6]) of Europe. Pleistocene glaciations had substantial impact on the flora and fauna directly via major biogeographic events (e.g. displacement) and habitat alterations, while indirectly, via fluctuations in environmental conditions [7–9]. The distribution and structure of obligate freshwater organisms frequently reflects historic, geological processes (such as tectonic activity, sea level change and glaciation) due to their dependency to the aquatic environment [10]. The long-term survival of many species depended on refugia and their current distribution, genetic structure and diversity reflects such historical processes [7–9, 11]. In Europe, the Balkan Peninsula is considered one of the major glacial refugia for many species [8]. It, also, played an important role in the colonization of eastern and western parts of Europe [7]. The majority of the European temperate fishes seem to have colonized the continent from the Black Sea through the Danube and Dnieper rivers [8].

More specifically, Greece with its complex geographic landscape [12], coupled with its geographic location (southernmost part of the Balkan Peninsula), offered suitable conditions for many species during glaciations (e.g. [13, 14]). In this area, the geological processes (tectonic and seismic activity) as well as climatic conditions forged a complex geographic landscape with rich habitat diversity [12]. Consequently, several restricted alluvial reaches [12] and small climatically stable areas [15] were formed, influencing the flora [1, 2] and fauna [13, 14]. During the mid- and late Holocene, the anthropogenic influence was intensified, generating the modification - to some extent - of the natural environment [16, 17].

An example of obligate freshwater species that could serve as a “model organism” for tackling historical events and biogeographical processes is the noble crayfish (*Astacus astacus*). It is a well-established European freshwater crustacean and its distribution expands from Norway (North end) to Greece (South end) and from France (West end) to Russia (East end) [18]. It plays an important role on the freshwater ecosystems, with its wide habitat usage and biological features [19]. The species is harboured in a plethora of inland freshwater habitats (streams, rivers, lakes, ponds and reservoirs [19]) and it is an opportunistic, omnivorous feeder [2, 3]. Sexual maturity is reached between 2 and 5 years of age [19, 20]. Females may be reproductively inactive for long periods (from one to several years), depending on the ambient water temperature [21]. The estimated life expectancy varies between 13 and 20 years [20], with the upper limit considered as uncommon [22], anecdotal [23] or even doubted [20]. Cukerzis (1988, cited in Keller [24]) estimated the probable longevity of the noble crayfish in Central Europe to be 7-8 years. The species has high oxygen demands [20], is sensitive to organic pollution [25] and is considered as a water quality indicator [26].

The economic and cultural value of the noble crayfish is well known in the Central and Northern Europe [19]. At the same time, several humanly induced interventions (e.g. habitat degradation, introduction of foreign species and crayfish plague) affect negatively the noble crayfish ([23] and references therein). Despite their wide distribution, the IUCN estimated a decline rate of the species between 50% and 70% [23]. Thus, the “IUCN Red List of Threatened Species” classifies the noble crayfish as a vulnerable species [23], while the “Red book of threatened fauna of Greece” classifies it at the unknown status [27]. It is worth noting that international treaties and conventions have attributed special protection to the noble crayfish (Bern Convention Appendix III, EU Habitats Directive Appendix V and directive 92/43/EEC). In order to compensate the decline or loss of populations, reintroduction of the species has been widely applied in Europe as a management tool [28]. However, variation of regulations between European countries and regional authorities based mainly on cultural traditions [19] complicates the preservation of the species.

Translocations may affect noble crayfish by influencing its range expansion, gene flow and gene pool, as identified by several studies using various molecular markers [16, 17]. It is important to notice that the majority of those translocations have disregarded the genetic structure of noble crayfish populations. The necessity of recording the genetic pool prior translocations has been recently pointed out [16, 17, 29]. To our knowledge, only one study has used microsatellite markers as monitoring tool of already translocated noble crayfish populations in the Czech Republic; the latter research was focused on the genetic variation of two translocated populations to evaluate the success of the managerial protocol applied in the last decade [30].

In the past, increasing attention was paid upon the genetic structure of the noble crayfish populations using allozymes ([31] and references therein), ISSR-PCR [32], RAPD-PCR [33] and microsatellites within rDNA-ITS1 ([34] and references therein). During the last years, three large-scale genetic analyses have been published, diagnosing the genetic diversity of the species between large geographical areas [16, 17, 29]. Microsatellite analysis differentiated Northern European (Estonia, Finland and Sweden) populations from Central European (Germany and Czech Republic) populations, with the former exhibiting lower genetic variation [17]. Furthermore, Schrimpf et al. [16, 29] revealed higher genetic diversity in the Black Sea basin populations compared to those of Southern Baltic, North and Adriatic Seas.

To our knowledge, there is currently no molecular study that encompassed samples from the southernmost limit of the species distribution (i.e. Greece). It should be noted, there are a few studies incorporating Greek crayfish to their genetic analysis, but they all refer to the stone crayfish, *Austropotamobius torrentium* [5, 35]. The few, sporadically published, studies about the noble crayfish in Greece are reporting on its geographical distribution and/or general information ([36] and references therein).

The lack of genetic information on the noble crayfish of Greece, coupled with the particular geographic features of the region, initiated the present study. This study largely focuses on *Astacus astacus* populations in the southernmost Balkan Peninsula, which has not been included in previous assessments [16, 29] although it is considered as a potentially important glacial refugium. Mitochondrial (COI and 16S) haplotypes were used to further investigate the phylogenetic relationships of the noble crayfish in Europe. Knowing that mitochondrial DNA may have little practical value in population/conservation genetic studies of widespread organisms [37], microsatellite markers were used in order to detect potential genetic differentiation and recent demographic events that may have shaped the population structure of the noble crayfish in its southernmost distribution range (i.e. Greece). The genetic make-up and the biogeographical patterns of the Greek populations are analyzed to identify factors affecting its current structure and distribution. To accomplish the above goals (i.e. phylogeography of *A. astacus* in Europe and the genetic structure of the species in its southernmost distribution range) extensive sampling in the continental Greece was performed in an exhaustive manner. The genetic structure of the species is meant to be utilized as a baseline for management and conservation activities.

## Methods

### Sample collection and DNA extraction

Two hundred eighty four potential noble crayfish sites have been investigated in continental Greece. The selection of the potential sites was inferred from the bibliography ([36] and references therein), information from local residents and habitat requirements of the species (high oxygen demand and availability of shelters [20]). In order to maximise our chances to capture crayfish in areas where sporadic observations were reported from local residents, a wide search of the river catchment was performed. For instance, in Pinios river catchment (Thessaly district) a total of 53 potential sampling sites were visited and explored. Similarly, in Kalamas river catchment (Epirus district), 7 sampling sites were searched for crayfish. The thorough examination of the continental Greece resulted in the sampling of noble crayfish in 21 sites (from a total of 284 explored sites).

Most of the noble crayfish samples were collected by hand during the day. When the conditions were not favorable, LiNi traps [38] baited with meat were used during the night. The only exception was one individual in KEF site (see Table 1), which was captured while moving <2 m from the riverbank fleeing from it. In all potential sites sampled by hand, an area between 50 and 250 m was thoroughly examined (following the upward direction of the river flow) and lasted between 30 min and 1 h 30 min (sampling was performed by at least two researchers). Sampling was repeated in sites where the number of sampled individuals was less than 20 or if the presence of noble crayfish had been reported previously. Individuals were transferred to the laboratory in an isotherm with ice packs (individuals from different sites were stored in separate plastic bags). Fishing license for research purposes was retrieved from the Greek authorities. In total, 284 noble crayfish were collected from 21 sites (Table 1) and stored at −20 °C. Genomic DNA was extracted from pereiopods muscle tissue (≈ 10 mg) following the protocol of Estoup et al. [39].

**Table 1:** Information on sampling collection, where sites, abbreviation, habitat type, geographical coordinate, collection method, year of sampling (Year), sample size, number of mitochondrial sequences (mtDNA), number of microsatellite genotyped data (nDNA) and status are given

Site	Abbreviation	Habitat type	Water Basin^a^	Geographic coordinates	Collection method	Year	Sample size	mtDNA	nDna	Status
Arahthos	ARX	River	Arahthos	N39°15′ E21°00′	Trap (recreational fisherman)	2011	1	1	-	Unknown
Kalamas	KAS	River	Kalamas	N39°39′ E20°31′ ^b^	Trap (from Perdikaris)	2004	20	2	20	Transfer from 1) Larissa (80’s)^d^ and 2) unknown origins (years 1990 and 1991)^e^
Tzaravina	TZA	Lake	Kalamas	N39°54′ E20°30′	Hand	2011	6	2	6	Transfer from 1) Larissa (80’s)^d^ and 2) unknown origins (years 1990 and 1991)^e^
Chani Kaber Aga	KPA	Stream	Arahthos	N39°43′ E20°57′	Hand	2011	6	2	6	Unknown
Aoo1	AA1	Lake	Aoos	N39°49′ E21°07′ ^b^	Trap (fisherman)	2009	20	2	20	Unknown
Aoo2	AA2	Lake	Aoos	N39°48′ E21°06′ ^b^	Trap (fisherman)	2011	20	2	20	Unknown
Fragkades	FRA	Stream	Aoos	N39°50′ E20°48′	Hand	2011	2	2	-	Unknown
Kalivia	KLV	Stream	Acheloos	N39°18′ E21°42′	Hand	2011, 2012	16	2	16	Unknown
Neochori	NEO	Stream	Acheloos	N39°17′ E21°43′	Hand	2011, 2012	11	2	11	Unknown
Karya	KRI	Stream	Ziliana^c^	N39°58′ E22°25′	Hand	2011, 2012	20	2	20	Native
Skotina	SKR	Stream	Ziliana^c^	N39°59′ E22°27′	Hand	2012	20	2	20	Native
Koniskos	KNS	Stream	Pinios	N39°48′ E21°48′	Hand	2011, 2012	17	2	17	Transfer from Krania (last 10 to 15 years)^g^
Loggas	LOG	Lake	Pinios	N39°49′ E21°55′	Hand	2011, 2012	17	2	17	Transfer from Krania (last 10 to 15 years)^g^
Begoritida/Agra	BGR	Lake	Aliakmon	N40°46′ E21°46′ ^b^	Trap (fisherman)	2008, 2009	1	1	-	Transfer from Orhomenos and Edessa Aquaculture station^d^
Palaifyto	PLF	River	Axios	N40°47′ E22°17′	Hand/Trap	2011, 2012	20	2	20	Transfer probably from Begoritida/Agra^g^
Tsivlo	TSV	Lake	Streams of North Peloponnese Beach	N38°04′ E22°13′	Hand	2011, 2012	20	2	20	Transfer from unknown origin ^f,g^
Doxa	DOX	Lake	Streams of North Peloponnese Beach	N37°55′ E22°17′	Hand	2011, 2012	23	2	23	Unknown
Perivoli	PRV	Stream	Pinios	N39°03′ E22°11′	Hand	2011	1	1	-	Native
Kefalovriso	KEF	River	Pinios	N39°53′ E22°04′	Hand-land	2012	1	1	-	Transfer from unknown origin^g^
Krania	KRN	Stream	Aliakmon	N39°51′ E21°18′	Hand	2012	20	2	20	Native
Pertouli	PRT	Stream	Acheloos	N39°32′ E21°28′	Hand	2013	22	1	22	Unknown

^a^based on the National Commission of Water (Government Gazette 1383/8/2-9-10 and 1572/Β/28-9-10).

^b^geographic coordinates obtained from Google earth.

^c^from [114].

^d^from [36].

^e^from [115].

^f^from [116].

^g^from local resident and recreational fisherman

### Mitochondrial amplification and sequencing

Phylogenetic analysis of the noble crayfish was carried out using two mitochondrial markers: 1) a partial 16S sequence using 1471 and 1472 primers [40], and 2) a partial PCR-amplified sequence of the Cytochrome Oxidase subunit I (COI) using the universal LCO1490 and HCO2198 primers [41]. PCR reactions were adapted from Maniatsi et al. for COI [42] and Baxevanis et al. for 16S [43], with the following modifications: 0.25 μl KAPA Taq DNA Polymerase (KAPA Biosystems, South Africa), 2.5 μl 10× KAPA Taq Buffer A (KAPA Biosystems, South Africa), and 0.75 (for 16S) and 1.5 (for COI) mM MgCl2. The PCR thermal profile for COI followed Trontelj et al. [5]. For 16S the annealing temperature was slightly modified (45 °C) from Crandall and Fitzpatrick [40]. Sequencing reactions (both directions) were electrophoresed on a PRISM 3730×l DNA analyzer (Applied Biosystems, Foster City, USA) and prepared by Macrogen Inc. (Seoul, South Korea).

### Mitochondrial analysis

The dataset used for the analysis comprised 37 randomly chosen noble crayfish (from a total of 284 individuals) collected from 21 sites of Greece (one or two individuals per site; Table 1) and sequences retrieved from the GenBank (19 for 16S and 32 for COI [6, 29, 35]; accession numbers DQ320033, KX370092, KF888279 to KF888295 for 16S and AY667146, KX369672, KF888296 to KF888325 for COI; Additional file 1). All produced sequences during this study have been deposited in GenBank (accession numbers KY048193 to KY048202 for 16S and KY067207 to KY067228 for COI; Additional file 1). Identity of amplified regions was confirmed using the BLAST searches. Sequences (16S and COI) were viewed in Bioedit v. 7.2.5 [44] and aligned in ClustalX v. 2.1 [45]. COI sequences were screened for pseudogenes following the procedure described in Buhay [46]. For each mitochondrial marker (COI and 16S) the heterogeneity of nucleotide frequencies, substitution and saturation and presence of phylogenetic signal were checked in DAMBE v. 5.5.29 [47]. The substitution model was determined in PartitionFinder v. 2.1.1 [48] using the Bayesian Information Criterion (BIC). Sequences (16S and COI) derived from the same individual were combined for further analysis (Additional file 1). “Concatenation” approach was followed since: 1) the two loci are linked on the mitochondria and maternally inherited [49], 2) the two genes have different resolving power [50], 3) the concatenation method can perform equally or better than methods that attempt to account for sources of error introduced by incomplete lineage sorting [51], and 4) the selection of one mitochondrial region can influence the results obtained [52].

Phylogenetic inference was performed using a strict clock model and a coalescent tree prior in Beast v. 1.8 [53]. The Markov Chain Monte Carlo (MCMC) analysis run comprised 10^8^ generations, sampled every 10^4^ generations. In order to determine convergence and appropriate burn-in, the Effective Sample Size (ESS) values, densities plots and trace logs for each parameter were visualized in Tracer v. 1.5 [54]. The first 10^6^ generations (10%) were discarded as burn-in. ESS values for all parameters were >486, larger than the threshold value of 200 identified by Tracer v. 1.5. The best fit tree was found using the Maximum clade credibility tree option in the Target tree type implemented in TreeAnnotator v. 1.8. Graphical representation of the relation of the number of haplotypes and samples with the phylogroups, were implemented in R v. 3.1.2 [55]. Pairwise within and between genetic distances of the haplotype groups for 16S and COI (Kimura 2-parameter and p-distance model), were computed in MEGA v. 6.06 [56].

Molecular dating was performed in Beast v. 1.8, using the same parameters as in the phylogenetic inference analysis (see also section “Results” for the best fit models generated for COI and 16S). To our knowledge, there are no fossil records and geophysical events in order to date the phylogroups of *Astacus astacus*. In order to estimate divergence time, different mutation rates were taken into account: 1) mitochondrial Arthropod substitution rate (2.3% pairwise sequence divergence per million years [57]), and 2) Decapod substitution rates for 16S (0.65-0.88% pairwise sequence divergence per million years) and COI (1.66-2.33% pairwise sequence divergence per million years) genes [58]. Both approaches have been previously used in crayfish [6, 35].

### Microsatellite genotyping

The analysis comprised 278 samples from 16 sites [i.e. from the 21 sampled sites, 5 sites were excluded from the subsequent analysis due to low sampling size (*n* < 6); Table 1]. Six microsatellite loci (Aas8, Aas766, Aas1198, Aas2489, Aas3040 and Aas3950) were used [59, 60], with the forward primer IRD800-labelled. The PCR reaction and amplification were modified from those of Kõiv et al. [59, 60] (Additional file 2). Reagents were the same as described in mitochondrial section. In all PCR reactions 0.5 μl 10× Bovine Serum Albumin (BSA) was added.

Microsatellite analysis was performed in a semi-automated Li-COR® 4200 DNA Analyzer (Li-COR, Nebraska, USA), using 6.5% acrylamide sequencing gels (Li-COR®KB Plus™). Data was genotyped in Saga^GT^ software (Li-COR, Nebraska, USA). Allele scoring was facilitated by using the same individuals for each locus as a reference sample between gels, in combination with the molecular genetic marker (50-350 bp, Size Standard IRDye™ 800 Li-COR®). Scoring accuracy was increased by independent genotyping of the samples by two researchers.

### Microsatellite analysis

Genotyping errors were assessed using Micro-Checker v. 2.2.3 [61] (95% CI and 10^4^ repetitions). Mean number of alleles per locus, allele frequencies and observed, expected and unbiased expected Nei’s heterozygosity [62] were calculated using Genetix v. 4.05 [63]. Genepop v. 4.2.2 [64] was used to test linkage disequilibrium, conformity to Hardy-Weinberg Equilibrium for each locus and to infer genetic differentiation for all pairs of sites. For all probability tests, Markov chain method was applied using 10^4^ generations, 20 batches and 5000 iterations per batch. Significance was assessed following the sequential Bonferroni procedure [65]. Allelic richness, number of alleles and Weir and Cockerham’s estimators (F_IT_, F_IS_ and F_ST_) were computed in FSTAT v. 2.9.3.2 [66]. R_ST_Calc v. 2.2 [67] was used to calculate R_ST_ (10^4^ permutations).

Genetic structure was inferred by the Bayesian clustering method implemented in Structure v. 2.3 [68]. The conditions performed were 20 runs for each genetic cluster (K) between 1 and 16 using a 10^5^ burn-in period followed by 2 x 10^6^ MCMC iterations, under an admixture model with independent allele frequencies. The number of K was determined via Structure Harvester [69]. Label Clump v. 1.1.2 [70] and Distruct v. 1.1 [71] were also employed to merge the results and generate the graphical representation of clusters, respectively. Discriminant analysis of principal components (DAPC) [72] in R package Adegenet v. 1.4-2 [73] was also used to assess the number of clusters and the relationships between populations. The number of K and the optimal number of components to be retained was assessed using the find.clusters (BIC) and optim.a.score functions, respectively.

Monmonier’s maximum difference algorithm implemented in Barrier v. 2.2 [74] was employed to identify barriers to gene flow within the data set (all sixteen sites). The procedure using the F_ST_ and D_CE_ described in [75] was followed. The tested number of probable barriers was between 1 and 10. The relationships between F_ST_, Rousset’s distance measure F_ST_/(1- F_ST_), R_ST_ and geographic distances among sites were analysed using Mantel test with 10^4^ randomizations, available at Isolation By Distance Web Service v. 3.23 (IBDWS [76]). Geographic distances were calculated in Geographic Distance Matrix Generator v. 1.2.3 [77].

Effective population size was estimated using ONeSAMP [78], which relies on Approximate Bayesian Computation. The demographic history of noble crayfish was inferred through a hierarchical Bayesian model implemented in MsVar v. 1.3 [79], using the number of genetic clusters derived from the population structure analysis (software Structure v. 2.3). For each genetic cluster, five independent runs were conducted under an exponential model, with different seeds and hyperpriors (Additional file 3). For each data set, 10^5^ steps and 9 x 10^4^ thinning were used. The generation time was considered as per Pianka [80], using the formula t_2_ = (α + ω)/2, where α is the age at maturation and ω the longevity. Since the longevity of the species is debated (see Background) two approaches were used: 1) the sexual maturity of the species (mean 3.5 years) and 2) the most probable longevity of 7.5 years (generation time 5.5). MsVar analysis was performed utilizing Aristotle University of Thessaloniki, Scientific Computing Office, IT Center, Institutional Computer Cluster resources (consuming a total of 91,561 CPU hours and 38 GB RAM capacity). 50% of each chain was discarded as burn-in. The convergence among MCMC runs was assessed visually and via the Gelman & Rubin [81] and Brooks & Gelman [82] statistics in R v. 3.1.2, using the package Coda v. 0.16-1 [83]. Point estimates of less than 1.2 were considered as a good indicator of convergence [84]. Hedge’s d and mean effective size per cluster, along with their 95% CI, were calculated as described in Paz-Vinas et al. [85].

The relative probability of the demographic event was assessed using approximate Bayes Factors (BF). A generation time of 5.5 for the noble crayfish was used in the analysis. Different levels of BF were considered as in Salmona et al. [86]. BFs were computed every 50 years for a time interval between 0 and 12,000 years (Holocene). Additionally, BFs were also computed for equal length intervals (500, 100 and 50 years) covering the past 100,000, 50,000 and 7000 years, respectively. R-scripts were based on those provided by V. Sousa (personal communication) and those included in Paz-Vinas et al. [85].

## Results

### Mitochondrial analysis

#### mtDNA variation

The full dataset comprised 66 concatenated haplotypes derived from the combination of 53 unique haplotypes for COI and 17 unique haplotypes for 16S. In COI alignment no gaps were present, whereas in 16S alignment one or two gaps were observed. The aligned final dataset had a total length of 684 bp, with 334 bp for 16S and 350 bp for COI. The best fit model generated for 16S was HKY + I [87] and for COI was HKY for codon positions 1 and 2, and HKY + Γ for codon position 3. The haplotype diversity was 0.6857 for 16S and 0.985 for COI. The number of variable and parsimony informative sites, were 17 and 11 for 16S and 49 and 32 for COI, respectively. There was no evidence for heterogeneity in base frequencies for neither mtDNA genes (16S: χ^2^ = 1.51, df = 165, *p* > 0.05 and COI: χ^2^ = 9.26, df = 207, *p* > 0.05). The substitution saturation test showed no significant saturation on either gene, since the index of substitution saturation (Iss for 16S = 0.20 and Iss for COI = 0.025) was lower than the critical value (Iss.c for 16S = 0.685 and Iss.c for COI = 0.686), with *T* = 41.104, df = 333, *p* < 0.001 for 16S and *T* = 73.571, df = 349, *p* < 0.001 for COI.

#### Phylogenetic inference

**Fig. 1:**
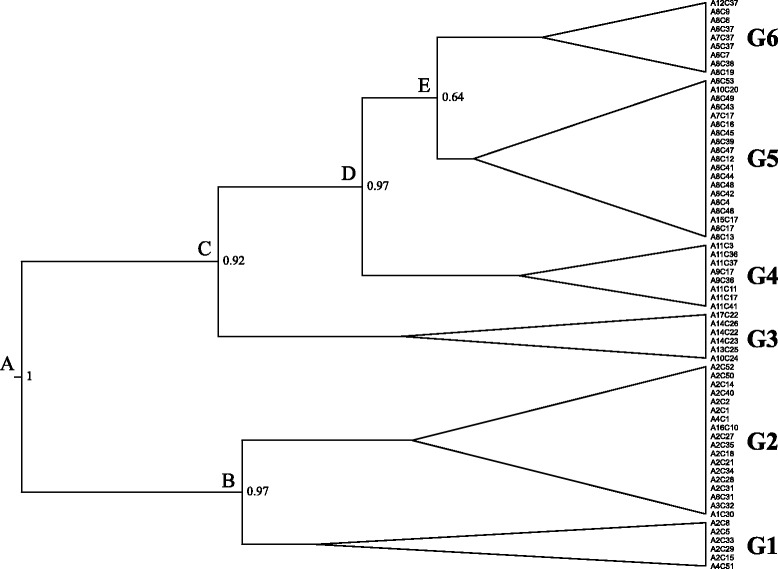
Bayesian inference topology of phylogenetic relationships among 66 concatenated mtDNA haplotypes of the noble crayfish, using Beast v. 1.8. Values at nodes represent posterior probabilities. Major nodes for the noble crayfish are given (**A** to **E**). Phylogenetic clades are represented by symbols from G1 to G6, for more information see Results

The tree inferred from the Bayesian phylogenetic analysis revealed six distinct genetic clades (Fig. 1). Phylogenetic clades G1 and G2 represent haplotypes found in Greece. Lineage G3 consists of haplotypes originated from Croatia, Montenegro and Germany. G4 contains haplotypes from Romania, Kosovo, Czech Republic and Austria. Phylogroup G5 comprises haplotypes belonging to several European countries (Romania, Belgium, Hungary, Bulgaria, Germany, Poland, Finland, Czech Republic and Austria). Finally, phylogenetic clade G6 is composed of Croatian, Montenegrin, German and Romanian haplotypes. It should be noted that minor discrepancies (identified in the shallow branches) have been observed between the independent gene trees produced for 16S and COI (data not shown); however, the major phylogroups were recovered in both trees.

**Fig. 2:**
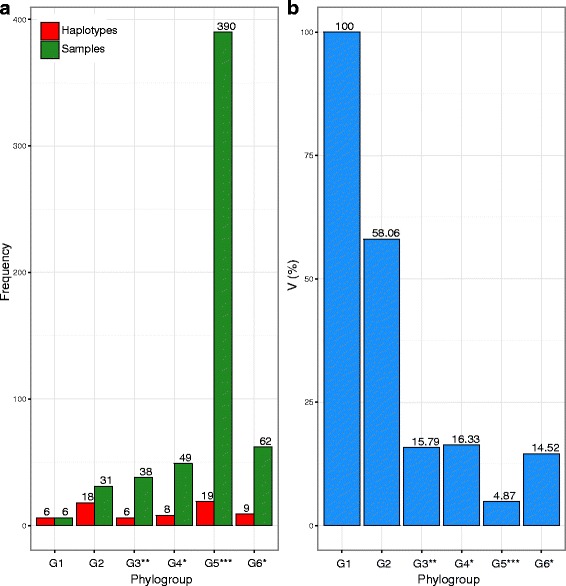
Graphical representation of the composition of the phylogenetic clades (G1 to G6), based on: (**a**) the number of haplotypes (*red color*) and samples (*green color*); (**b**) the percentage of the phylogroup-specific haplotypes divided by the number of individuals (V; *blue color*). Where: *, based on [29]; **, based on [29, 35]; *** based on [6, 29, 35]

All newly identified haplotypes were unique to Greece, and represent 36.4% of the total number of haplotypes (24 out of 66 haplotypes). The percentage of the phylogroup-specific haplotypes divided by the number of individuals (V) was greater than 58.06% in both phylogenetic lineages G1 and G2. In contrast, the percentage V for phylogroups G3 to G6 was between 4.87% and 16.32% (Fig. 2). Furthermore, the between genetic distances of the haplotype groups ranged from 0.1% to 1.9% for 16S and 0.6% to 4.1% for COI. The within genetic distances of the phylogroups were between 0.13% and 1.02% for 16S, and 0.37% and 3.5% for COI (Additional file 4).

### Age estimates

Estimates of divergence times for the concatenated mtDNA dataset showed that the noble crayfish diversified during the Pleistocene. Similar time estimates were produced by the substitution rate approaches (Decapoda and Arthropoda; Table 2). Therefore, estimated time divergences using the mutation rate of Decapoda are further discussed. Node A, represents the separation of the southernmost distribution of *Astacus astacus* (i.e. Greece; phylogroups G1 and G2) from the remaining groups of Europe (G3 to G6) at 1.765 ± 0.0036 MYA (Table 2). The separation of the two Greek haplogroups (G1 and G2) occurred at 1.245 ± 0.0029 MYA (node B). The first group to diversify from the remaining groups of Europe is G3, at 1.284 ± 0.003 MYA (node C). Node D followed (separating G4 from groups G5 and G6) with an estimated divergence time at 0.792 ± 0.0022 MYA. Phylogenetic groups G5 and G6 shared for the last time a common ancestor at 0.556 ± 0.0016 MYA (node E). The divergence within groups (G1 to G6) was estimated at 1.765 ± 0.0036, 0.705 ± 0.0021, 0.761 ± 0.0022, 0.356 ± 0.015, 0.468 ± 0.0014 and 0.285 ± 0.0012 MYA, respectively.

**Table 2:** Estimates of divergence time for the concatenated mtDNA (16S and COI) data set, based on the substitution rate of Decapoda or Arthropoda

Substitution rate	Decapoda			Arthropoda		
Phylogroup or Node	Time	SD	95% HPD	Time	SD	95% HPD
A	1.765	0.0036	(1.1433 - 2.3995)	1.807	0.0049	(0.9810 - 2.7321)
B	1.245	0.0029	(0.7819 - 1.7700)	1.276	0.0037	(0.6481 - 1.9634)
C	1.284	0.0030	(0.7610 - 1.8444)	1.317	0.0040	(0.6953 - 2.1040)
D	0.792	0.0022	(0.4428 - 1.2068)	0.809	0.0028	(0.3496 - 1.3083)
E	0.556	0.0016	(0.3042 - 0.8348)	0.569	0.0019	(0.2598 - 0.9207)
G1	1.048	0.0025	(0.6251 - 1.4989)	1.072	0.0032	(0.5545 - 1.6842)
G2	0.705	0.0021	(0.3464 - 1.1095)	0.727	0.0027	(0.3034 - 1.2180)
G3	0.761	0.0022	(0.3957 - 1.1816)	0.781	0.0028	(0.3307 - 1.2955)
G4	0.356	0.0015	(0.1254 - 0.6256)	0.364	0.0016	(0.1157 - 0.6708)
G5	0.468	0.0014	(0.2453 - 0.7109)	0.478	0.0016	(0.2188 - 0.7847)
G6	0.285	0.0011	(0.1002 - 0.4974)	0.293	0.0013	(0.0854 - 0.5367)

For each phylogenetic group (G1 - G6) and node (A-E) (defined in Fig. 1) the mean divergence time (Time), standard deviation (SD), 95% HPD lower and upper are given

### Microsatellite analysis

#### General summary statistics

Putative null alleles were observed in 3 microsatellite loci and 5 sites (Table 3). Omitting Aas8, Aas2498 or Aas1198 from the analysis did not have any substantial effect on the results (following the procedure described in [39]; data not shown). Therefore, all loci were included in the subsequent analysis. All microsatellite loci were polymorphic, with the number of alleles per locus ranging from 11 (Aas766) to 39 (Aas1198) (Additional file 5). A total of 111 different alleles were recognised with an average of 18.5 alleles per locus (Additional file 5). For all pairs of loci across all sites, 5 out of 240 global tests for genotypic linkage disequilibrium were statistically significant (none after Bonferroni correction) and none among locus pairs (Fisher’s method). Deviation from Hardy-Weinberg equilibrium was present at 9 out of 96 tests (none after Bonferroni correction).

**Table 3:** Population genetic parameters inferred from 6 microsatellite loci for 16 sites of the noble crayfish

	AA1	AA2	KAS	LOG	KNS	KLV	NEO	PLF	KRI	SKR	KRN	KPA	TZA	DOX	TSV	PRT
A	3.667	4.333	8.167	2.500	2.500	4.167	2.833	5.333	2.667	2.167	2.333	2.333	2.500	8.167	7.333	7.333
A_R_	2.844	3.295	5.567	1.969	2.069	2.760	2.281	4.082	2.021	2.056	1.856	2.333	4.833	5.333	5.019	5.080
P_A_	0.000	3.000	5.000	1.000	0.000	1.000	1.000	3.000	1.000	0.000	1.000	0.000	1.000	8.000	8.000	4.000
H_E_	0.468	0.559	0.753	0.204	0.236	0.365	0.299	0.648	0.310	0.310	0.174	0.414	0.641	0.758	0.735	0.761
H_N_	0.480	0.573	0.772	0.210	0.243	0.377	0.314	0.664	0.318	0.318	0.179	0.452	0.700	0.775	0.754	0.779
H_O_	0.408	0.592	0.767	0.265	0.216	0.333	0.356	0.592	0.308	0.300	0.192	0.472	0.667	0.768	0.717	0.727
F_IS_	0.152	−0.033	0.007	−0.271	0.114	0.118	−0.144	0.112	0.030	0.058	−0.074	−0.049	0.051	0.009	0.050	0.067
P_H-W_	0.040	0.103	0.402	0.840	0.344	0.169	0.943	0.047	0.804	0.825	0.850	0.689	0.769	0.069	0.805	0.127
L0	Aas2489	Aas8				Aas8								Aas8		Aas1198, Aas8

Where the number of alleles (A), allelic richness (A_R_), number of private alleles (P_A_), expected heterozygosity (H_E_), unbiased expected Nei’s heterozygosity (H_N_), observed heterozygosity (H_O_), inbreeding coefficient (F_IS_), exact *P*-value for Hardy-Weinberg equilibrium test (P_H-W_) and loci with putative null alleles (L0) are given

The mean allelic richness of each site varied from 1.86 (KRN) to 5.57 (KAS) and the mean observed heterozygosity ranged from 0.19 (KRN) to 0.77 (KAS and DOX) (Table 3; see also Additional file 5). Overall inbreeding coefficient F_IS_ for each sampled site showed heterozygote excess in several sites (AA2, LOG, NEO, KRN and KPA) due to their negative values (between −0.271 in LOG and −0.033 in AA2). Maximum value of F_IS_ was recorded at AA1 (0.152; Table 3). The total number of private alleles was 33. Sites DOX and TSV had the highest number of private alleles with 8 counts, while in AA1, KNS, SKR, KRN and KPA no private alleles were observed.

Exact tests based on allele frequencies for all pairs of sites showed that 6 out of 240 tests were significant (none after Bonferroni correction). The overall genetic heterogeneity was high (F_ST_ = 0.4 and R_ST_ = 0.39), with the estimated values of F_ST_ generally greater than R_ST_ for most of the loci (except Aas3040 and Aas1198; Additional file 6). Pairwise estimates of F_ST_ ranged from −0.0069 (KLV-NEO) to 0.7499 (KRN-SKR) and R_ST_ from −0.0325 (KLV-NEO) to 0.8778 (KRI-KRN) (Additional file 7).

### Population structure

**Fig. 3:**
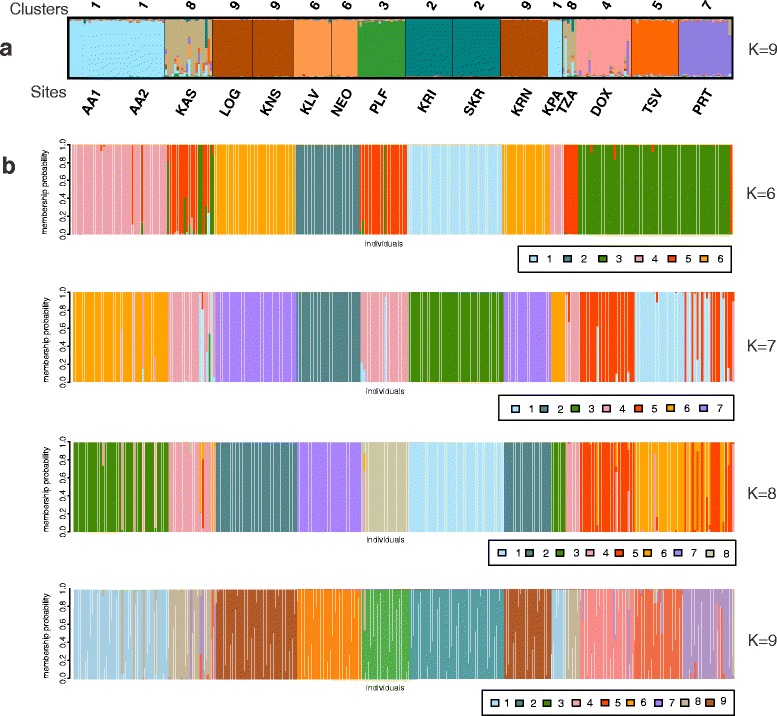
Graphical representation of the clustering analysis, using: (**a**) Structure v. 2.3 (for K = 9); (**b**) R package Adegenet v. 1.4-2 (for K between 6 and 9). In both analyses each bar represents an individual that is colored based on its assignment probability (colors used in both analysis for K = 9 are similar). All K are sorted based on the sampling sites. In (**a**) the abbreviation of the sites (Sites, below) and a number representing the cluster (Clusters 1 to 9, on top) identified are given. In (**b**) for each K identified by the DAPC every cluster is identified by a number (the same numbering is used in Structure)

The inferred number of clusters (K) was nine for the analysis using Structure software (Additional file 8). Sites were grouped together as follows: AA1-AA2-KPA (cluster 1), KRI-SKR (cluster 2), KLV-NEO (cluster 6), KAS-TZA (cluster 8) and LOG-KNS-KRN (cluster 9), while each one of the other sites (PLF, TSV, PRT and DOX) formed different and distinct genetic clusters (cluster 3, 5, 7 and 4, respectively; Fig. 3). The proposed number of clusters for the DAPC analysis varied between 6 and 9 (Additional file 8). It is important to notice that assignment probability for each K identified by DAPC was >0.978 (Additional file 9). In both programs, K = 9 had a similar grouping and was based on the geographic proximity of the sampling sites (Fig. 3).

**Fig. 4:**
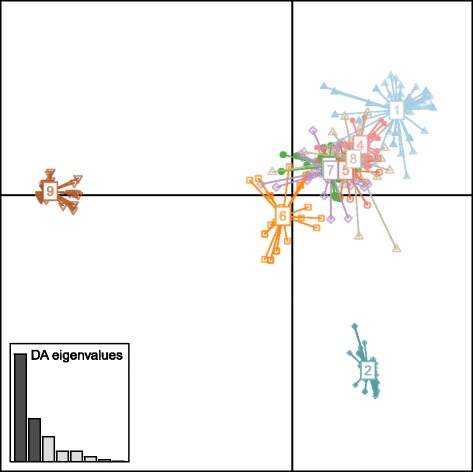
DAPC scatterplot of the first two principal components for K = 9. Clusters are represented by several distinguishable colors (same as in Structure, Fig. 4). The inset shows the discriminant analysis (DA) eigenvalues

The majority of the genetic structure in DAPC for nine clusters was captured in the first principal component (screeplot of the eigenvalues; Fig. 4). Visual inspection of the first two principal components, revealed two distinct genetic clusters (clusters 2 and 9; Fig. 4). Similar results were observed for K between 6 and 8 (Additional file 10). No evidence of isolation by distance was retrieved by Mantel tests (no statistically significant correlation between genetic and geographical distances was observed - Additional file 11).

### Barriers

**Fig. 5:**
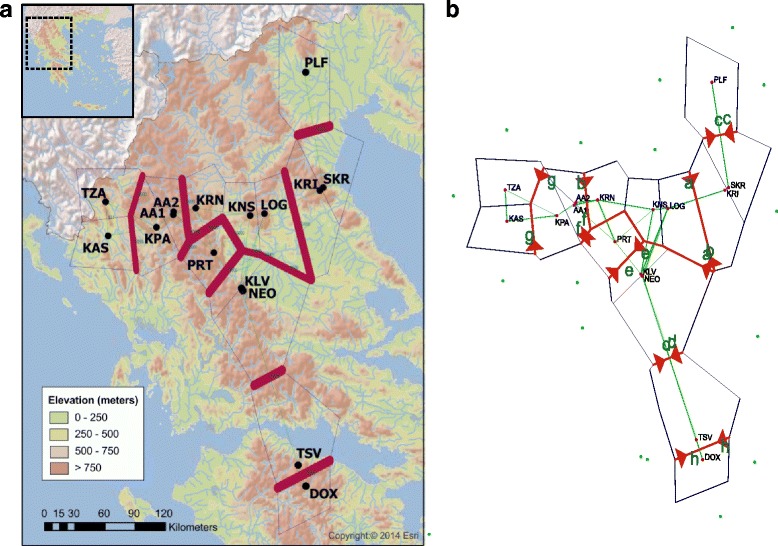
Genetic barriers created via Delaunay triangulation (*green lines*) and Voronoi tessellation (*blue polygons*), as predicted by Barrier software for all sixteen sites. Red lines constitute the genetic barriers detected through (**a**) the bootstrap analysis (10^4^ bootstraps) of D_CE_ and (**b**) the analysis of the pairwise F_ST_ matrix for all microsatellite loci. Circles represent the sites (*black letters*). For (**a**) the thickness of each genetic barrier is proportional to the bootstrap support (*green numbers*). A background map, created using ArcGIS® and ArcMap™ by Esri, is provided in order to visualize the landscape (Copyright © 2014 Esri and its licensors. All rights reserved)

Barrier software revealed the occurrence of breaks in the genetic flow across continental Greece. Analysis of all microsatellite loci simultaneously using D_CE_ or F_ST_ identified eight probable barriers. D_CE_ approach revealed at least 93% bootstrap support for every barrier. The locus by locus analysis of F_ST_ showed that five out of six microsatellite loci (with the exception of Aas3040) agreed with the occurrence of all barriers (Additional file 12). In Aas3040 the observed differences are due to the lack of barriers between sites KRN-LOG-KNS and NEO-KLV, as well as between AA1-AA2-KPA and KAS-TZA. Since the number of data derived from the analysis was large (totally 100; Additional file 12) and the outcomes were similar, it was decided to provide the output data for eight putative barriers, 16 sites and all microsatellite loci using both F_ST_ and D_CE_ (Fig. 5). The derived genetic barriers are closely related with the presence of mountains and river systems, corroborating the findings of structure analysis for K = 9. For instance, sites KRI and SKR are delimited by Mt. Olympus, Mts. Pieria and Aliakmon river. The names of the mountains and major rivers are provided in Additional file 13.

### Demographic history and effective population size

**Fig. 6:**
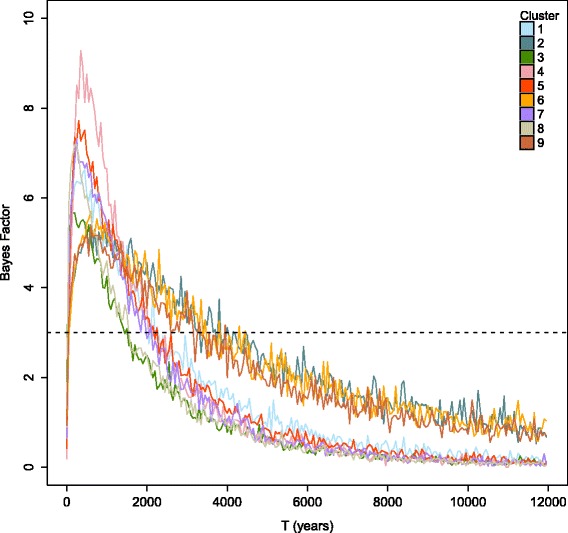
BF values for equal time intervals (100 years) for the past 12,000 years (Holocene). Each genetic cluster (1 to 9) is represented by a different colored-solid line (same as population structure analysis). Horizontal dotted line represents BF values of 3

Point estimates of the genetic clusters varied between 1 and 1.09 (Additional file 14), indicating a good convergence of MCMC runs. MsVar analyses indicated a decline in all genetic clusters, with no apparent overlap of the posterior distributions of past log_10_(N1) and present log_10_(N0) population sizes (Additional file 15). Analyses of the global data set (random samples from different demes were merged into one dataset) gave similar results excluding the presence of a false bottleneck signal (Additional files 14 and 15; as suggested by [56]). Since the descriptive statistics (Table 4) and the statistics describing the magnitude of demographic change (Table 5) are similar for either generation time used (3.5 and 5.5), it was decided to further discus the analysis assuming a generation time of 5.5. The ancestral population size log_10_(N1) among the genetic clusters ranged from 4.97 to 5.51 (Table 4). The contemporary population size log_10_(N0) varied between 0.5 and 1.42 (or mean N0 3.17 and 26.6, respectively) (Table 4). The current effective size estimated for each genetic cluster in ONeSAMP was small, with values varying between 22.069 and 44.191 (Table 6). Mean effective population size (Mean log_10_(N0/N1)) of the genetic clusters decreased by 14 to 20% (average of approximately 17%, Table 5). The magnitude of the bottleneck was strong in every genetic cluster, with negative values for Hedge’s d (between −6.02 and −9.91) (Table 5). Time since the population decline log_10_(T) (Additional file 16) varied between 2.27 and 2.77 among genetic clusters (Table 4). BF analysis indicated that the decline occurred during the last around 5000 years (BF > 3, Fig. 6; see also Additional file 17).

**Table 4:** Mean values of log_10_(N0), log_10_(N1) and log_10_(T) for every genetic cluster (1 to 9) with a generation time of 3.5 and 5.5

Genetic cluster	1		2		3		4		5		6		7		8		9	
Generation time	3.5	5.5	3.5	5.5	3.5	5.5	3.5	5.5	3.5	5.5	3.5	5.5	3.5	5.5	3.5	5.5	3.5	5.5
Mean log_10_(N0)	0.885	1.006	0.522	0.661	0.889	0.781	1.289	1.425	1.298	1.273	0.837	0.582	1.315	1.233	1.411	1.201	0.509	0.501
S.D.	0.711	0.685	0.952	0.826	0.711	0.685	0.827	0.873	0.647	0.519	0.622	0.632	0.856	0.877	0.581	0.655	0.555	0.664
Mean log_10_ (N1)	5.153	5.132	5.355	5.349	4.970	4.971	5.381	5.387	5.502	5.508	5.036	5.272	5.342	5.338	5.462	5.464	5.281	5.269
S.D.	0.382	0.380	0.490	0.495	0.378	0.375	0.324	0.322	0.308	0.308	0.411	0.482	0.333	0.332	0.304	0.303	0.482	0.478
Mean log_10_ (T)	2.286	2.584	2.454	2.770	2.167	2.267	2.358	2.680	2.441	2.611	2.218	2.732	2.430	2.545	2.430	2.432	2.482	2.657
S.D.	0.654	0.631	0.893	0.784	0.755	0.797	0.597	0.489	0.574	0.587	0.786	0.822	0.539	0.604	0.514	0.619	0.826	0.842

Standard Deviation (SD) for every parameter is given

**Table 5:** Statistics describing the magnitude of the demographic change for each genetic cluster (1-9) and generation time (3.5 or 5.5)

Genetic cluster	1		2		3		4		5		6		7		8		9	
Generation time	3.5	5.5	3.5	5.5	3.5	5.5	3.5	5.5	3.5	5.5	3.5	5.5	3.5	5.5	3.5	5.5	3.5	5.5
Mean effective size log_10_(N0/N1)	-1.809	−1.734	−1.976	−1.96	−1.718	−1.764	−1.515	−1.414	−1.576	−1.555	−1.761	−1.993	−1.523	−1.576	−1.449	−1.643	−2.037	-2.030
95% CI lower	−1.809	−1.734	−1.976	−1.96	−1.718	−1.764	−1.515	−1.414	−1.576	−1.555	−1.761	−1.993	−1.523	−1.576	−1.449	−1.643	−2.037	-2.030
95% CI upper	−1.809	−1.734	−1.976	−1.96	−1.718	−1.764	−1.515	−1.414	−1.576	−1.555	−1.761	−1.993	−1.523	−1.576	−1.449	−1.643	−2.037	-2.030
S.D.	0.826	0.804	0.916	0.894	0.838	0.888	0.623	0.468	0.662	0.596	0.863	0.902	0.587	0.686	0.513	0.714	0.930	0.920
Variance	0.682	0.646	0.840	0.800	0.702	0.788	0.388	0.219	0.438	0.356	0.745	0.814	0.344	0.471	0.263	0.51	0.865	0.847
Hedges’ d	−7.479	−7.447	−6.386	−6.885	−7.164	−7.584	−6.516	−6.024	−8.301	−9.915	−7.965	−8.346	−6.199	−6.19	−8.734	−8.347	−9.183	-8.241
95% CI lower	−9.439	−9.407	−8.346	−8.846	−9.124	−9.544	−8.476	−7.984	−10.261	−11.875	−9.925	−10.306	−8.159	−8.15	−10.694	−10.307	−11.143	-10.201
95% CI upper	−5.519	−5.487	−4.426	−4.925	−5.204	−5.623	−4.556	−4.063	−6.341	−7.955	−6.005	−6.386	−4.239	−4.23	−6.774	−6.387	−7.222	−6.281

Mean effective size log_10_(N0/N1), Standard Deviation (SD), Variance, along with Hedges’ d and their 95% Confidence Intervals (CI) are given. Negative values indicate significant bottlenecks

**Table 6:** Mean estimated contemporary size (N0) for each genetic cluster of the noble crayfish, along with their 95% Confidence Intervals (CI) (OneSAMP software)

Cluster	Mean N0	95% CI lower	95% CI upper
1	29.134	20.632	44.899
2	22.069	14.988	37.702
3	23.765	18.996	33.244
4	34.107	27.110	48.675
5	38.318	27.868	61.107
6	34.361	24.070	59.253
7	27.029	22.345	35.878
8	44.191	33.663	65.395
9	23.051	13.907	39.118

## Discussion

### Genetic diversity of the noble crayfish

The observed haplotypic diversity of the noble crayfish (*Astacus astacus*) in Europe (0.6857 for 16S and 0.985 for COI, respectively) is in accordance with the wide geographic range of the species in this area [18]. Six haplotypic lineages (G1 to G6, Fig. 1) were identified using a concatenated mitochondrial data set (16S and COI). The inclusion of samples from the southern Balkans (i.e. Greece) revealed the existence of two novel phylogenetic clades (G1 and G2), separated from the rest of the European haplogroups. The between pairwise genetic distances showed that the phylogroups associated to the southernmost Balkan Peninsula (i.e. Greece, G1 and G2) were clearly differentiated from the remaining European haplotype groups (G3 to G6; Additional file 4). Furthermore, a high number of unique haplotypes in Greece were observed (24 haplotypes from 37 samples; Fig. 2), indicating a high genetic diversity. It must be noted that the number of unique haplotypes and genetic diversity of the southern distribution of *A. astacus* may be biased by the distribution of the sites within each haplogroup and the number of individuals per haplogroup in the Balkan Peninsula. These findings may corroborate the taxonomical division of the noble crayfish, proposed by Karaman [88]; three subspecies are present in Europe, *A. astacus astacus, A. a. colchicus* and *A. a. balcanicus*. Based on our results, it is impelling to suggest that the Greek phylogroups may represent the subspecies *A. a. balcanicus*. Haplotype groups G3 to G6 (Fig. 1) are consistent with a previous study [29]. Additionally, a lower genetic diversity was observed in central and northern Europe (G4 to G6) compared to the Greek (G1 and G2) and the Croatian (G3) lineages (Fig. 2; Additional file 4), probably due to bottleneck and founder effects [16, 17, 29]. Several studies indicated that this pattern could be the result of a post-glacial range expansion from south to north regions [7, 8, 11]. Genetic distances (16S and COI; Additional file 4) were lower to those observed in *Austropotamobius torrentium* in Europe [35], probably reflecting different life-history traits. Phylogroup G5 indicates a pattern typical of a recent range expansion, based on the number of observed haplotypes. This pattern of noble crayfish distribution range is identified as a typical Southern glacial refugium [9]. Therefore, a “Mediterranean” molecular biogeographical pattern [11] and a “grasshopper” [7] or “chub” [8] model of expansion, can be deduced for the noble crayfish. The most likely colonization route of the noble crayfish towards European regions is through the Danube river (as suggested by Schrimpf et al. [29]) and observed, also, in temperate freshwater fishes [8]. In contrast, it has been assumed that the non-endemic Plio-Pleistocene freshwater fishes of Greece originated from further north regions, e.g. the Danube river [89]. The present analysis indicates that Greece is an older glacial refugium than the eastern Black Sea (phylogenetic clade G4) and Western Balkans (phylogenetic clade G3), with the latter two regions already identified by Schrimpf et al. [29]. Nevertheless, the scenario of multiple refugia is confirmed, as suggested for the noble crayfish [17, 29] and postulated for other species (e.g. [90, 91]).

The phylogroups of the noble crayfish diverged during Early and Middle Pleistocene. Specifically, the estimated average divergence times were between ca. 0.3 and 1.8 MYA (Table 2). In the Mediterranean, major cooling events occurred between 2.15 and 2.73 MYA ([92] and references therein), covering the upper HPD limits. During the Middle Pleistocene, two global and prominent climatic transitions seems to be the core of the induced phylogenetic divergence of the species, the “Early Middle Pleistocene Transition” (0.8 to 1.2 MYA) and the “Mid-Brunhes Event” (~0.4 MYA) [93]. Moreover, a correlation can be observed between time of lineage divergence, increased freshwater inputs (last ca. 2.3 MYA [94]) and climatic oscillations (last 1.3 MYA [95]) of the eastern Mediterranean. It has been suggested that there was a connection between the Danube basin and the Greek river basins during the late Pliocene to Pleistocene; this was based on studies related to central European and Danubian freshwater fishes [89]. A geological event that might have influenced the noble crayfish is the opening of the Danube corridor between 0.7 and 1 MYA [96]. In Greece, the delta formation of Pinios river could have, also, affected the noble crayfish genetic structure by dividing the haplotypes into two well-defined clusters (~1.1 MYA; Faugères, 1977 in Caputo [97] and Rook and Martínez-Navarro [98]). It has, also, been suggested that during the glacial-interglacial cycles, freshwater or oligosaline conditions were prevailing in the upper Aegean areas [99]. These events may have played an important role in the expansion of the noble crayfish in northern regions.

### Micro-scale evolution of the noble crayfish

The noble crayfish in Greece is confined in the upper parts of river systems, with a limited, scattered, discontinuous and grouped distribution (21 crayfish sites from a total of 284 investigated sites; Additional file 12). Similar distribution pattern was observed in a former study in Greece [36], but also in other European countries [20, 100]. Anthropogenic activities (agricultural land use and settlements) are negatively affecting the species distribution in riverine systems of the Czech Republic [100]. Agriculture intensification in lowlands of the Mediterranean [101] and water quality of rivers in Greece [102] could explain the distribution pattern of the Greek noble crayfish.

The genetic heterogeneity in the southernmost distribution range of the noble crayfish (i.e. Greece) (global F_ST_ = 0.4 and between sites −0.007 < F_ST_ < 0.75; Additional file 6) was comparable to wider geographical studies (i.e., global F_ST_ = 0.264 [17] and 0.008 < F_ST_ < 0.723 [29]). Structure analysis revealed a high number of genetic clusters (Fig. 3). The higher sub-structuring of Greece compared to central and northern regions of Europe [17, 29] corroborates the findings of the mitochondrial analysis.

The identified nine clusters (K = 9) revealed a relation between them and the geographic landscape of Greece (Figs. 3 and 5). Furthermore, eight putative barriers were inferred revealing that mountains played an important role in the gene flow (Fig. 5). The majority of the inferred clusters (K = 9) also revealed a close relation with their river system, suggesting geographic isolation. These findings are in accordance with field observations on wild populations of noble crayfish (indigenous to the location). Those studies indicated a sedentary nature of the noble crayfish, with low dispersal ability [20, 103]. However, discrepancies can be observed in two clusters (i.e. 1 and 9, Fig. 3), with their components belonging to two different river systems. The most likely explanation is due to human interference. Cluster 1 (sites AA1, KPA and AA2; Fig. 3) may be the result of indirect human activities, since deviation of water by the Aoos hydroelectric power plant has been reported [102]. Cluster 9 is a typical case of a recent human translocation (sites KNS and LOG translocated from site KRN; Fig. 3 and Table 1). In fact, the Geographic Distance Matrix Generator shows that in several cases it involves long-distance translocations (e.g. 53 km in a straight line, between sites KRN and LOG). The same observation was inferred for the river Kalamas, by a former study [36]. It is important to notice, that the sedentary nature of the species depends on food availability, sex, external factors and familiarity of the noble crayfish to the habitat [20, 103]. Low pairwise F_ST_ and R_ST_ values between sites from different river catchments (Additional file 6), absence of correlation between genetic and geographical distances (Additional file 11) and close genetic relation of the majority of the clusters (Fig. 4), indicated that human translocations have shaped the genetic structure of *A. astacus* in Greece (and not, so much, relevant biogeographic events). Information on the status of the species (Table 1) disclosed that several identified distinct clusters are due to human translocation (e.g. cluster 8, Kalamas river).

Person(s) involved in translocation of the noble crayfish in Greece had not followed good conservation practices, such as similarities or dissimilarities of habitats, riverine systems or genetic make-up of the species (e.g. sites KNS, LOG and KRN, Table 1 and Fig. 3). Similar observations are also reported in other European regions [16, 17]. Another issue that must be addressed is the nature of the translocations. Unfortunately, translocations may occur without scientific guidance and/or governmental consensus (for instance sites LOG and KNS; Table 1). The absence of appropriate controls may be an issue, since overharvesting and violation of regulations from local and recreational fishermen is common practice. For example, 671 noble crayfish with highly variable lengths (approximately between 5 and 12 cm Total Length or TL) were collected in a 24 h period in Karya by a local recreational fisherman (personal observation). It should be noted that, in Greece, trading of *A. astacus* is prohibited for less than 7 cm TL (Ministerial Decision A2-3354/2007, Government Gazette 2207/B/14.11.2007), while the minimum allowed fishing length is 10 cm TL (Royal Decree 142/1971, Government Gazette 49A/1971). Similar phenomena have also been observed in Norway [104]. Prohibiting and restricting measures (e.g. restriction of fishing gear) could improve management practices.

Analysis of the demographic history in the southernmost distribution of the species (i.e. Balkan Peninsula, Greece) insinuates a severe decrease in the population size during the Holocene (last ~5000 years), resulting in small effective population sizes (Fig. 6; Tables 4, 5 and 6; Additional files 14, 15 and 16). The culminating arid conditions of the last approximately 5.5 kyr BP [105] may have played an important role in the recent evolutionary history of the noble crayfish (potential and reoccurring bottlenecks). During that period, several climatic oscillations (e.g. [106]) and variations in river flood activity (e.g. [107]) have been reported (see also Additional file 17E). Furthermore, several geological changes influencing freshwater systems of Greece have been recorded during the mid- and late Holocene [97, 108].

However, for the last ~5000 years and due to the advent of the Bronze Age (~3.1-5.3 kyr BP), it is difficult to distinguish climate-induced variations from human-mediated influence on riverine systems of Greece [109, 110]. Historical records showed that from the Bronze Age (~5.3 kyr BP) until nowadays several anthropogenic activities affected the continental Greece. Changes in settlements, hydraulic works (irrigation, e.g. aqueducts, dams, water diversions and creation of artificial lakes) and wars (e.g. [111, 112]) are some of the factors that may have directly or indirectly influenced the noble crayfish populations (potential alteration or destruction of habitats and crayfish consumption by humans). The major hydraulic/engineering project in Copais lake (Central - South Greece) during the Bronze Age by the Mynians (Mycenaean civilization [111]) illustrates the intensity of the human activity in Greece. Notably, it is known that crustaceans are consumed since the Bronze Age [113].

## Conclusions

The noble crayfish revealed a high genetic diversity, with its southernmost range limit playing a crucial role. Mitochondrial analysis (16S and COI) inferred six phylogroups; two of them (corresponding to the southernmost distribution of the species) were identified for the first time. Genetic analyses revealed that the genetic diversity of *A. astacus* populations from Southern Balkans (Greece) is high and they form a distinct group when compared with their European counterparts. The divergence time analysis implied a potential differentiation of the species during the Early and Middle Pleistocene (between ca. 0.3 and 1.8 MYA). During that period, several Pleistocene climatic oscillations and geological processes corroborate the inferred divergence. Therefore, we consider that the area of the Southern Balkans may have played an important role in shaping the genetic make-up of *A. astacus* populations (old centre of expansion and/or southern glacial refugium). Microsatellite analysis focusing on the noble crayfish from its southernmost distribution (Balkan Peninsula, Greece) disclosed six to nine distinct clusters. The combination of the inferred genetic clusters with the genetic barriers and the status of each sampled site (natural or translocated) unraveled the influence of several factors. Specifically, it was deduced that the landscape of the region (mountains and river systems) and human related interventions (mostly translocations) were the main factors affecting the genetic structure of the noble crayfish. Finally, it was figured out that during mid to late Holocene, a severe decrease in the effective population size of the Greek noble crayfish took place. During that time period, several climatic, geological and anthropogenic processes occurred influencing the distribution of noble crayfish in the southernmost Balkan Peninsula (i.e. Greece). It is concluded that the noble crayfish has been primarily influenced by physical processes (climate and geology), while recently, anthropogenic implications (mainly translocations by humans and niche degradation) may have affected its genetic and geographic structure.

## Additional files

Additional file 1: List of sequences used in the mitochondrial analysis. A detailed table with the information of each sequence used is given. The information comprises the name of the site, country of origin, haplotype, GenBank Accession numbers and bibliographic references. (DOC 362 kb)

Additional file 2: The modified conditions used for the PCR and amplification of each microsatellite loci are given. (DOC 32 kb)

Additional file 3: Starting values (α, σ, β and τ) for hyperpriors (log(N0), log(N1), log(Θ) and log(T)) for each independent MCMC run. (DOC 30 kb)

Additional file 4: Pairwise within and between genetic distances of the haplotype groups (G1 to G6) for COI and 16S. (DOC 56 kb)

Additional file 5: Genetic diversity of the microsatellite loci and sampling sites. Table with the number of alleles, allelic richness, number of private alleles, expected heterozygosity, unbiased expected Nei’s heterozygosity, observed heterozygosity, inbreeding coefficient and exact *P*-value for Hardy-Weinberg equilibrium test are listed for the microsatellite loci and the sampling sites. (DOC 140 kb)

Additional file 6: Fixation indices for all microsatellite loci. Values of the fixation indices for all microsatellite loci based on infinite allele model (F_IT_, F_IS_ and F_ST_) and stepwise mutation model (R_ST_). (DOC 30 kb)

Additional file 7: Estimated pairwise F_ST_ and R_ST_ values based on sampling sites. (DOC 53 kb)

Additional file 8: Inference of number of clusters. The file contains graphical representations of a) DeltaK for each K (1 to 16) produced by Structure Harvester and b) Bayesian information criterion (BIC) for every number of clusters, using DAPC. (DOC 108 kb)

Additional file 9: Assignment probability (K between 6 and 9) and assignment probability per cluster, as identified by DAPC analysis. (DOC 35 kb)

Additional file 10: Graphical representations of the DAPC scatterplots of the first two principal components for K between 6 and 8. (DOC 325 kb)

Additional file 11: Mantel test correlation between geographical distance (kilometers) and genetic distances (given as F_ST_, F_ST_/(1-F_ST_) or R_ST_) for all sites. (DOC 637 kb)

Additional file 12: Graphical representation of genetic barriers (1 to 10) based on: a) sixteen sampling stations, all microsatellites loci and the genetic distance D_CE_, b) sixteen sampling stations, all microsatellites loci and the F_ST_, c) sixteen sampling stations, each microsatellite locus (Aas8, Aas766, Aas1198, Aas2498, Aas3040 and Aas3950) and F_ST_, d) nine genetic clusters (of the Structure software), all microsatellites loci and the genetic distance D_CE_, and e) nine genetic clusters (of the Structure software), all microsatellites loci and the F_ST_. (DOC 10235 kb)

Additional file 13: Map of the sampled noble crayfish in Greece (Copyright © 2014 Esri and its licensors. All rights reserved) where abbreviation of the sampling sites, names of major rivers, mountains and sierra, type of genetic markers used (mitochondrial and/or nuclear; mtDNA and nDNA, respectively) and elevation are given. (DOC 2355 kb)

Additional file 14: Point estimates with their upper Confidence Interval (CI) for all parameters (log(N0), log(N1), log(Θ) and log(T)) and every genetic cluster for a generation time of 3.5 and 5.5. (DOC 48 kb)

Additional file 15: Posteriors and priors densities plots for the past (N1) and present (N0) population sizes of each genetic cluster (cluster 1 to 9) and the global data set (samples from different demes were merged into one dataset; black color). (DOC 859 kb)

Additional file 16: Posteriors and priors densities plots for time since the population decline (T) for each genetic cluster (cluster 1 to 9) and the global data set (samples from different demes were merged into one dataset; black color). (DOC 674 kb)

Additional file 17: The relative probability of different hypotheses was assessed using approximate Bayes Factors (BF). A generation time of 5.5 for the noble crayfish was used in the analysis. Hypotheses were examined for the past 12,000 and 7000 years. (DOC 2096 kb)

## Abbreviations

BF: Bayes factors
BIC: Bayesian information criterion
CI: Confidence interval
COI: Cytochrome oxidase subunit I
DA: Discriminant analysis
DAPC: Discriminant analysis of principal components
ESS: Effective sample size
HPD: Highest posterior density
Iss: Index of substitution saturation
Iss.c: Index of substitution saturation critical value
IT: Information technology
K: Number of clusters
MCMC: Markov chain Monte Carlo
mtDNA: Mitochondrial DNA
PCR: Polymerase chain reaction
TL: Total length
V: Percentage of the phylogroup-specific haplotypes divided by the number of individuals
