# Supplementary material for: Microevolution of the noble crayfish (Astacus astacus) in the Southern Balkan Peninsula
Source: BMC Evol Biol. 2017 May 30;17:122. doi: 10.1186/s12862-017-0971-6 (PMC5450353; doi:10.1186/s12862-017-0971-6)
Supplement: Supplementary file 1 — List of sequences used in the mitochondrial analysis. A detailed table with the information of each sequence used is given. The information comprises the name of the site, country of origin, haplotype, GenBank Accession numbers and bibliographic references. (DOC 362 kb) [file 12862_2017_971_MOESM1_ESM.doc]

# Additional file 1

List of sequences used in the mitochondrial analysis. Site, country of origin (Country), haplotype (H) and GenBank Accession numbers (Ac.) for every haplotype are given. Abbreviations are used for countries: “AUS” for Austria, “BEL” for Belgium, “BUL” for Bulgaria, “CZR” for Czech Republic, “CRO” for Croatia, “FIN” for Finland, “GRE” for Greece, “GER” for Germany, “HUN” for Hungary, “KSV” for Kosovo, “MON” for Montenegro, “NOR” for Norway, “POL” for Poland, “ROM” for Romania and “?” for Unknown.

| **Site** | **Country** | **NSI** | **Ac. 16S** | **Ac. COI** | **16S H** | **COI H** | **Concatenated H** | **Bibliography** |
| --- | --- | --- | --- | --- | --- | --- | --- | --- |
| Begoritida/Agra | Gr | 1 | KY048194 | KY067213 | A2 | C15 | A2C15 | present study |
| Kalamas | Gr | 1 | KY048194 | KY067218 | A2 | C29 | A2C29 | present study |
| Kalamas | Gr | 1 | KY048194 | KY067222 | A2 | C33 | A2C33 | present study |
| Chani Kaber Aga | Gr | 1 | KY048197 | KY067215 | A2 | C21 | A2C21 | present study |
| Chani Kaber Aga | Gr | 1 | KY048194 | KY067215 | A2 | C21 | A2C21 | present study |
| Palaifyto | Gr | 1 | KY048197 | KY067217 | A2 | C28 | A2C28 | present study |
| Palaifyto | Gr | 1 | KY048194 | KY067223 | A2 | C34 | A2C34 | present study |
| Kalivia | Gr | 1 | KY048198 | KY067219 | A1 | C30 | A1C30 | present study |
| Kalivia | Gr | 1 | KY048194 | KY067224 | A2 | C35 | A2C35 | present study |
| Arahthos | Gr | 1 | KY048199 | KY067215 | A2 | C21 | A2C21 | present study |
| Pertouli | Gr | 1 | KY048200 | KY067207 | A2 | C1 | A2C1 | present study |
| Perivoli | Gr | 1 | KY048193 | KY067211 | A16 | C10 | A16C10 | present study |
| Kefalovriso | Gr | 1 | KY048201 | KY067221 | A3 | C32 | A3C32 | present study |
| Skotina | Gr | 2 | KY048194 | KY067207 | A2 | C1 | A2C1 | present study |
| Fragkades | Gr | 2 | KY048194 | KY067212 | A2 | C14 | A2C14 | present study |
| Doxa | Gr | 1 | KY048195 | KY067208 | A2 | C2 | A2C2 | present study |
| Doxa | Gr | 1 | KY048194 | KY067208 | A2 | C2 | A2C2 | present study |
| Neochori | Gr | 2 | KY048194 | KY067216 | A2 | C27 | A2C27 | present study |
| Koniskos | Gr | 2 | KY048194 | KY067220 | A2 | C31 | A2C31 | present study |
| Krania | Gr | 1 | KY048194 | KY067220 | A6 | C31 | A6C31 | present study |
| Krania | Gr | 1 | KY048200 | KY067220 | A2 | C31 | A2C31 | present study |
| Karya | Gr | 1 | KY048202 | KY067207 | A2 | C1 | A2C1 | present study |
| Karya | Gr | 1 | KY048194 | KY067225 | A2 | C40 | A2C40 | present study |
| Aoo1 | Gr | 1 | KY048194 | KY067209 | A2 | C5 | A2C5 | present study |
| Aoo1 | Gr | 1 | KY048194 | KY067210 | A2 | C8 | A2C8 | present study |
| Aoo2 | Gr | 1 | KY048194 | KY067215 | A2 | C21 | A2C21 | present study |
| Aoo2 | Gr | 1 | KY048194 | KY067226 | A2 | C50 | A2C50 | present study |
| Tzaravina | Gr | 1 | KY048194 | KY067228 | A2 | C52 | A2C52 | present study |
| Tzaravina | Gr | 1 | KY048194 | KY067214 | A2 | C18 | A2C18 | present study |
| Loggas | Gr | 1 | KY048194 | KY067220 | A2 | C31 | A2C31 | present study |
| Loggas | Gr | 1 | KY048195 | KY067220 | A2 | C31 | A2C31 | present study |
| Tsivlo | Gr | 1 | KY048196 | KY067207 | A4 | C1 | A4C1 | present study |
| Tsivlo | Gr | 1 | KY048196 | KY067227 | A4 | C51 | A4C51 | present study |
| Rakov Creek | Cr | 1 | KF888295 | KF888325 | A10 | C24 | A10C24 | [29] |
| Ričica River | Cr | 1 | KF888294 | KF888324 | A13 | C25 | A13C25 | [29] |
| Jäglitz | Ge | 2 | KF888292 | KF888321 | A14 | C22 | A14C22 | [29] |
| Dielbach (Woog) | Ge | 1 | KF888292 | KF888321 | A14 | C22 | A14C22 | [29] |
| Vukovina Lake | Cr | 5 | KF888292 | KF888321 | A14 | C22 | A14C22 | [29] |
| Bačica Creek | Cr | 3 | KF888293 | KF888321 | A14 | C22 | A14C22 | [29] |
| Ježevo Lake, Velika Gorica | Cr | 1 | KF888293 | KF888321 | A14 | C22 | A14C22 | [29] |
| Pakra River, Kusonje | Cr | 1 | KF888293 | KF888321 | A14 | C22 | A14C22 | [29] |
| Ribnjak Creek, Vladisovo | Cr | 2 | KF888293 | KF888321 | A14 | C22 | A14C22 | [29] |
| Trećak Creek Staro Petrovo Selo | Cr | 2 | KF888293 | KF888321 | A14 | C22 | A14C22 | [29] |
| Subocka River | Cr | 1 | KF888293 | KF888321 | A14 | C22 | A14C22 | [29] |
| Šumetlica Creek | Cr | 3 | KF888293 | KF888321 | A14 | C22 | A14C22 | [29] |
| Zeta River | Mo | 4 | KF888293 | KF888321 | A14 | C22 | A14C22 | [29] |
| Liverovići Lake | Mo | 1 | KF888293 | KF888321 | A14 | C22 | A14C22 | [29] |
| Paklenica River - NP Paklenica | Cr | 2 | KF888293 | KF888321 | A14 | C22 | A14C22 | [29] |
| Bačica Creek | Cr | 1 | KF888293 | KF888323 | A14 | C23 | A14C23 | [29] |
| Bašnica River, Gračac | Cr | 2 | KF888293 | KF888322 | A14 | C26 | A14C26 | [29] |
| Borovik Lake | Cr | 1 | KF888293 | KF888322 | A14 | C26 | A14C26 | [29] |
| Dubočanka River | Cr | 1 | KF888293 | KF888322 | A14 | C26 | A14C26 | [29] |
| Krapina River | Cr | 1 | KF888293 | KF888322 | A14 | C26 | A14C26 | [29] |
| Ribnjak Creek, Vladisovo | Cr | 1 | KF888293 | KF888322 | A14 | C26 | A14C26 | [29] |
| Allna | Ge | 1 | KF888285 | KF888296 | A9 | C17 | A9C17 | [29] |
| Ciornovãt | Ro | 6 | KF888285 | KF888311 | A9 | C36 | A9C36 | [29] |
| U sudu | Cz | 2 | KF888286 | KF888313 | A10 | C20 | A10C20 | [29] |
| Freundsheimer Weiher | Au | 1 | KF888286 | KF888313 | A10 | C20 | A10C20 | [29] |
| Maierus | Ro | 1 | KF888284 | KF888312 | A11 | C11 | A11C11 | [29] |
| Bogata | Ro | 1 | KF888284 | KF888312 | A11 | C11 | A11C11 | [29] |
| Moravita | Ro | 1 | KF888284 | KF888296 | A11 | C17 | A11C17 | [29] |
| Valea Adanca | Ro | 1 | KF888284 | KF888310 | A11 | C3 | A11C3 | [29] |
| Natra | Ro | 1 | KF888284 | KF888311 | A11 | C36 | A11C36 | [29] |
| Comarnic | Ro | 1 | KF888284 | KF888311 | A11 | C36 | A11C36 | [29] |
| Toplita | Ro | 1 | KF888284 | KF888311 | A11 | C36 | A11C36 | [29] |
| Ravistea | Ro | 8 | KF888284 | KF888311 | A11 | C36 | A11C36 | [29] |
| Moravita | Ro | 3 | KF888284 | KF888311 | A11 | C36 | A11C36 | [29] |
| Dognecea | Ro | 1 | KF888284 | KF888311 | A11 | C36 | A11C36 | [29] |
| Ciornovãt | Ro | 1 | KF888284 | KF888311 | A11 | C36 | A11C36 | [29] |
| Caras | Ro | 1 | KF888284 | KF888311 | A11 | C36 | A11C36 | [29] |
| Taraia | Ro | 1 | KF888284 | KF888311 | A11 | C36 | A11C36 | [29] |
| Iazul | Ro | 1 | KF888284 | KF888311 | A11 | C36 | A11C36 | [29] |
| Sinca | Ro | 1 | KF888284 | KF888311 | A11 | C36 | A11C36 | [29] |
| Hartibaciu | Ro | 1 | KF888284 | KF888311 | A11 | C36 | A11C36 | [29] |
| Venetia | Ro | 1 | KF888284 | KF888311 | A11 | C36 | A11C36 | [29] |
| Simbrezi | Ro | 2 | KF888284 | KF888311 | A11 | C36 | A11C36 | [29] |
| Rupea | Ro | 4 | KF888284 | KF888311 | A11 | C36 | A11C36 | [29] |
| Oituz | Ro | 1 | KF888284 | KF888311 | A11 | C36 | A11C36 | [29] |
| Unnamed stream Ferizaj (Uroševac) | Ko | 1 | KF888287 | KF888311 | A11 | C36 | A11C36 | [29] |
| Clocotici | Ro | 7 | KF888284 | KF888315 | A11 | C37 | A11C37 | [29] |
| Clocotici | Ro | 1 | KF888284 | KF888319 | A11 | C41 | A11C41 | [29] |
| Valkeinen | Fi | 2 | KF888281 | KF888296 | A7 | C17 | A7C17 | [29] |
| Farm Augsburg | Ge | 1 | KF888281 | KF888296 | A7 | C17 | A7C17 | [29] |
| Rosko | Po | 2 | KF888283 | KF888296 | A15 | C17 | A15C17 | [29] |
| Dielbach (Woog) | Ge | 1 | KF888279 | KF888299 | A8 | C12 | A8C12 | [29] |
| Wolfsägertal | Ge | 1 | KF888279 | KF888299 | A8 | C12 | A8C12 | [29] |
| Razdvec | Bu | 1 | KF888279 | KF888305 | A8 | C13 | A8C13 | [29] |
| Farm Augsburg | Ge | 1 | KF888279 | KF888301 | A8 | C16 | A8C16 | [29] |
| Koppelsee | Ge | 3 | KF888279 | KF888296 | A8 | C17 | A8C17 | [29] |
| Tonkuhle | Ge | 3 | KF888279 | KF888296 | A8 | C17 | A8C17 | [29] |
| Rakowe Duze | Po | 5 | KF888279 | KF888296 | A8 | C17 | A8C17 | [29] |
| Seki | Po | 5 | KF888279 | KF888296 | A8 | C17 | A8C17 | [29] |
| Bez Nazwu | Po | 5 | KF888279 | KF888296 | A8 | C17 | A8C17 | [29] |
| Czarne | Po | 5 | KF888279 | KF888296 | A8 | C17 | A8C17 | [29] |
| Rosko | Po | 2 | KF888279 | KF888296 | A8 | C17 | A8C17 | [29] |
| Tomczyna | Po | 5 | KF888279 | KF888296 | A8 | C17 | A8C17 | [29] |
| Lake1 close to Miastko | Po | 5 | KF888279 | KF888296 | A8 | C17 | A8C17 | [29] |
| Lake2 close to Miastko | Po | 5 | KF888279 | KF888296 | A8 | C17 | A8C17 | [29] |
| Lake3 close to Miastko | Po | 5 | KF888279 | KF888296 | A8 | C17 | A8C17 | [29] |
| Valkeinen | Fi | 3 | KF888279 | KF888296 | A8 | C17 | A8C17 | [29] |
| Yla | Fi | 5 | KF888279 | KF888296 | A8 | C17 | A8C17 | [29] |
| Gut Rietberg | Ge | 4 | KF888279 | KF888296 | A8 | C17 | A8C17 | [29] |
| Stepenitz | Ge | 5 | KF888279 | KF888296 | A8 | C17 | A8C17 | [29] |
| Schwarze Elster | Ge | 6 | KF888279 | KF888296 | A8 | C17 | A8C17 | [29] |
| Jäglitz | Ge | 5 | KF888279 | KF888296 | A8 | C17 | A8C17 | [29] |
| Svetlohor | Cz | 8 | KF888279 | KF888296 | A8 | C17 | A8C17 | [29] |
| U sudu | Cz | 4 | KF888279 | KF888296 | A8 | C17 | A8C17 | [29] |
| Kramata | Cz | 7 | KF888279 | KF888296 | A8 | C17 | A8C17 | [29] |
| Florenville | Be | 5 | KF888279 | KF888296 | A8 | C17 | A8C17 | [29] |
| Libramont | Be | 2 | KF888279 | KF888296 | A8 | C17 | A8C17 | [29] |
| Aar | Ge | 5 | KF888279 | KF888296 | A8 | C17 | A8C17 | [29] |
| Allna | Ge | 3 | KF888279 | KF888296 | A8 | C17 | A8C17 | [29] |
| Ambach | Ge | 5 | KF888279 | KF888296 | A8 | C17 | A8C17 | [29] |
| Mühlgraben Caldern | Ge | 2 | KF888279 | KF888296 | A8 | C17 | A8C17 | [29] |
| Dautphe | Ge | 4 | KF888279 | KF888296 | A8 | C17 | A8C17 | [29] |
| Dielbach (Woog) | Ge | 3 | KF888279 | KF888296 | A8 | C17 | A8C17 | [29] |
| Donsbach | Ge | 5 | KF888279 | KF888296 | A8 | C17 | A8C17 | [29] |
| Eichelbach | Ge | 5 | KF888279 | KF888296 | A8 | C17 | A8C17 | [29] |
| Fischbach | Ge | 3 | KF888279 | KF888296 | A8 | C17 | A8C17 | [29] |
| Fohnbach | Ge | 5 | KF888279 | KF888296 | A8 | C17 | A8C17 | [29] |
| Giebelsbach | Ge | 1 | KF888279 | KF888296 | A8 | C17 | A8C17 | [29] |
| Gansbach | Ge | 5 | KF888279 | KF888296 | A8 | C17 | A8C17 | [29] |
| Geierstein/Roth | Ge | 10 | KF888279 | KF888296 | A8 | C17 | A8C17 | [29] |
| Waldteich, Irrschelde | Ge | 5 | KF888279 | KF888296 | A8 | C17 | A8C17 | [29] |
| Kallenbach | Ge | 5 | KF888279 | KF888296 | A8 | C17 | A8C17 | [29] |
| Lasterbach | Ge | 5 | KF888279 | KF888296 | A8 | C17 | A8C17 | [29] |
| Merzkrebse | Ge | 5 | KF888279 | KF888296 | A8 | C17 | A8C17 | [29] |
| Meerbach | Ge | 1 | KF888279 | KF888296 | A8 | C17 | A8C17 | [29] |
| Madenmühlen | Ge | 5 | KF888279 | KF888296 | A8 | C17 | A8C17 | [29] |
| Mademühlen 2 | Ge | 4 | KF888279 | KF888296 | A8 | C17 | A8C17 | [29] |
| Nanzenbach | Ge | 1 | KF888279 | KF888296 | A8 | C17 | A8C17 | [29] |
| Klausbach | Ge | 3 | KF888279 | KF888296 | A8 | C17 | A8C17 | [29] |
| Mahlscheid | Ge | 6 | KF888279 | KF888296 | A8 | C17 | A8C17 | [29] |
| Perf | Ge | 5 | KF888279 | KF888296 | A8 | C17 | A8C17 | [29] |
| Hartmann/Rehbach | Ge | 5 | KF888279 | KF888296 | A8 | C17 | A8C17 | [29] |
| Pollichia Woog2 | Ge | 1 | KF888279 | KF888296 | A8 | C17 | A8C17 | [29] |
| Wolfsägertal | Ge | 2 | KF888279 | KF888296 | A8 | C17 | A8C17 | [29] |
| Meisertalweiher | Ge | 5 | KF888279 | KF888296 | A8 | C17 | A8C17 | [29] |
| Saarbach (Lagerweiher) | Ge | 1 | KF888279 | KF888296 | A8 | C17 | A8C17 | [29] |
| Saarbach (Woog) | Ge | 4 | KF888279 | KF888296 | A8 | C17 | A8C17 | [29] |
| Salzbach | Ge | 5 | KF888279 | KF888296 | A8 | C17 | A8C17 | [29] |
| Eifel | Ge | 1 | KF888279 | KF888296 | A8 | C17 | A8C17 | [29] |
| Spielberg | Ge | 2 | KF888279 | KF888296 | A8 | C17 | A8C17 | [29] |
| Steinbruch Rot | Ge | 5 | KF888279 | KF888296 | A8 | C17 | A8C17 | [29] |
| Stippbach | Ge | 5 | KF888279 | KF888296 | A8 | C17 | A8C17 | [29] |
| Waldteich bei Wallenfels | Ge | 4 | KF888279 | KF888296 | A8 | C17 | A8C17 | [29] |
| Waldteich bei Wallenfels | Ge | 1 | KF888280 | KF888296 | A8 | C17 | A8C17 | [29] |
| Breitweiher/Rhön | Ge | 2 | KF888279 | KF888296 | A8 | C17 | A8C17 | [29] |
| Ocherbach | Ge | 5 | KF888279 | KF888296 | A8 | C17 | A8C17 | [29] |
| Urff | Ge | 4 | KF888279 | KF888296 | A8 | C17 | A8C17 | [29] |
| Razdvec | Bu | 4 | KF888279 | KF888296 | A8 | C17 | A8C17 | [29] |
| Gorna Trape | Bu | 1 | KF888279 | KF888296 | A8 | C17 | A8C17 | [29] |
| Freundsheimer Weiher | Au | 2 | KF888279 | KF888296 | A8 | C17 | A8C17 | [29] |
| Wielenbach | Ge | 2 | KF888279 | KF888296 | A8 | C17 | A8C17 | [29] |
| Kádárta | Hu | 3 | KF888279 | KF888296 | A8 | C17 | A8C17 | [29] |
| Schiopu | Ro | 1 | KF888279 | KF888296 | A8 | C17 | A8C17 | [29] |
| Valea Boului | Ro | 1 | KF888279 | KF888296 | A8 | C17 | A8C17 | [29] |
| Barcau | Ro | 1 | KF888279 | KF888296 | A8 | C17 | A8C17 | [29] |
| Valea Stoiaca | Ro | 1 | KF888279 | KF888296 | A8 | C17 | A8C17 | [29] |
| Nadas | Ro | 8 | KF888279 | KF888296 | A8 | C17 | A8C17 | [29] |
| Buhui | Ro | 2 | KF888279 | KF888296 | A8 | C17 | A8C17 | [29] |
| Moravita | Ro | 1 | KF888279 | KF888296 | A8 | C17 | A8C17 | [29] |
| Dognecea | Ro | 1 | KF888279 | KF888296 | A8 | C17 | A8C17 | [29] |
| Caianu | Ro | 1 | KF888279 | KF888296 | A8 | C17 | A8C17 | [29] |
| Bacaia | Ro | 2 | KF888279 | KF888296 | A8 | C17 | A8C17 | [29] |
| Balsa | Ro | 1 | KF888279 | KF888296 | A8 | C17 | A8C17 | [29] |
| Tamasesti | Ro | 4 | KF888279 | KF888296 | A8 | C17 | A8C17 | [29] |
| Balsa | Ro | 3 | KF888279 | KF888296 | A8 | C17 | A8C17 | [29] |
| Cladovita | Ro | 2 | KF888279 | KF888296 | A8 | C17 | A8C17 | [29] |
| Geoagiu | Ro | 1 | KF888279 | KF888296 | A8 | C17 | A8C17 | [29] |
| Conop | Ro | 1 | KF888279 | KF888296 | A8 | C17 | A8C17 | [29] |
| Valea Crisului | Ro | 4 | KF888279 | KF888296 | A8 | C17 | A8C17 | [29] |
| Crisul | Ro | 3 | KF888279 | KF888296 | A8 | C17 | A8C17 | [29] |
| Giacas | Ro | 2 | KF888279 | KF888296 | A8 | C17 | A8C17 | [29] |
| Solocma | Ro | 3 | KF888279 | KF888296 | A8 | C17 | A8C17 | [29] |
| Niraj | Ro | 1 | KF888279 | KF888296 | A8 | C17 | A8C17 | [29] |
| Valea Adanca | Ro | 1 | KF888279 | KF888296 | A8 | C17 | A8C17 | [29] |
| Bichigiu | Ro | 1 | KF888279 | KF888296 | A8 | C17 | A8C17 | [29] |
| Agrij | Ro | 1 | KF888279 | KF888296 | A8 | C17 | A8C17 | [29] |
| Tetisu | Ro | 1 | KF888279 | KF888296 | A8 | C17 | A8C17 | [29] |
| Poiana | Ro | 1 | KF888279 | KF888296 | A8 | C17 | A8C17 | [29] |
| Sarasau | Ro | 1 | KF888279 | KF888296 | A8 | C17 | A8C17 | [29] |
| Valea Tejei | Ro | 1 | KF888279 | KF888296 | A8 | C17 | A8C17 | [29] |
| Valea Holita | Ro | 1 | KF888279 | KF888296 | A8 | C17 | A8C17 | [29] |
| Valea Mare | Ro | 1 | KF888279 | KF888296 | A8 | C17 | A8C17 | [29] |
| Zoo Zajak | Ge | 5 | KF888279 | KF888296 | A8 | C17 | A8C17 | [29] |
| Farm in Belgium | Be | 1 | KF888279 | KF888296 | A8 | C17 | A8C17 | [29] |
| Farm Augsburg | Ge | 8 | KF888279 | KF888296 | A8 | C17 | A8C17 | [29] |
| Langsee | Ge | 8 | KF888279 | KF888297 | A8 | C39 | A8C39 | [29] |
| Farm Oeversee | Ge | 4 | KF888279 | KF888297 | A8 | C39 | A8C39 | [29] |
| Razdvec | Bu | 1 | KF888279 | KF888306 | A8 | C4 | A8C4 | [29] |
| Nadas | Ro | 1 | KF888279 | KF888319 | A8 | C41 | A8C41 | [29] |
| Farm in Belgium | Be | 1 | KF888279 | KF888319 | A8 | C41 | A8C41 | [29] |
| Rosko | Po | 1 | KF888279 | KF888309 | A8 | C42 | A8C42 | [29] |
| Barthe | Ge | 5 | KF888279 | KF888307 | A8 | C43 | A8C43 | [29] |
| Beli Osam | Bu | 4 | KF888279 | KF888304 | A8 | C44 | A8C44 | [29] |
| Gorna Trape | Bu | 5 | KF888279 | KF888304 | A8 | C44 | A8C44 | [29] |
| Langsee | Ge | 1 | KF888279 | KF888298 | A8 | C45 | A8C45 | [29] |
| Farm Oeversee | Ge | 1 | KF888279 | KF888298 | A8 | C45 | A8C45 | [29] |
| Cladovita | Ro | 2 | KF888279 | KF888303 | A8 | C46 | A8C46 | [29] |
| Pét | Hu | 1 | KF888279 | KF888302 | A8 | C47 | A8C47 | [29] |
| Gorna Trape | Bu | 2 | KF888279 | KF888308 | A8 | C48 | A8C48 | [29] |
| Mühlgraben Caldern | Ge | 1 | KF888279 | KF888300 | A8 | C49 | A8C49 | [29] |
| Farm Augsburg | Ge | 3 | KF888279 | KF888300 | A8 | C49 | A8C49 | [29] |
| Libramont | Be | 2 | KF888282 | KF888296 | A8 | C17 | A8C17 | [29] |
| Nadas | Ro | 3 | KF888290 | KF888315 | A8 | C37 | A8C37 | [29] |
| Galben | Ro | 1 | KF888289 | KF888315 | A5 | C37 | A5C37 | [29] |
| Gut Rietberg | Ge | 1 | KF888281 | KF888315 | A7 | C37 | A7C37 | [29] |
| Klausbach | Ge | 4 | KF888281 | KF888315 | A7 | C37 | A7C37 | [29] |
| Pollichia Woog | Ge | 2 | KF888281 | KF888315 | A7 | C37 | A7C37 | [29] |
| Pollichia Woog2 | Ge | 2 | KF888281 | KF888315 | A7 | C37 | A7C37 | [29] |
| Schlettenbachtal | Ge | 1 | KF888281 | KF888315 | A7 | C37 | A7C37 | [29] |
| Wolfsägertal | Ge | 2 | KF888281 | KF888315 | A7 | C37 | A7C37 | [29] |
| Eifel | Ge | 1 | KF888281 | KF888315 | A7 | C37 | A7C37 | [29] |
| Spielberg | Ge | 3 | KF888281 | KF888315 | A7 | C37 | A7C37 | [29] |
| Farm Augsburg | Ge | 2 | KF888281 | KF888315 | A7 | C37 | A7C37 | [29] |
| Tebea | Ro | 1 | KF888291 | KF888315 | A12 | C37 | A12C37 | [29] |
| Sohodol | Ro | 1 | KF888291 | KF888315 | A12 | C37 | A12C37 | [29] |
| Racas | Ro | 1 | KF888291 | KF888315 | A12 | C37 | A12C37 | [29] |
| Barcau | Ro | 1 | KF888291 | KF888315 | A12 | C37 | A12C37 | [29] |
| Valea Stoiaca | Ro | 1 | KF888291 | KF888315 | A12 | C37 | A12C37 | [29] |
| Derjana | Ro | 1 | KF888291 | KF888315 | A12 | C37 | A12C37 | [29] |
| Balsa | Ro | 1 | KF888291 | KF888315 | A12 | C37 | A12C37 | [29] |
| Dragoiestilor | Ro | 5 | KF888291 | KF888315 | A12 | C37 | A12C37 | [29] |
| Balsa | Ro | 1 | KF888291 | KF888315 | A12 | C37 | A12C37 | [29] |
| Conop | Ro | 2 | KF888291 | KF888315 | A12 | C37 | A12C37 | [29] |
| Stramba | Ro | 1 | KF888291 | KF888315 | A12 | C37 | A12C37 | [29] |
| Valea Pestilor | Ro | 1 | KF888279 | KF888320 | A8 | C19 | A8C19 | [29] |
| Jaruga River, Stajničko polje | Cr | 2 | KF888288 | KF888315 | A8 | C37 | A8C37 | [29] |
| Plitvice Lakes - NP Plitvička jezera | Cr | 2 | KF888288 | KF888315 | A8 | C37 | A8C37 | [29] |
| Zeta River | Mo | 1 | KF888288 | KF888315 | A8 | C37 | A8C37 | [29] |
| Carpan | Ro | 1 | KF888279 | KF888318 | A8 | C38 | A8C38 | [29] |
| Fischbach | Ge | 3 | KF888279 | KF888317 | A8 | C6 | A8C6 | [29] |
| Mrežnica River, Generalski Stol | Cr | 2 | KF888288 | KF888314 | A8 | C7 | A8C7 | [29] |
| Dielbach (Woog) | Ge | 3 | KF888279 | KF888316 | A8 | C9 | A8C9 | [29] |
| Wolfsägertal | Ge | 2 | KF888279 | KF888316 | A8 | C9 | A8C9 | [29] |
| Saarbacher Mühlweiher | Ge | 2 | KF888279 | KF888316 | A8 | C9 | A8C9 | [29] |
| Saarbach/Saar-bachhammer | Ge | 2 | KF888279 | KF888316 | A8 | C9 | A8C9 | [29] |
| Saarbach (Woog) | Ge | 1 | KF888279 | KF888316 | A8 | C9 | A8C9 | [29] |
| Saarbacher Hammer | Ge | 1 | KF888279 | KF888316 | A8 | C9 | A8C9 | [29] |
| Zeta River | Mo | 1 | KF888288 | KF888316 | A8 | C9 | A8C9 | [29] |
| ? | ? | 1 | DQ320033 | AY667146 | A8 | C53 | A8C53 | [35] |
| Bacica Creek | Cr | 1 | KX370092 | KX369672 | A17 | C22 | A17C22 | [6] |

# References

6. Jelić M, Klobučar GIV, Grandjean F, Puillandre N, Franjević D, Futo M, et al. Insights into the molecular phylogeny and historical biogeography of the white-clawed crayfish (Decapoda, Astacidae). Mol. Phylogenet. Evol. 2016;103:26–40.

29. Schrimpf A, Theissinger K, Dahlem J, Maguire I, Pârvulescu L, Schulz HK, et al. Phylogeography of noble crayfish (*Astacus astacus*) reveals multiple refugia. Freshwater Biol. 2014;59:761–76.

35. Klobučar GI V, Podnar M, Jelić M, Franjević D, Faller M, Štambuk A, et al. Role of the Dinaric Karst (western Balkans) in shaping the phylogeographic structure of the threatened crayfish *Austropotamobius torrentium*. Freshwater Biol. 2013;58:1089–105.
