# Supplementary material for: Microevolution of the noble crayfish (Astacus astacus) in the Southern Balkan Peninsula
Source: BMC Evol Biol. 2017 May 30;17:122. doi: 10.1186/s12862-017-0971-6 (PMC5450353; doi:10.1186/s12862-017-0971-6)
Supplement: Supplementary file 2 — The modified conditions used for the PCR and amplification of each microsatellite loci are given. (DOC 32 kb) [file 12862_2017_971_MOESM2_ESM.doc]

# Additional file 2

Modified PCR and amplification conditions for each microsatellite loci used.

| **Microsatellite loci** | **MgCl_2_ (mM)** | **Primer concentration (pM)** | **Annealing Temperature (^o^C)** |
| --- | --- | --- | --- |
| Aas8 | 0 | 20 | 60 |
| Aas766 | 0 | 16.5 | 60 |
| Aas1198 | 0 | 16.5 | 60 |
| Aas2489 | 0 | 20 | 56 |
| Aas3040 | 0.5 | 20 | 56 |
| Aas3950 | 0 | 12.5 | 60 |
