# Supplementary material for: Microevolution of the noble crayfish (Astacus astacus) in the Southern Balkan Peninsula
Source: BMC Evol Biol. 2017 May 30;17:122. doi: 10.1186/s12862-017-0971-6 (PMC5450353; doi:10.1186/s12862-017-0971-6)
Supplement: Supplementary file 3 — Starting values (α, σ, β and τ) for hyperpriors (log(N0), log(N1), log(Θ) and log(T)) for each independent MCMC run. (DOC 30 kb) [file 12862_2017_971_MOESM3_ESM.doc]

# Additional file 3

Starting values (α, σ, β and τ) for hyperpriors (log(N0), log(N1), log(Θ) and log(T)) for each independent MCMC run.

|  | **Starting values (α, σ, β and τ) for hyperpriors** | | | |
| --- | --- | --- | --- | --- |
| **Runs** | **log(N0)** | **log(N1)** | **log(Θ)** | **log(T)** |
| 1 | 3 2 0 0.5 | 5 3 0 0.5 | -3.5 0.25 0 0.5 | 5 3 0 0.5 |
| 2 | 3 2 0 0.5 | 3 3 0 0.5 | -3.5 0.25 0 0.5 | 5 3 0 0.5 |
| 3 | 5 2 0 0.5 | 5 3 0 0.5 | -3.5 0.25 0 0.5 | 5 3 0 0.5 |
| 4 | 5 2 0 0.5 | 3 3 0 0.5 | -3.5 0.25 0 0.5 | 5 3 0 0.5 |
| 5 | 2 2 0 0.5 | 4 3 0 0.5 | -3.5 0.25 0 0.5 | 5 3 0 0.5 |
