# Supplementary material for: Microevolution of the noble crayfish (Astacus astacus) in the Southern Balkan Peninsula
Source: BMC Evol Biol. 2017 May 30;17:122. doi: 10.1186/s12862-017-0971-6 (PMC5450353; doi:10.1186/s12862-017-0971-6)
Supplement: Supplementary file 4 — Pairwise within and between genetic distances of the haplotype groups (G1 to G6) for COI and 16S. (DOC 56 kb) [file 12862_2017_971_MOESM4_ESM.doc]

# Additional file 4

Pairwise within and between genetic distances (Kimura two parameter - K2P and uncorrected - p) of the haplotype groups for 16S and COI (see Figure 1), using MEGA v. 6.06 [57].

| **Within mean group distances** | | | | |
| --- | --- | --- | --- | --- |
|  | **16S** | | **COI** | |
|  | **K2P** | **p** | **K2P** | **p** |
| G1 | 0.0010 | 0.0010 | 0.0350 | 0.0328 |
| G2 | 0.0020 | 0.0020 | 0.0108 | 0.0106 |
| G3 | 0.0102 | 0.0100 | 0.0066 | 0.0065 |
| G4 | 0.0013 | 0.0013 | 0.0046 | 0.0046 |
| G5 | 0.0016 | 0.0016 | 0.0066 | 0.0065 |
| G6 | 0.0013 | 0.0013 | 0.0037 | 0.0037 |

| **Between mean distances** | | | | | |
| --- | --- | --- | --- | --- | --- |
|  |  | **16S** | | **COI** | |
| **Phylogroups** | | **K2P** | **p** | **K2P** | **p** |
| G1 | G2 | 0.001 | 0.001 | 0.035 | 0.032 |
| G1 | G3 | 0.019 | 0.019 | 0.041 | 0.039 |
| G1 | G4 | 0.013 | 0.013 | 0.037 | 0.034 |
| G1 | G5 | 0.012 | 0.012 | 0.040 | 0.038 |
| G1 | G6 | 0.013 | 0.013 | 0.034 | 0.032 |
| G2 | G3 | 0.019 | 0.019 | 0.030 | 0.028 |
| G2 | G4 | 0.014 | 0.013 | 0.019 | 0.019 |
| G2 | G5 | 0.013 | 0.013 | 0.023 | 0.022 |
| G2 | G6 | 0.013 | 0.013 | 0.017 | 0.017 |
| G3 | G4 | 0.013 | 0.013 | 0.020 | 0.019 |
| G3 | G5 | 0.011 | 0.011 | 0.021 | 0.021 |
| G3 | G6 | 0.012 | 0.012 | 0.023 | 0.022 |
| G4 | G5 | 0.007 | 0.007 | 0.006 | 0.006 |
| G4 | G6 | 0.007 | 0.007 | 0.006 | 0.006 |
| G5 | G6 | 0.001 | 0.001 | 0.009 | 0.009 |

# References

57. Tamura K, Stecher G, Peterson D, Filipski A, Kumar S. MEGA6: Molecular evolutionary genetics analysis version 6.0. Mol. Biol. Evol. 2013;30:2725–9.
