# Supplementary material for: Microevolution of the noble crayfish (Astacus astacus) in the Southern Balkan Peninsula
Source: BMC Evol Biol. 2017 May 30;17:122. doi: 10.1186/s12862-017-0971-6 (PMC5450353; doi:10.1186/s12862-017-0971-6)
Supplement: Supplementary file 5 — Genetic diversity of the microsatellite loci and sampling sites. Table with the number of alleles, allelic richness, number of private alleles, expected heterozygosity, unbiased expected Nei’s heterozygosity, observed heterozygosity, inbreeding coefficient and exact P-value for Hardy-Weinberg equilibrium test are listed for the microsatellite loci and the sampling sites. (DOC 140 kb) [file 12862_2017_971_MOESM5_ESM.doc]

# Additional file 5

Genetic diversity at 6 microsatellite loci for 16 sites of the noble crayfish. Where: A, number of alleles; A_R_, Allelic richness; P_A_, number of private alleles; H_E_, expected heterozygosity; H_N_, unbiased expected Nei's heterozygosity; H_O_, observed heterozygosity; F_IS_, inbreeding coefficient; P_H-W_, exact P-value for Hardy-Weinberg equilibrium test; N/A, not available.

|  | **AA1** | **AA2** | **KAS** | **LOG** | **KNS** | **KLV** | **NEO** | **PLF** | **KRI** | **SKR** | **KRN** | **KPA** | **TZA** | **DOX** | **TSV** | **PRT** | **Average** |
| --- | --- | --- | --- | --- | --- | --- | --- | --- | --- | --- | --- | --- | --- | --- | --- | --- | --- |
| **Aas3950** |  |  |  |  |  |  |  |  |  |  |  |  |  |  |  |  |  |
| **A** | 5.000 | 6.000 | 12.000 | 1.000 | 2.000 | 6.000 | 3.000 | 9.000 | 3.000 | 2.000 | 1.000 | 3.000 | 8.000 | 8.000 | 13.000 | 9.000 | 21.000 |
| **A_R_** | 4.025 | 4.276 | 7.669 | 1.000 | 1.842 | 3.359 | 2.551 | 6.183 | 2.297 | 1.851 | 1.000 | 3.000 | 8.000 | 6.213 | 7.396 | 6.297 | 7.027 |
| **P_A_** | 0.000 | 0.000 | 0.000 | 0.000 | 0.000 | 0.000 | 0.000 | 0.000 | 1.000 | 0.000 | 0.000 | 0.000 | 0.000 | 0.000 | 2.000 | 0.000 | 3.000 |
| **H_E_** | 0.708 | 0.691 | 0.890 | 0.000 | 0.208 | 0.379 | 0.335 | 0.836 | 0.471 | 0.219 | 0.000 | 0.625 | 0.847 | 0.845 | 0.876 | 0.849 | 0.549 |
| **H_N_** | 0.726 | 0.709 | 0.913 | 0.000 | 0.214 | 0.391 | 0.353 | 0.858 | 0.483 | 0.224 | 0.000 | 0.682 | 0.924 | 0.864 | 0.899 | 0.869 | 0.569 |
| **H_O_** | 0.800 | 0.900 | 0.850 | 0.000 | 0.235 | 0.375 | 0.400 | 0.800 | 0.400 | 0.250 | 0.000 | 10.000 | 10.000 | 0.913 | 0.850 | 0.818 | 1.724 |
| **F_IS_** | -0.105 | -0.279 | 0.071 | N/A | -0.103 | 0.043 | -0.143 | 0.069 | 0.176 | -0.118 | N/A | -0.538 | -0.091 | -0.058 | 0.056 | 0.060 |  |
| **P_H-W_** | 0.804 | 0.353 | 0.687 | N/A | 1.000 | 0.498 | 1.000 | 0.101 | 0.739 | 1.000 | N/A | 0.091 | 1.000 | 0.820 | 0.391 | 0.793 | 0.941 |
| **Aas766** |  |  |  |  |  |  |  |  |  |  |  |  |  |  |  |  |  |
| **A** | 3.000 | 4.000 | 5.000 | 1.000 | 1.000 | 2.000 | 3.000 | 6.000 | 1.000 | 1.000 | 1.000 | 2.000 | 3.000 | 6.000 | 4.000 | 3.000 | 11.000 |
| **A_R_** | 2.184 | 3.145 | 4.045 | 1.000 | 1.000 | 1.617 | 2.091 | 4.535 | 1.000 | 1.000 | 1.000 | 2.000 | 3.000 | 4.662 | 3.625 | 2.734 | 4.159 |
| **P_A_** | 0.000 | 0.000 | 1.000 | 0.000 | 0.000 | 0.000 | 1.000 | 1.000 | 0.000 | 0.000 | 0.000 | 0.000 | 0.000 | 0.000 | 2.000 | 0.000 | 5.000 |
| **H_E_** | 0.226 | 0.591 | 0.654 | 0.000 | 0.000 | 0.117 | 0.169 | 0.744 | 0.000 | 0.000 | 0.000 | 0.486 | 0.403 | 0.756 | 0.671 | 0.569 | 0.337 |
| **H_N_** | 0.232 | 0.606 | 0.671 | 0.000 | 0.000 | 0.121 | 0.178 | 0.763 | 0.000 | 0.000 | 0.000 | 0.530 | 0.439 | 0.773 | 0.689 | 0.583 | 0.349 |
| **H_O_** | 0.150 | 0.550 | 0.850 | 0.000 | 0.000 | 0.125 | 0.182 | 0.700 | 0.000 | 0.000 | 0.000 | 0.500 | 0.500 | 0.913 | 0.550 | 0.500 | 0.345 |
| **F_IS_** | 0.360 | 0.095 | -0.277 | N/A | N/A | -0.034 | -0.026 | 0.084 | N/A | N/A | N/A | 0.063 | -0.154 | -0.186 | 0.205 | 0.144 |  |
| **P_H-W_** | 0.033 | 0.752 | 0.075 | N/A | N/A | 1.000 | 1.000 | 0.624 | N/A | N/A | N/A | 1.000 | 1.000 | 0.106 | 0.392 | 0.055 | 0.263 |
| **Aas2489** |  |  |  |  |  |  |  |  |  |  |  |  |  |  |  |  |  |
| **A** | 3.000 | 3.000 | 6.000 | 2.000 | 2.000 | 2.000 | 1.000 | 6.000 | 5.000 | 3.000 | 3.000 | 3.000 | 4.000 | 6.000 | 6.000 | 9.000 | 12.000 |
| **A_R_** | 2.959 | 2.927 | 4.737 | 1.991 | 1.982 | 1.617 | 1.000 | 4.743 | 2.631 | 2.810 | 2.076 | 3.000 | 4.000 | 3.985 | 4.804 | 5.321 | 5.589 |
| **P_A_** | 0.000 | 0.000 | 0.000 | 0.000 | 0.000 | 0.000 | 0.000 | 0.000 | 0.000 | 0.000 | 0.000 | 0.000 | 0.000 | 0.000 | 0.000 | 1.000 | 1.000 |
| **H_E_** | 0.611 | 0.591 | 0.738 | 0.389 | 0.360 | 0.117 | 0.000 | 0.766 | 0.271 | 0.489 | 0.224 | 0.569 | 0.694 | 0.546 | 0.755 | 0.781 | 0.494 |
| **H_N_** | 0.627 | 0.606 | 0.756 | 0.401 | 0.371 | 0.121 | 0.000 | 0.786 | 0.278 | 0.501 | 0.230 | 0.621 | 0.758 | 0.559 | 0.774 | 0.799 | 0.512 |
| **H_O_** | 0.400 | 0.800 | 0.700 | 0.529 | 0.235 | 0.125 | 0.000 | 0.850 | 0.300 | 0.550 | 0.250 | 0.667 | 0.833 | 0.522 | 0.750 | 0.864 | 0.523 |
| **F_IS_** | 0.368 | -0.330 | 0.076 | -0.333 | 0.373 | -0.034 | N/A | -0.084 | -0.081 | -0.100 | -0.092 | -0.081 | -0.111 | 0.067 | 0.032 | -0.083 |  |
| **P_H-W_** | 0.021 | 0.362 | 0.578 | 0.279 | 0.178 | 1.000 | N/A | 0.984 | 1.000 | 0.615 | 1.000 | 1.000 | 0.324 | 0.336 | 0.571 | 0.250 | 0.664 |
| **Aas3040** |  |  |  |  |  |  |  |  |  |  |  |  |  |  |  |  |  |
| **A** | 3.000 | 4.000 | 6.000 | 2.000 | 2.000 | 3.000 | 3.000 | 2.000 | 2.000 | 2.000 | 2.000 | 2.000 | 4.000 | 9.000 | 6.000 | 7.000 | 13.000 |
| **A_R_** | 2.627 | 3.034 | 3.720 | 1.588 | 1.353 | 2.374 | 2.805 | 1.995 | 2.000 | 1.999 | 1.515 | 2.000 | 4.000 | 5.457 | 4.275 | 4.789 | 6.003 |
| **P_A_** | 0.000 | 0.000 | 0.000 | 0.000 | 0.000 | 0.000 | 0.000 | 0.000 | 0.000 | 0.000 | 0.000 | 0.000 | 0.000 | 2.000 | 0.000 | 0.000 | 2.000 |
| **H_E_** | 0.374 | 0.414 | 0.538 | 0.111 | 0.057 | 0.490 | 0.574 | 0.420 | 0.495 | 0.469 | 0.095 | 0.153 | 0.694 | 0.803 | 0.711 | 0.745 | 0.446 |
| **H_N_** | 0.383 | 0.424 | 0.551 | 0.114 | 0.059 | 0.506 | 0.602 | 0.431 | 0.508 | 0.481 | 0.097 | 0.167 | 0.758 | 0.820 | 0.730 | 0.762 | 0.462 |
| **H_O_** | 0.300 | 0.450 | 0.550 | 0.118 | 0.059 | 0.625 | 0.636 | 0.300 | 0.500 | 0.350 | 0.100 | 0.167 | 0.667 | 0.913 | 0.650 | 0.727 | 0.444 |
| **F_IS_** | 0.222 | -0.062 | 0.002 | -0.032 | 0.000 | -0.245 | -0.061 | 0.309 | 0.016 | 0.277 | -0.027 | 0.000 | 0.130 | -0.116 | 0.112 | 0.047 |  |
| **P_H-W_** | 0.214 | 0.409 | 0.552 | 1.000 | N/A | 0.725 | 0.586 | 0.279 | 1.000 | 0.338 | 1.000 | N/A | 0.580 | 0.932 | 0.341 | 0.667 | 0.955 |
| **Aas1198** |  |  |  |  |  |  |  |  |  |  |  |  |  |  |  |  |  |
| **A** | 4.000 | 7.000 | 12.000 | 8.000 | 7.000 | 5.000 | 2.000 | 4.000 | 4.000 | 4.000 | 6.000 | 2.000 | 5.000 | 15.000 | 9.000 | 9.000 | 39.000 |
| **A_R_** | 2.600 | 4.401 | 6.875 | 5.237 | 5.235 | 2.990 | 1.600 | 2.990 | 3.199 | 3.676 | 4.546 | 2.000 | 5.000 | 7.598 | 5.795 | 6.189 | 7.370 |
| **P_A_** | 0.000 | 3.000 | 4.000 | 1.000 | 0.000 | 1.000 | 0.000 | 1.000 | 0.000 | 0.000 | 0.000 | 0.000 | 1.000 | 6.000 | 2.000 | 3.000 | 22.000 |
| **H_E_** | 0.546 | 0.691 | 0.845 | 0.723 | 0.789 | 0.371 | 0.095 | 0.465 | 0.621 | 0.685 | 0.728 | 0.375 | 0.528 | 0.868 | 0.794 | 0.835 | 0.622 |
| **H_N_** | 0.560 | 0.709 | 0.867 | 0.745 | 0.813 | 0.383 | 0.100 | 0.477 | 0.637 | 0.703 | 0.746 | 0.409 | 0.576 | 0.887 | 0.814 | 0.854 | 0.643 |
| **H_O_** | 0.400 | 0.750 | 0.900 | 0.941 | 0.765 | 0.250 | 0.100 | 0.300 | 0.650 | 0.650 | 0.800 | 0.167 | 0.500 | 0.870 | 0.900 | 0.682 | 0.602 |
| **F_IS_** | 0.291 | -0.059 | -0.040 | -0.274 | 0.061 | 0.355 | 0.000 | 0.377 | -0.021 | 0.077 | -0.074 | 0.615 | 0.143 | 0.020 | -0.109 | 0.206 |  |
| **P_H-W_** | 0.161 | 0.873 | 0.933 | 0.908 | 0.191 | 0.038 | N/A | 0.005 | 0.139 | 0.550 | 0.265 | 0.274 | 0.534 | 0.528 | 0.936 | 0.037 | 0.044 |
| **Aas8** |  |  |  |  |  |  |  |  |  |  |  |  |  |  |  |  |  |
| **A** | 4.000 | 2.000 | 8.000 | 1.000 | 1.000 | 7.000 | 5.000 | 5.000 | 1.000 | 1.000 | 1.000 | 2.000 | 5.000 | 5.000 | 6.000 | 7.000 | 15.000 |
| **A_R_** | 2.666 | 1.985 | 6.357 | 1.000 | 1.000 | 4.604 | 3.636 | 4.045 | 1.000 | 1.000 | 1.000 | 2.000 | 5.000 | 4.083 | 4.218 | 5.149 | 6.533 |
| **P_A_** | 0.000 | 0.000 | 0.000 | 0.000 | 0.000 | 0.000 | 0.000 | 1.000 | 0.000 | 0.000 | 0.000 | 0.000 | 0.000 | 0.000 | 2.000 | 0.000 | 3.000 |
| **H_E_** | 0.341 | 0.375 | 0.854 | 0.000 | 0.000 | 0.715 | 0.620 | 0.654 | 0.000 | 0.000 | 0.000 | 0.278 | 0.681 | 0.733 | 0.601 | 0.786 | 0.415 |
| **H_N_** | 0.350 | 0.385 | 0.876 | 0.000 | 0.000 | 0.738 | 0.649 | 0.671 | 0.000 | 0.000 | 0.000 | 0.303 | 0.742 | 0.749 | 0.617 | 0.804 | 0.430 |
| **H_O_** | 0.400 | 0.100 | 0.750 | 0.000 | 0.000 | 0.500 | 0.818 | 0.600 | 0.000 | 0.000 | 0.000 | 0.333 | 0.500 | 0.478 | 0.600 | 0.773 | 0.366 |
| **F_IS_** | -0.147 | 0.745 | 0.147 | N/A | N/A | 0.330 | -0.277 | 0.108 | N/A | N/A | N/A | -0.111 | 0.348 | 0.366 | 0.028 | 0.040 |  |
| **P_H-W_** | 1.000 | 0.003 | 0.123 | N/A | N/A | 0.019 | 0.408 | 0.289 | N/A | N/A | N/A | 1.000 | 0.165 | 0.003 | 0.745 | 0.558 | 0.003 |
