# Supplementary material for: Microevolution of the noble crayfish (Astacus astacus) in the Southern Balkan Peninsula
Source: BMC Evol Biol. 2017 May 30;17:122. doi: 10.1186/s12862-017-0971-6 (PMC5450353; doi:10.1186/s12862-017-0971-6)
Supplement: Supplementary file 6 — Fixation indices for all microsatellite loci. Values of the fixation indices for all microsatellite loci based on infinite allele model (FIT, FIS and FST) and stepwise mutation model (RST). (DOC 30 kb) [file 12862_2017_971_MOESM6_ESM.doc]

# Additional file 6

**Fixation indices for all microsatellite loci based on infinite alleles model (F_IT_, F_IS_ and F_ST_) and stepwise mutation model (R_ST_).**

| **Locus** | **F_IT_** | **F_IS_** | **F_ST_** | **R_ST_** |
| --- | --- | --- | --- | --- |
| Aas3950 | 0.34 | -0.02 | 0.36 | 0.26 |
| Aas766 | 0.50 | 0.02 | 0.49 | 0.47 |
| Aas2489 | 0.35 | -0.01 | 0.36 | 0.27 |
| Aas3040 | 0.48 | 0.04 | 0.46 | 0.52 |
| Aas1198 | 0.26 | 0.05 | 0.23 | 0.37 |
| Aas8 | 0.60 | 0.17 | 0.52 | 0.44 |
| Overall | 0.42 | 0.04 | 0.40 | 0.39 |
