# Supplementary material for: Microevolution of the noble crayfish (Astacus astacus) in the Southern Balkan Peninsula
Source: BMC Evol Biol. 2017 May 30;17:122. doi: 10.1186/s12862-017-0971-6 (PMC5450353; doi:10.1186/s12862-017-0971-6)
Supplement: Supplementary file 7 — Estimated pairwise FST and RST values based on sampling sites. (DOC 53 kb) [file 12862_2017_971_MOESM7_ESM.doc]

# Additional file 7

Estimated F_ST_ (above diagonal) and R_ST_ (below diagonal) values for each site pair of *Astacus astacus*. Underline values indicate neighboring sites from the same river system and bold values, site with known human-mediated intervention.

|  | **AA1** | **AA2** | **KAS** | **LOG** | **KNS** | **KLV** | **NEO** | **PLF** | **KRI** | **SKR** | **KRN** | **KPA** | **TZA** | **DOX** | **TSV** | **PRT** |
| --- | --- | --- | --- | --- | --- | --- | --- | --- | --- | --- | --- | --- | --- | --- | --- | --- |
| **AA1** |  | 0.0159 | 0.1388 | 0.6163 | 0.5979 | 0.4249 | 0.4432 | 0.3121 | 0.4690 | 0.4794 | 0.6483 | 0.0368 | 0.1901 | 0.2892 | 0.2957 | 0.2911 |
| **AA2** | 0.0543 |  | 0.1023 | 0.5509 | 0.5283 | 0.3984 | 0.4104 | 0.2548 | 0.4511 | 0.4616 | 0.5801 | 0.0218 | 0.1562 | 0.2455 | 0.2444 | 0.2504 |
| **KAS** | 0.0617 | 0.0996 |  | 0.4563 | 0.4357 | 0.2779 | 0.2932 | 0.1323 | 0.3324 | 0.3444 | 0.4835 | 0.1289 | 0.0256 | 0.1327 | 0.1216 | 0.1092 |
| **LOG** | 0.6086 | 0.5252 | 0.3580 |  | **0.0290** | 0.6061 | 0.6541 | 0.5234 | 0.7319 | 0.7320 | **0.1193** | 0.7063 | 0.6158 | 0.4589 | 0.4906 | 0.4481 |
| **KNS** | 0.6227 | 0.5452 | 0.3771 | **-0.0104** |  | 0.5708 | 0.6104 | 0.4912 | 0.7160 | 0.7158 | **0.0952** | 0.6710 | 0.5742 | 0.4407 | 0.4741 | 0.4295 |
| **KLV** | 0.1205 | 0.2373 | 0.0556 | 0.6589 | 0.6789 |  | -0.0069 | 0.3632 | 0.5450 | 0.5442 | 0.6138 | 0.4840 | 0.3194 | 0.3145 | 0.3327 | 0.2966 |
| **NEO** | 0.1537 | 0.2767 | 0.0579 | 0.6789 | 0.6982 | -0.0325 |  | 0.3724 | 0.5933 | 0.5934 | 0.6636 | 0.5155 | 0.3525 | 0.3324 | 0.3548 | 0.3163 |
| **PLF** | 0.5552 | 0.5352 | 0.2578 | 0.4424 | 0.4779 | 0.5083 | 0.5156 |  | 0.4971 | 0.4966 | 0.5387 | 0.2611 | 0.1639 | 0.2077 | 0.2296 | 0.1876 |
| **KRI** | 0.6348 | 0.5841 | 0.2413 | 0.8531 | 0.8664 | 0.6796 | 0.6981 | 0.6776 |  | 0.0203 | 0.7498 | 0.5649 | 0.3886 | 0.4104 | 0.3859 | 0.3940 |
| **SKR** | 0.5963 | 0.5496 | 0.2156 | 0.8283 | 0.8429 | 0.6364 | 0.6557 | 0.6338 | 0.0137 |  | 0.7499 | 0.5720 | 0.3962 | 0.4097 | 0.3961 | 0.3905 |
| **KRN** | 0.6368 | 0.5439 | 0.3686 | **0.0147** | **0.0442** | 0.7012 | 0.7225 | 0.5186 | 0.8778 | 0.8542 |  | 0.7353 | 0.6454 | 0.4833 | 0.5212 | 0.4719 |
| **KPA** | 0.0670 | 0.0451 | 0.1563 | 0.6641 | 0.6748 | 0.3674 | 0.4106 | 0.6566 | 0.7746 | 0.7417 | 0.6982 |  | 0.1823 | 0.2768 | 0.2907 | 0.2734 |
| **TZA** | 0.0393 | 0.0301 | -0.0208 | 0.4132 | 0.4385 | 0.0890 | 0.1104 | 0.3233 | 0.2768 | 0.2348 | 0.4323 | 0.1465 |  | 0.1459 | 0.1573 | 0.1314 |
| **DOX** | 0.1335 | 0.1476 | 0.0959 | 0.4047 | 0.4157 | 0.1852 | 0.2072 | 0.3072 | 0.4023 | 0.3486 | 0.4388 | 0.2223 | 0.0475 |  | 0.1320 | 0.0898 |
| **TSV** | 0.2474 | 0.2410 | 0.0609 | 0.4349 | 0.4572 | 0.2552 | 0.2655 | 0.2700 | 0.1863 | 0.1514 | 0.4555 | 0.3529 | 0.0424 | 0.1311 |  | 0.1032 |
| **PRT** | 0.2404 | 0.2692 | 0.0363 | 0.4964 | 0.5182 | 0.2199 | 0.2316 | 0.2534 | 0.3508 | 0.2885 | 0.5327 | 0.3951 | 0.0283 | 0.0744 | 0.0274 |  |
