# Supplementary material for: Microevolution of the noble crayfish (Astacus astacus) in the Southern Balkan Peninsula
Source: BMC Evol Biol. 2017 May 30;17:122. doi: 10.1186/s12862-017-0971-6 (PMC5450353; doi:10.1186/s12862-017-0971-6)
Supplement: Supplementary file 8 — Inference of number of clusters. The file contains graphical representations of a) DeltaK for each K (1 to 16) produced by Structure Harvester and b) Bayesian information criterion (BIC) for every number of clusters, using DAPC. (DOC 108 kb) [file 12862_2017_971_MOESM8_ESM.doc]

# Additional file 8

Inference of number of clusters: **A**) DeltaK for each K (1 to 16) produced by Structure Harvester [70], and **B**) Bayesian information criterion (BIC) for every number of clusters, using DAPC [73] in R package Adegenet v. 1.4-2 [74].


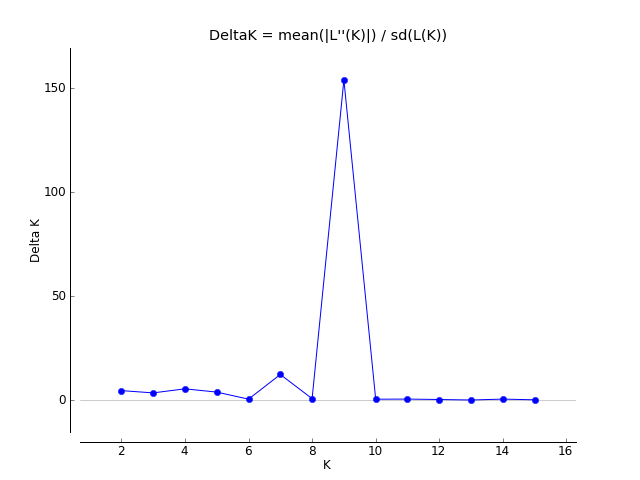


A)

**
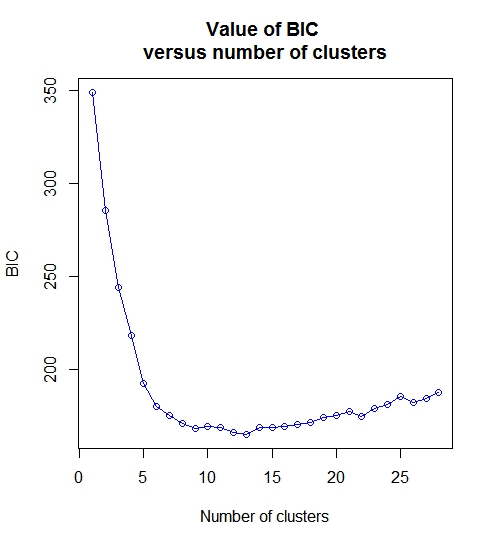
**

**Β)**

# References

(**A**)

(**b**)

(**B**)

70. Earl DA, VonHoldt BM. STRUCTURE HARVESTER: a website and program for visualizing STRUCTURE output and implementing the Evanno method. Conserv. Genet. Resour. Springer Netherlands; 2012;4:359–61.

73. Jombart T, Devillard S, Balloux F. Discriminant analysis of principal components: a new method for the analysis of genetically structured populations. BMC Genet. 2010;11:94.

74. Jombart T. Adegenet: a R package for the multivariate analysis of genetic markers. Bioinformatics. 2008;24:1403–5.
