# Supplementary material for: Microevolution of the noble crayfish (Astacus astacus) in the Southern Balkan Peninsula
Source: BMC Evol Biol. 2017 May 30;17:122. doi: 10.1186/s12862-017-0971-6 (PMC5450353; doi:10.1186/s12862-017-0971-6)
Supplement: Supplementary file 9 — Assignment probability (K between 6 and 9) and assignment probability per cluster, as identified by DAPC analysis. (DOC 35 kb) [file 12862_2017_971_MOESM9_ESM.doc]

# Additional file 9

Assignment probability (K between 6 and 9) and assignment probability per cluster, as identified by DAPC analysis (see Figure 4b).

| **K** | **Assignment probability** | **Assignment probability per cluster** | | | | | | | | |
| --- | --- | --- | --- | --- | --- | --- | --- | --- | --- | --- |
| **6** |  | **1** | **2** | **3** | **4** | **5** | **6** |  |  |  |
|  | 0.982 | 0.976 | 1.0 | 0.986 | 0.978 | 0.952 | 1.0 |  |  |  |
| **7** |  | **1** | **2** | **3** | **4** | **5** | **6** | **7** |  |  |
|  | 0.986 | 0.943 | 1.0 | 1.0 | 1.0 | 0.941 | 1.0 | 1.0 |  |  |
| **8** |  | **1** | **2** | **3** | **4** | **5** | **6** | **7** | **8** |  |
|  | 0.978 | 1.0 | 1.0 | 1.0 | 0.967 | 0.939 | 0.937 | 1.0 | 0.950 |  |
| **9** |  | **1** | **2** | **3** | **4** | **5** | **6** | **7** | **8** | **9** |
|  | 0.989 | 1.0 | 0.941 | 1.0 | 1.0 | 0.952 | 1.0 | 1.0 | 0.968 | 1.0 |
