# Supplementary material for: Microevolution of the noble crayfish (Astacus astacus) in the Southern Balkan Peninsula
Source: BMC Evol Biol. 2017 May 30;17:122. doi: 10.1186/s12862-017-0971-6 (PMC5450353; doi:10.1186/s12862-017-0971-6)
Supplement: Supplementary file 10 — Graphical representations of the DAPC scatterplots of the first two principal components for K between 6 and 8. (DOC 325 kb) [file 12862_2017_971_MOESM10_ESM.doc]

# Additional file 10

DAPC scatterplot of the first two principal components for K: (**A**) 6, (**B**) 7 and (**C**) 8. Clusters are represented by several distinguishable colors (same as in structure analysis, Figure 4b). The inset shows the discriminant analysis (DA) eigenvalues.

| 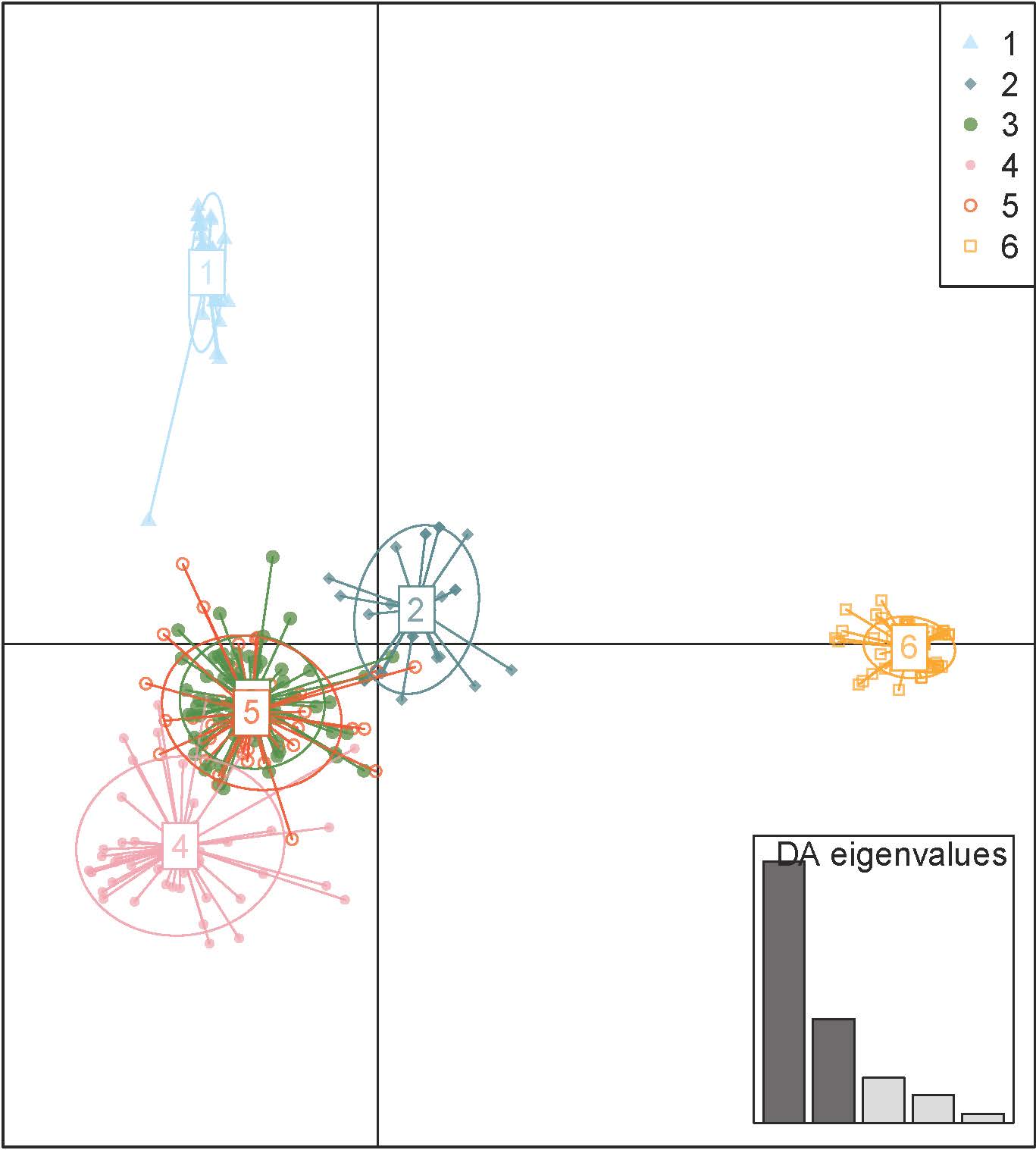  (**A**) | 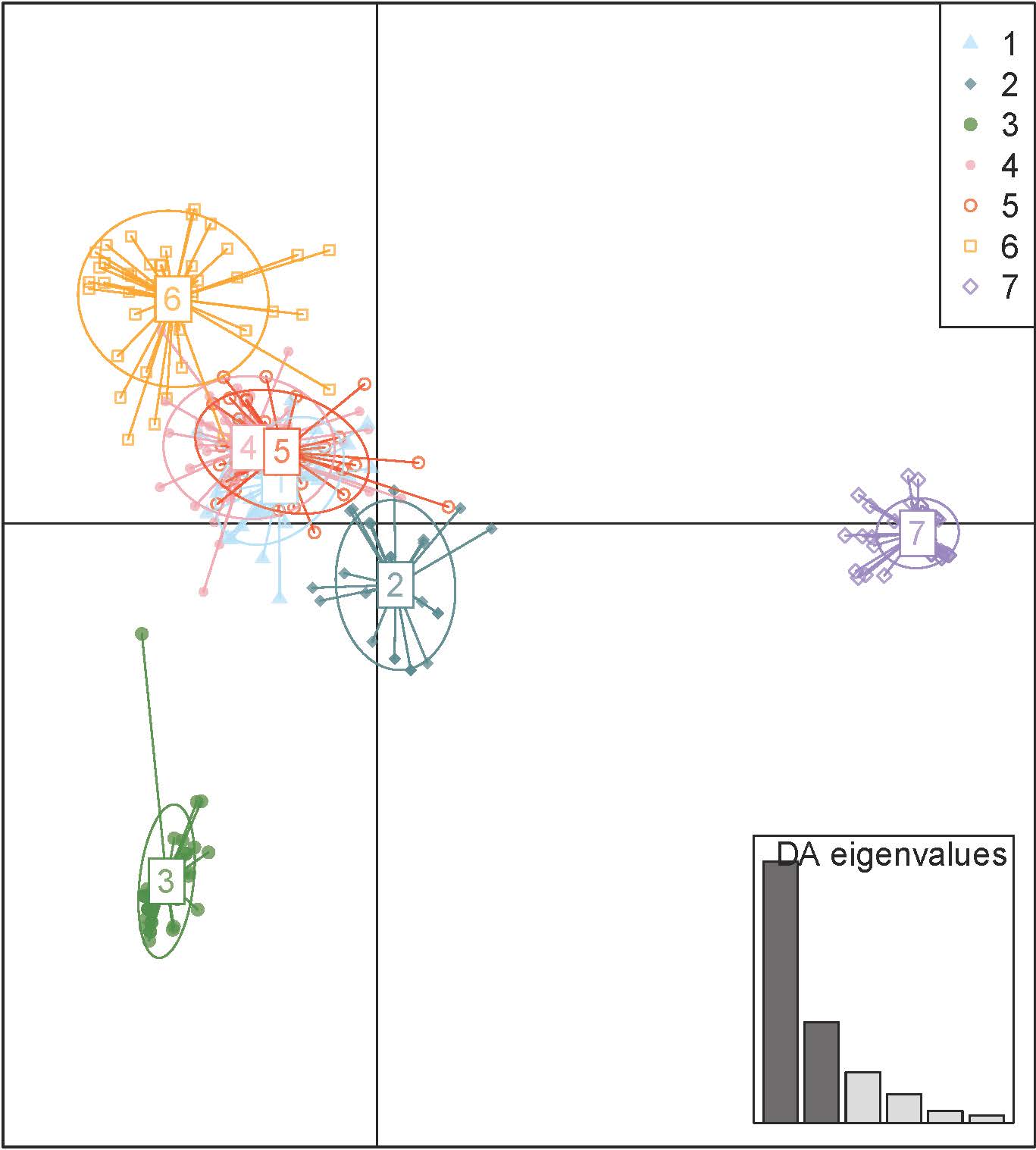  (**B**) |
| --- | --- |
| 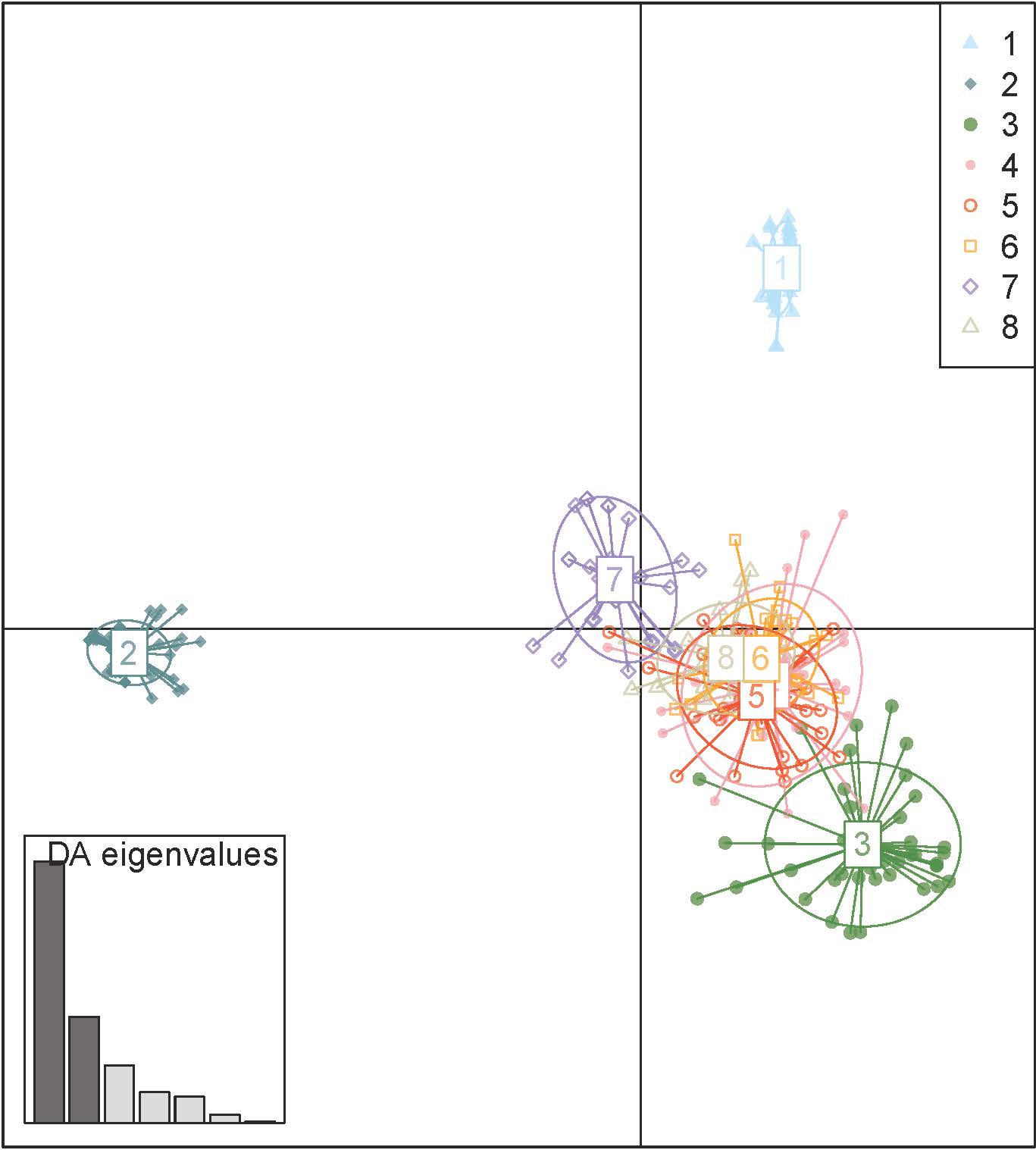  (**C**) |  |
