# Supplementary material for: Microevolution of the noble crayfish (Astacus astacus) in the Southern Balkan Peninsula
Source: BMC Evol Biol. 2017 May 30;17:122. doi: 10.1186/s12862-017-0971-6 (PMC5450353; doi:10.1186/s12862-017-0971-6)
Supplement: Supplementary file 11 — Mantel test correlation between geographical distance (kilometers) and genetic distances (given as FST, FST/(1-FST) or RST) for all sites. (DOC 637 kb) [file 12862_2017_971_MOESM11_ESM.doc]

**Additional file 11**

Mantel’s test correlation between geographical distance (kilometers) and genetic distances (given as F_ST_, F_ST_/(1-F_ST_) or R_ST_) for all sites.

**
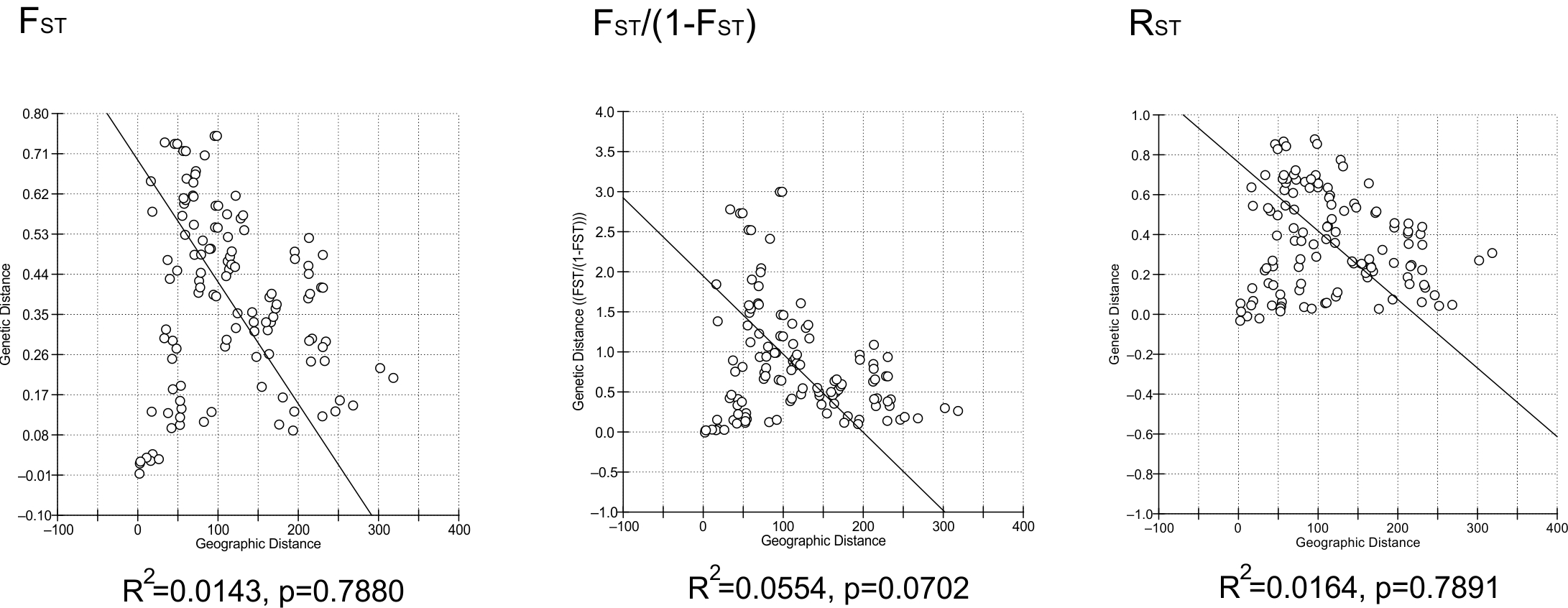
**
