# Supplementary material for: Microevolution of the noble crayfish (Astacus astacus) in the Southern Balkan Peninsula
Source: BMC Evol Biol. 2017 May 30;17:122. doi: 10.1186/s12862-017-0971-6 (PMC5450353; doi:10.1186/s12862-017-0971-6)

# Additional file 12

**A)** Graphical representation of genetic barriers (1 to 10) based on the sixteen sampling stations, all microsatellites loci and the genetic distance D_CE_. Genetic Barriers were created via the Delaunay triangulation (green lines) and Voronoi tessellation (blue polygons), as predicted by Barrier software. Red lines constitute the genetic barriers detected through the bootstrap analysis (10000 bootstraps) of D_CE_. The thickness of each genetic Barrier is proportional to the bootstrap support (green numbers).


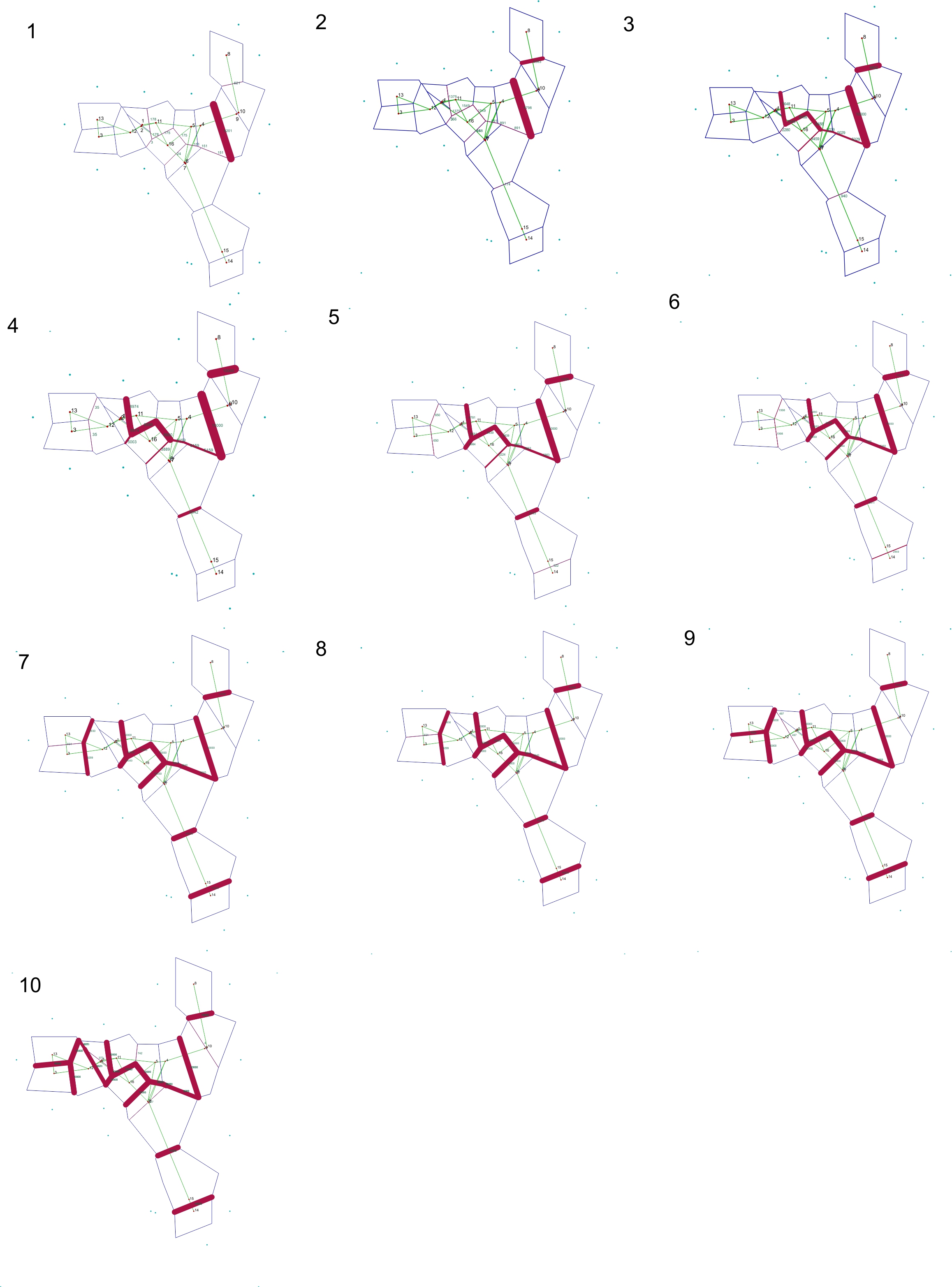


**B)** Graphical representation of genetic barriers (1 to 10) based on the sixteen sampling stations, all microsatellites loci and the F_ST_. Genetic barriers were created via the Delaunay triangulation (green lines) and Voronoi tessellation (blue polygons), as predicted by Barrier software. Red lines constitute the genetic Barriers detected through the analysis of the pairwise F_ST_ matrix for all microsatellite loci.


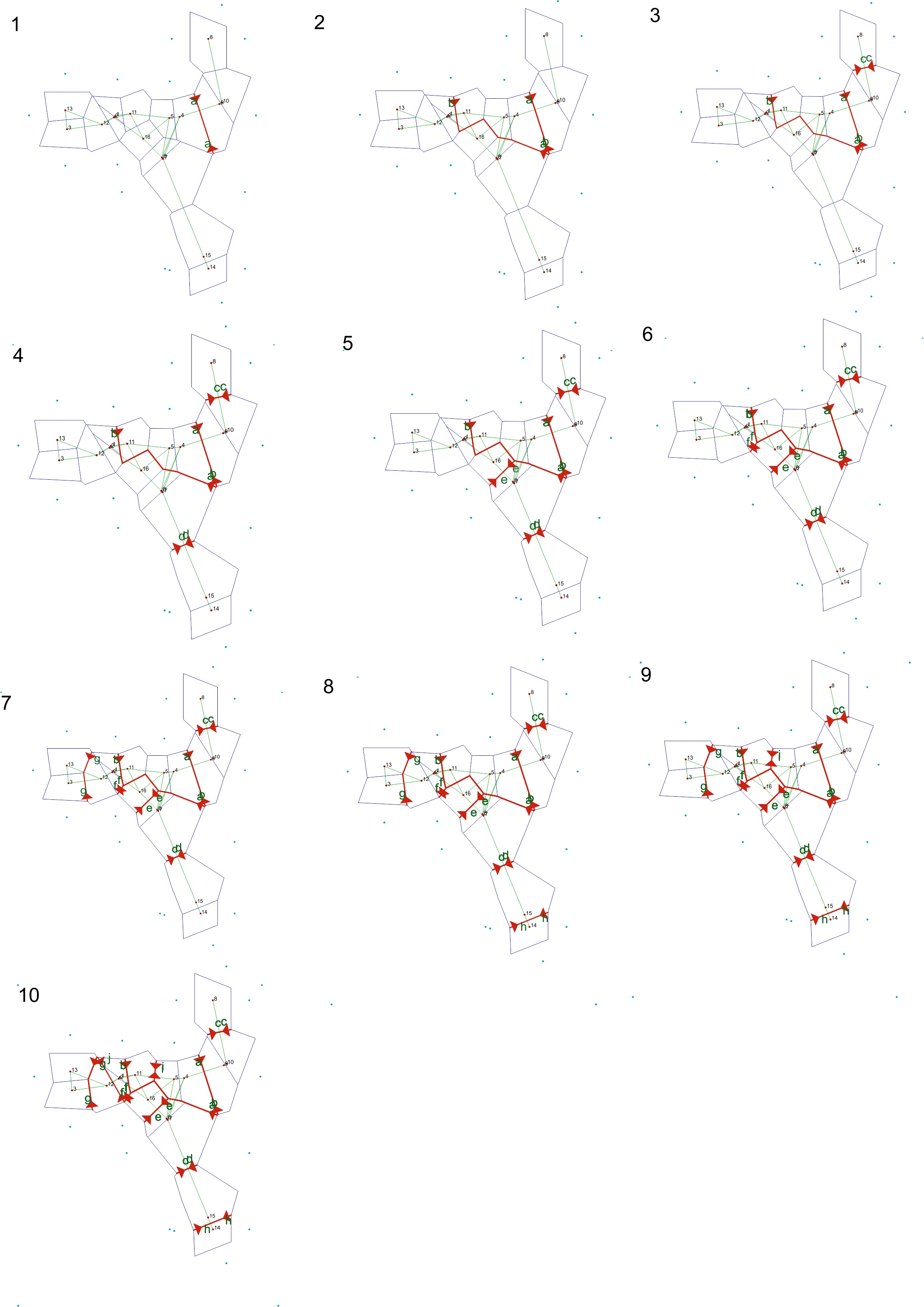


**C)** Graphical representation of genetic barriers (1 to 10) based on the sixteen sampling stations and F_ST_. The analysis was performed for every microsatellite locus separately (Aas8, Aas766, Aas1198, Aas2498, Aas3040 and Aas3950). Genetic barriers were created via the Delaunay triangulation (green lines) and Voronoi tessellation (blue polygons), as predicted by Barrier software. Red lines constitute the genetic Barriers detected through the analysis of the pairwise F_ST_ matrix for all microsatellite loci.


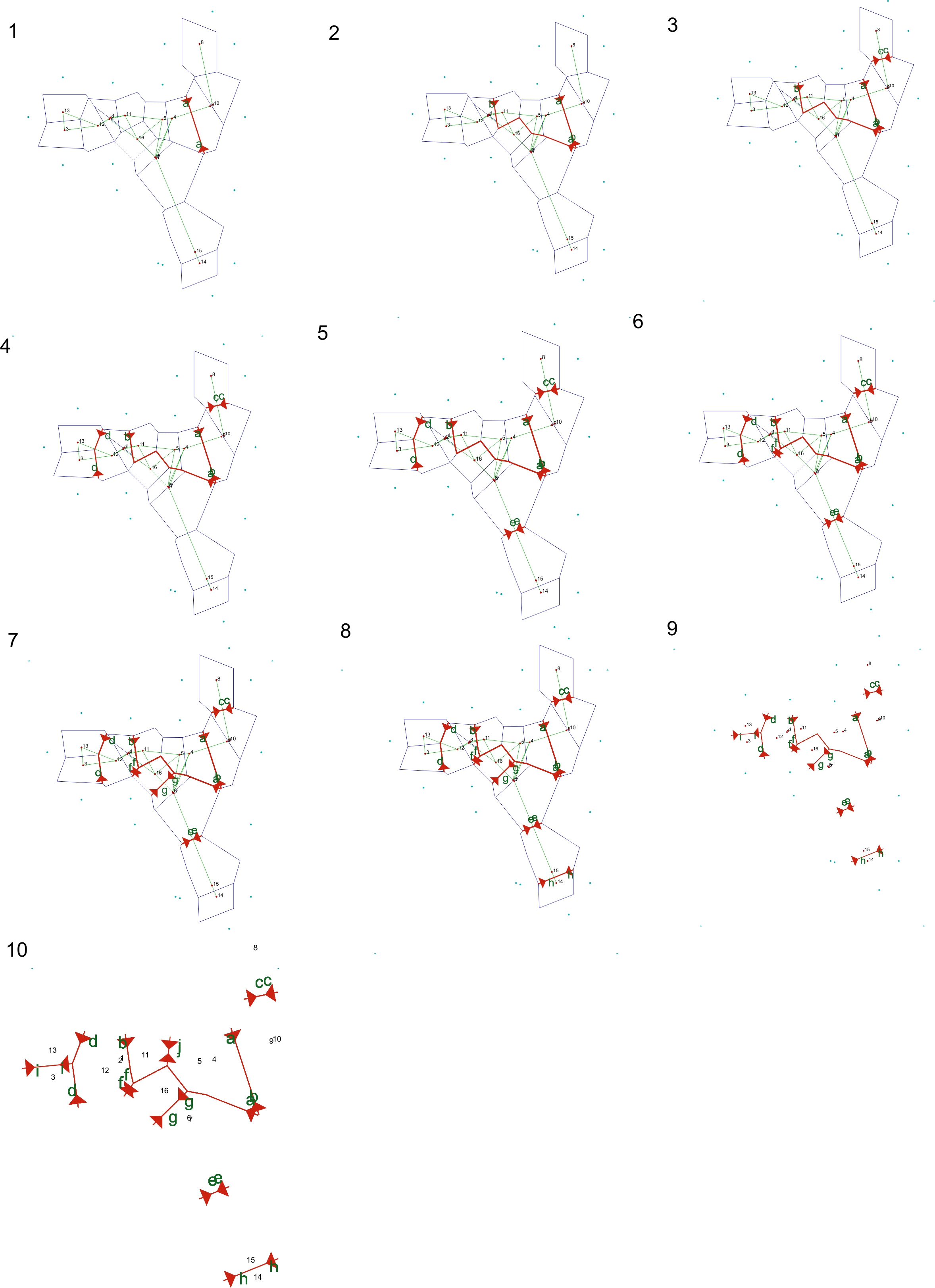


**Aas8**


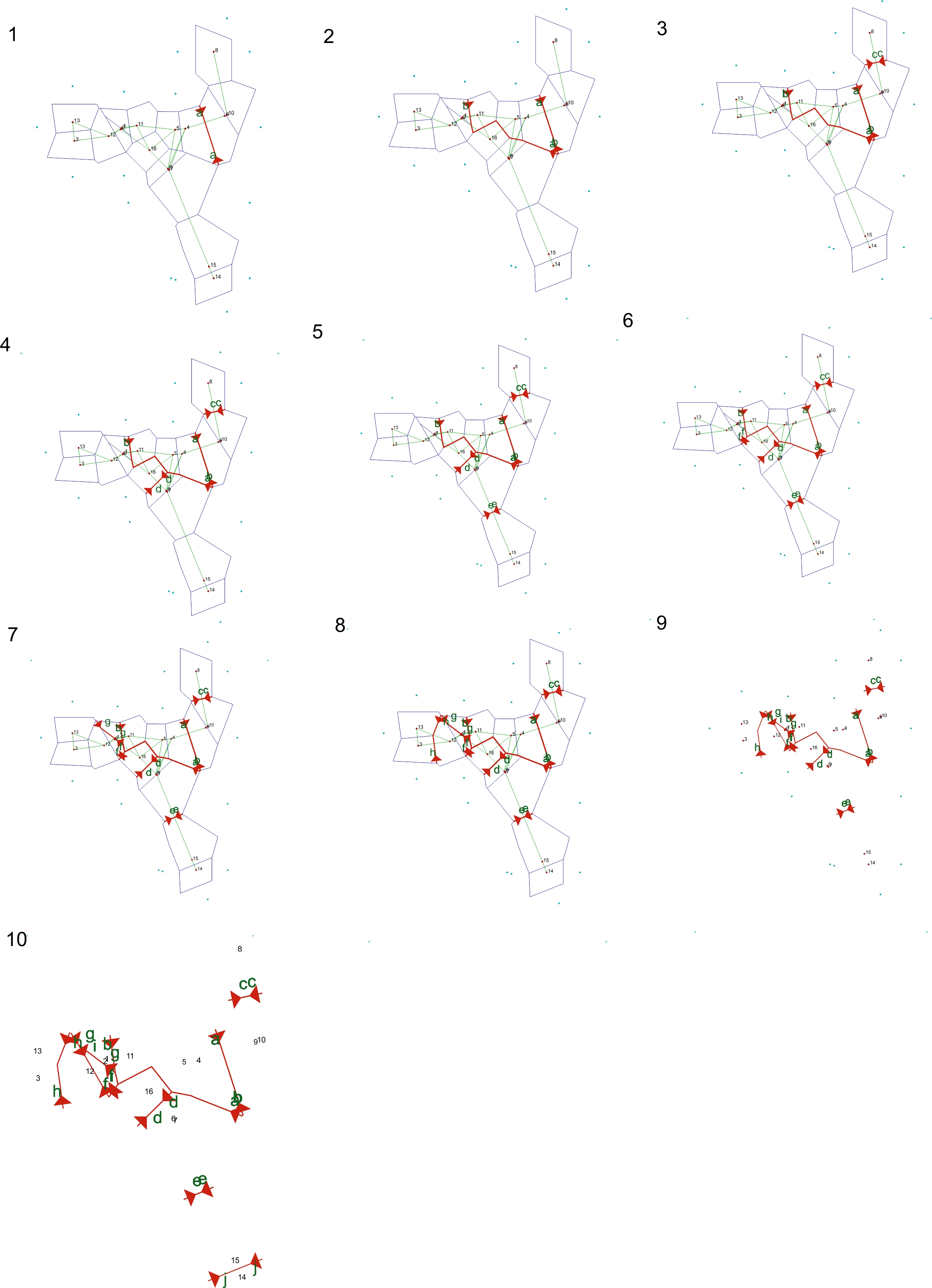


**Aas766**


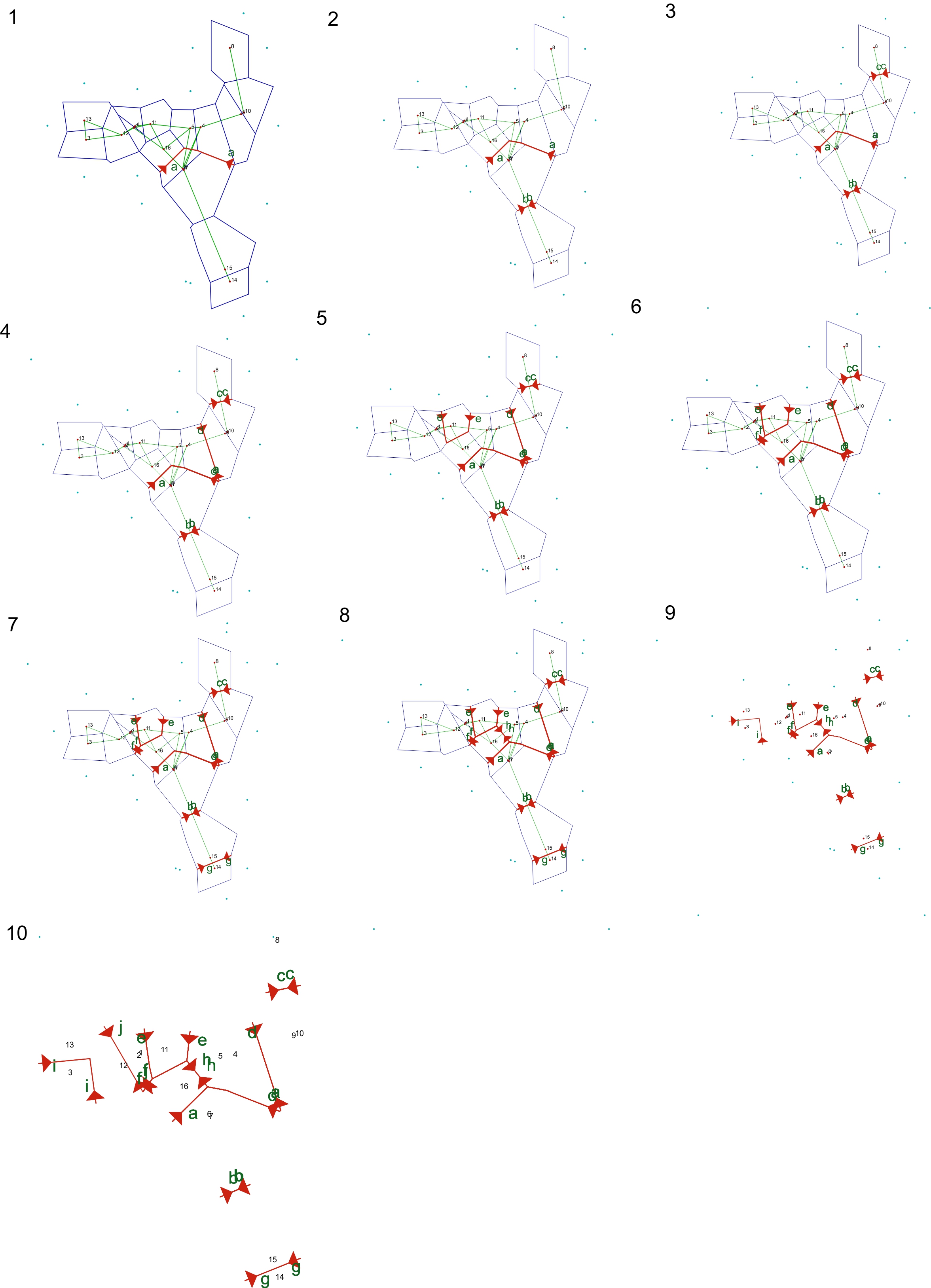


**Aas1198**


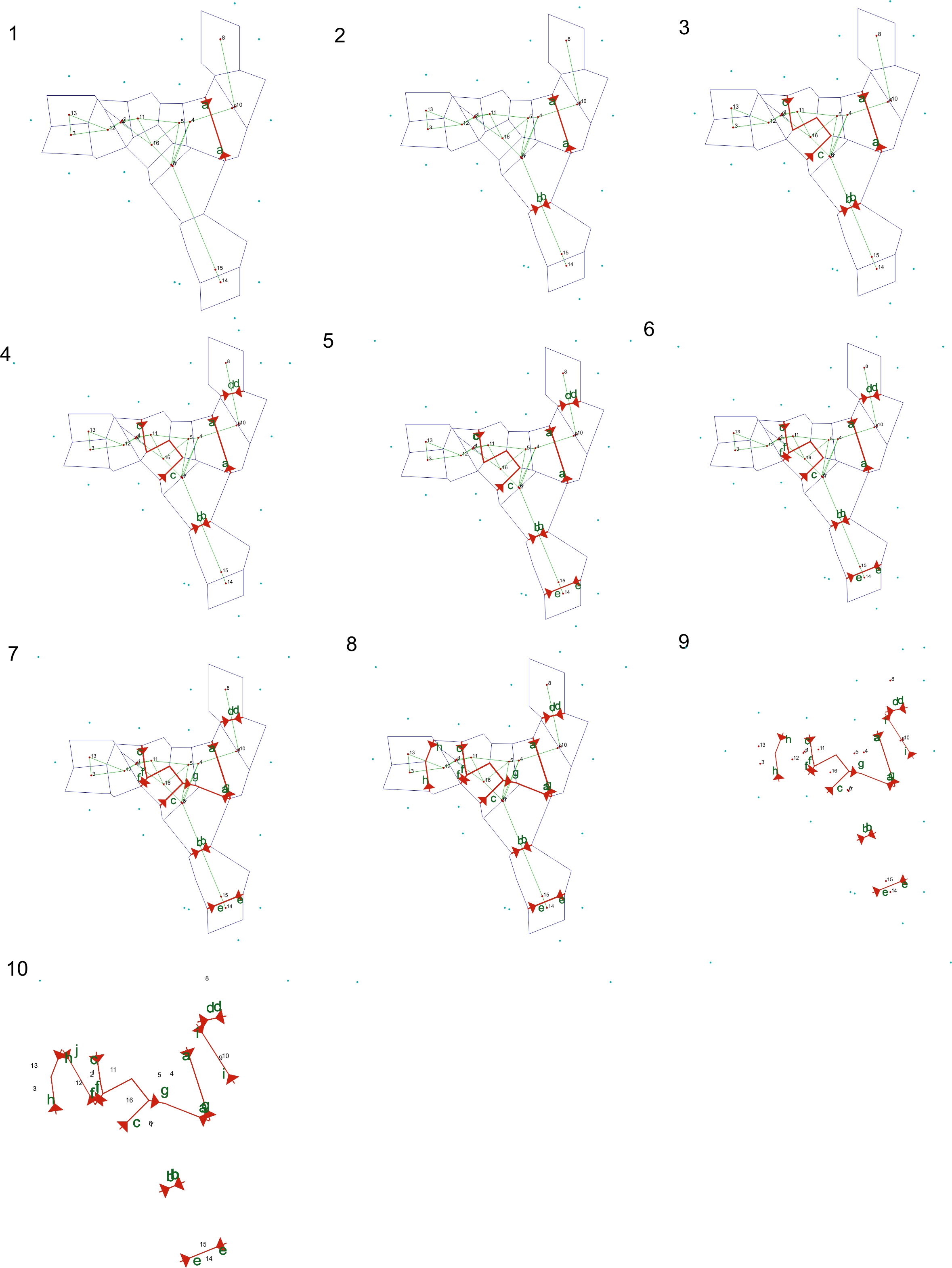


**Aas2498**


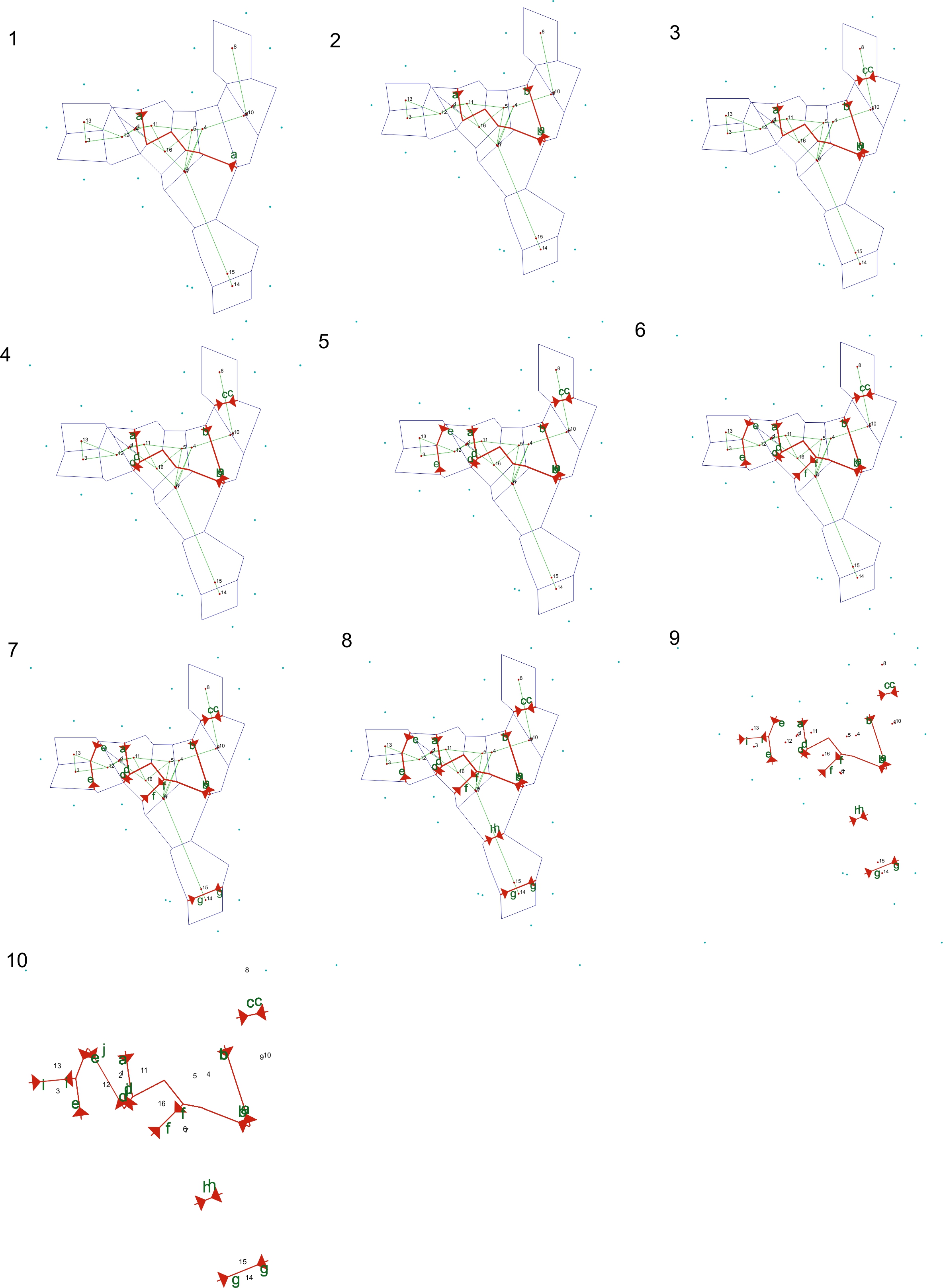


**Aas3040**


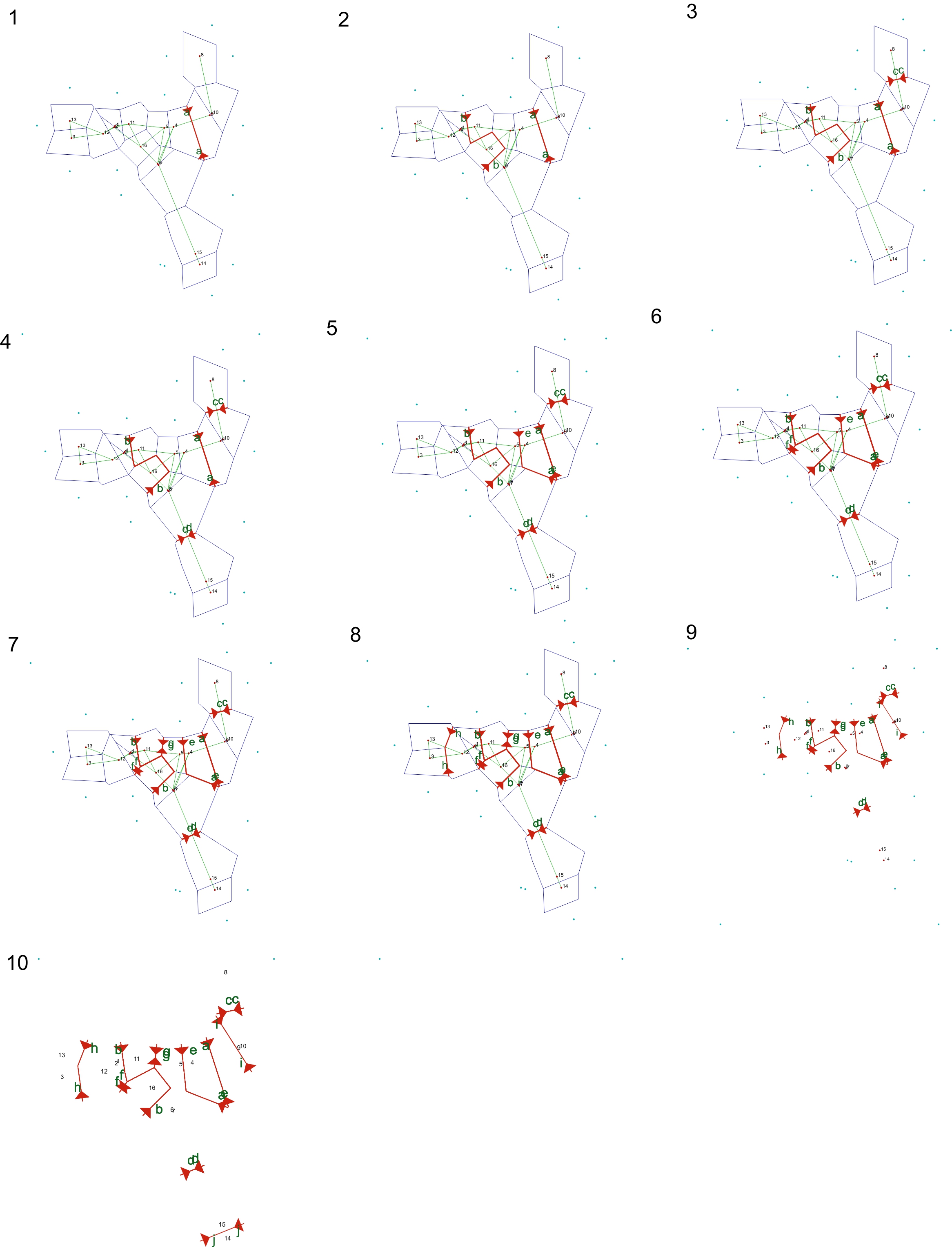


**Aas3950**

**D)** Graphical representation of genetic barriers (1 to 10) based on the nine genetic clusters (of the Structure software), all microsatellites loci and the genetic distance D_CE_. Genetic barriers were created via the Delaunay triangulation (green lines) and Voronoi tessellation (blue polygons), as predicted by Barrier software. Red lines constitute the genetic barriers detected through the bootstrap analysis (10000 bootstraps) of D_CE_. The thickness of each genetic barrier is proportional to the bootstrap support (green numbers).


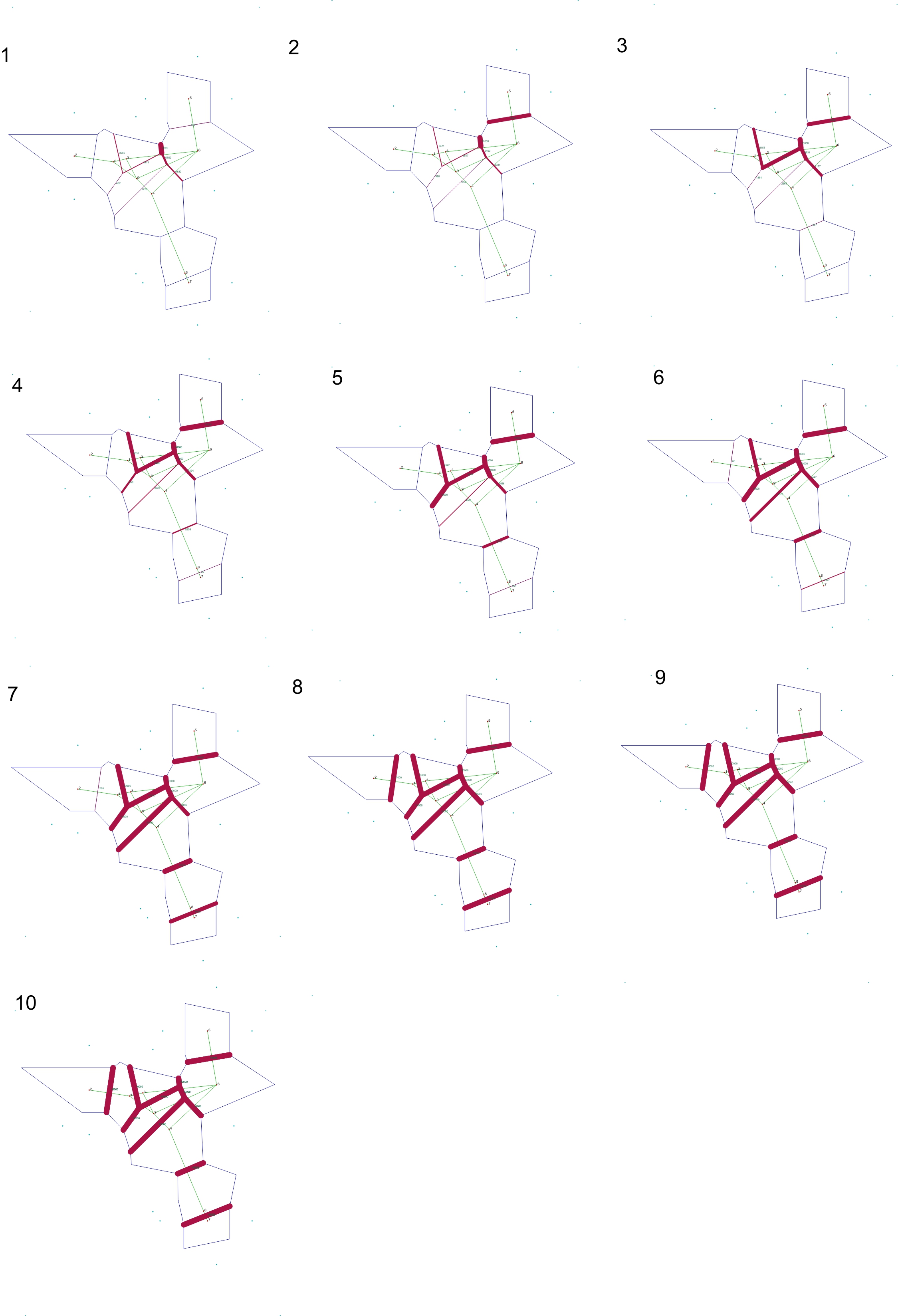


**E)** Graphical representation of genetic barriers (1 to 10) based on the nine genetic clusters (of the Structure software), all microsatellites loci and the F_ST_. Genetic barriers were created via the Delaunay triangulation (green lines) and Voronoi tessellation (blue polygons), as predicted by Barrier software. Red lines constitute the genetic barriers detected through the analysis of the pairwise F_ST_ matrix for all microsatellite loci.


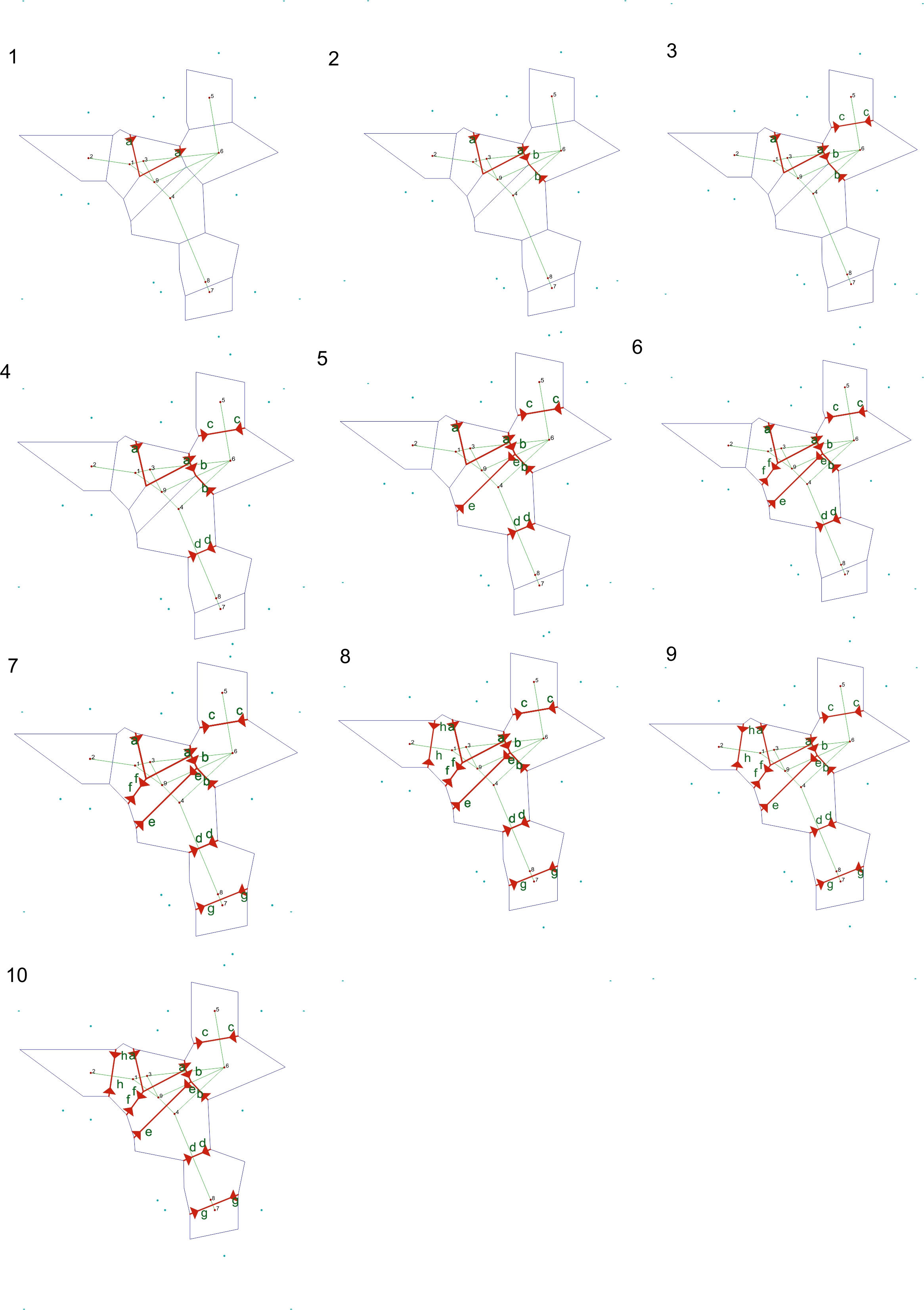

Supplement: Supplementary file 12 — Graphical representation of genetic barriers (1 to 10) based on: a) sixteen sampling stations, all microsatellites loci and the genetic distance DCE, b) sixteen sampling stations, all microsatellites loci and the FST, c) sixteen sampling stations, each microsatellite locus (Aas8, Aas766, Aas1198, Aas2498, Aas3040 and Aas3950) and FST, d) nine genetic clusters (of the Structure software), all microsatellites loci and the genetic distance DCE, and e) nine genetic clusters (of the Structure software), all microsatellites loci and the FST. (DOC 10235 kb) [file 12862_2017_971_MOESM12_ESM.doc]
