# Supplementary material for: Microevolution of the noble crayfish (Astacus astacus) in the Southern Balkan Peninsula
Source: BMC Evol Biol. 2017 May 30;17:122. doi: 10.1186/s12862-017-0971-6 (PMC5450353; doi:10.1186/s12862-017-0971-6)

# Additional file 13

Map of the sampled noble crayfish in Greece, created using ArcGIS® and ArcMap™ by Esri (Copyright © 2014 Esri and its licensors. All rights reserved). Abbreviation of the sampling sites (black letters, see also Table 1), Names of major rivers (blue letters), mountains (brown letters) and sierra (green letters), type of genetic markers used (mitochondrial and/or nuclear; mtDNA and nDNA, respectively) (blue or red circles), and elevation (divided in four groups: green, yellow, brownish and brown) are given.


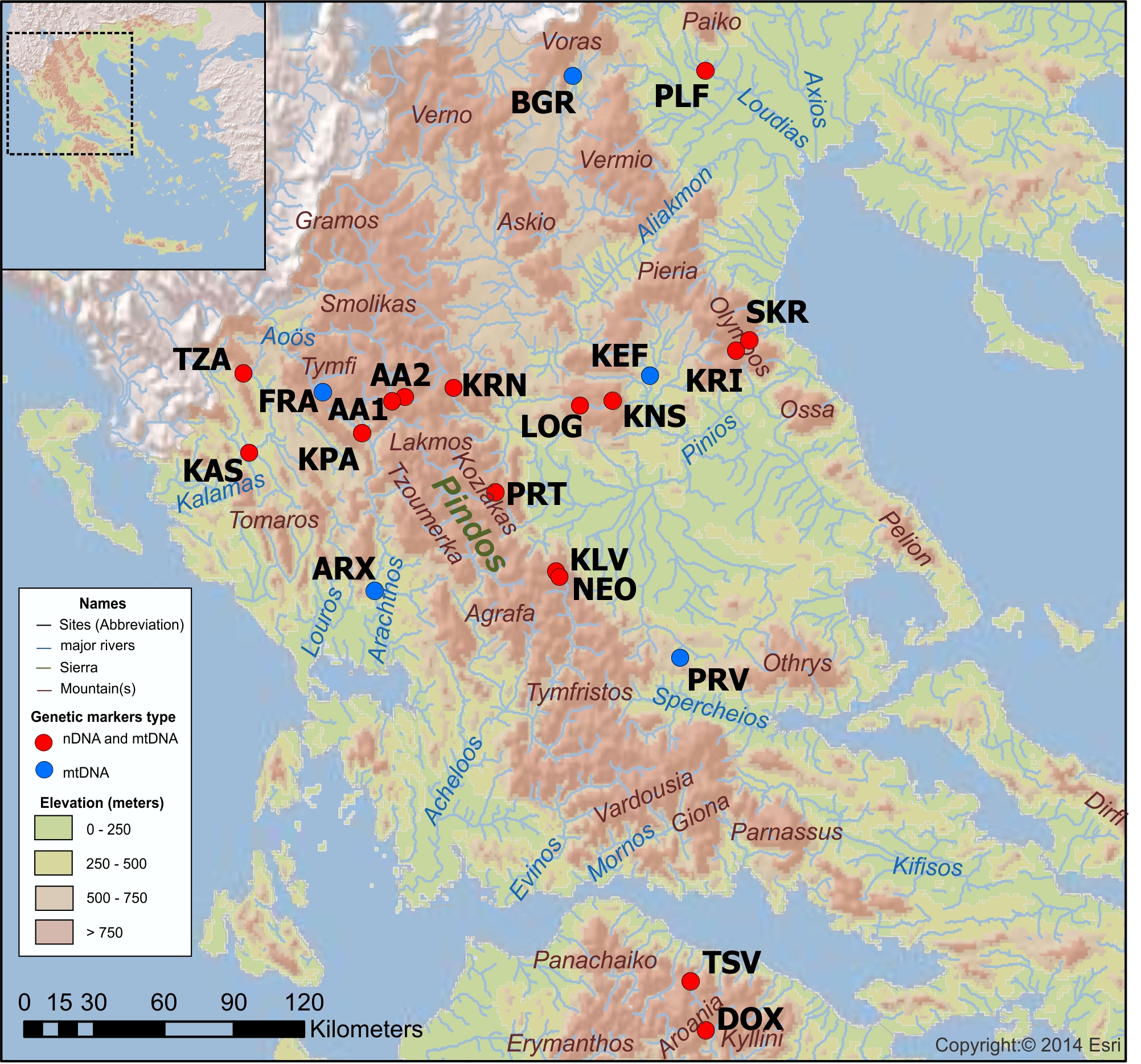

Supplement: Supplementary file 13 — Map of the sampled noble crayfish in Greece (Copyright © 2014 Esri and its licensors. All rights reserved) where abbreviation of the sampling sites, names of major rivers, mountains and sierra, type of genetic markers used (mitochondrial and/or nuclear; mtDNA and nDNA, respectively) and elevation are given. (DOC 2355 kb) [file 12862_2017_971_MOESM13_ESM.doc]
