# Supplementary material for: Microevolution of the noble crayfish (Astacus astacus) in the Southern Balkan Peninsula
Source: BMC Evol Biol. 2017 May 30;17:122. doi: 10.1186/s12862-017-0971-6 (PMC5450353; doi:10.1186/s12862-017-0971-6)
Supplement: Supplementary file 14 — Point estimates with their upper Confidence Interval (CI) for all parameters (log(N0), log(N1), log(Θ) and log(T)) and every genetic cluster for a generation time of 3.5 and 5.5. (DOC 48 kb) [file 12862_2017_971_MOESM14_ESM.doc]

# Additional file 14

Point estimates with their upper Confidence Interval (CI) for all parameters (log(N0), log(N1), log(Θ) and log(T)) and every genetic cluster for a generation time of 3.5 and 5.5.

|  |  | **log(N0)** | | **log(N1)** | | **log(Θ)** | | **log(T)** | |
| --- | --- | --- | --- | --- | --- | --- | --- | --- | --- |
| **Generation** | **Cluster** | **Point estimate** | **Upper CI** | **Point estimate** | **Upper CI** | **Point estimate** | **Upper CI** | **Point estimate** | **Upper CI** |
| 3.5 | 1 | 1.03 | 1.07 | 1 | 1.01 | 1.01 | 1.02 | 1.03 | 1.07 |
|  | 2 | 1.06 | 1.15 | 1.01 | 1.02 | 1.01 | 1.02 | 1.06 | 1.14 |
|  | 3 | 1.03 | 1.07 | 1.01 | 1.02 | 1.01 | 1.02 | 1.03 | 1.07 |
|  | 4 | 1.06 | 1.13 | 1.01 | 1.02 | 1 | 1.01 | 1.05 | 1.12 |
|  | 5 | 1.06 | 1.16 | 1 | 1.01 | 1 | 1.01 | 1.05 | 1.13 |
|  | 6 | 1.04 | 1.1 | 1 | 1.01 | 1.01 | 1.01 | 1.04 | 1.1 |
|  | 7 | 1.01 | 1.03 | 1 | 1 | 1 | 1.01 | 1.01 | 1.03 |
|  | 8 | 1.04 | 1.11 | 1 | 1.01 | 1.01 | 1.02 | 1.04 | 1.1 |
|  | 9 | 1.05 | 1.12 | 1 | 1.01 | 1 | 1.01 | 1.04 | 1.11 |
| 5.5 | 1 | 1.01 | 1.04 | 1.01 | 1.01 | 1 | 1.01 | 1.01 | 1.04 |
|  | 2 | 1.04 | 1.1 | 1.01 | 1.01 | 1 | 1.01 | 1.04 | 1.1 |
|  | 3 | 1.02 | 1.04 | 1 | 1 | 1 | 1.01 | 1.01 | 1.04 |
|  | 4 | 1 | 1.01 | 1 | 1 | 1 | 1.01 | 1 | 1 |
|  | 5 | 1.09 | 1.21 | 1 | 1.01 | 1 | 1.01 | 1.06 | 1.17 |
|  | 6 | 1.1 | 1.25 | 1 | 1.01 | 1 | 1.01 | 1.07 | 1.2 |
|  | 7 | 1.03 | 1.07 | 1 | 1.01 | 1 | 1.01 | 1.02 | 1.06 |
|  | 8 | 1.06 | 1.17 | 1.01 | 1.02 | 1 | 1.01 | 1.06 | 1.15 |
|  | 9 | 1.06 | 1.15 | 1 | 1.01 | 1 | 1.01 | 1.05 | 1.13 |
