# Supplementary material for: Microevolution of the noble crayfish (Astacus astacus) in the Southern Balkan Peninsula
Source: BMC Evol Biol. 2017 May 30;17:122. doi: 10.1186/s12862-017-0971-6 (PMC5450353; doi:10.1186/s12862-017-0971-6)
Supplement: Supplementary file 16 — Posteriors and priors densities plots for time since the population decline (T) for each genetic cluster (cluster 1 to 9) and the global data set (samples from different demes were merged into one dataset; black color). (DOC 674 kb) [file 12862_2017_971_MOESM16_ESM.doc]

# Additional file 16

Posteriors (solid lines) and priors (dotted lines) densities plots for time since the population decline (T) for each genetic cluster (cluster 1 to 9) and the global data set (samples from different demes were merged into one dataset; black color). Each genetic cluster (1 to 9) is represented by a different color (same as population structure analysis). The data set used in the analysis derived from MsVar v. 1.3 [80], assuming a generation time of 5.5. Furthermore, R package Locfit v. 1.5-9.1 [116] was used in order to produce the graphical representations.


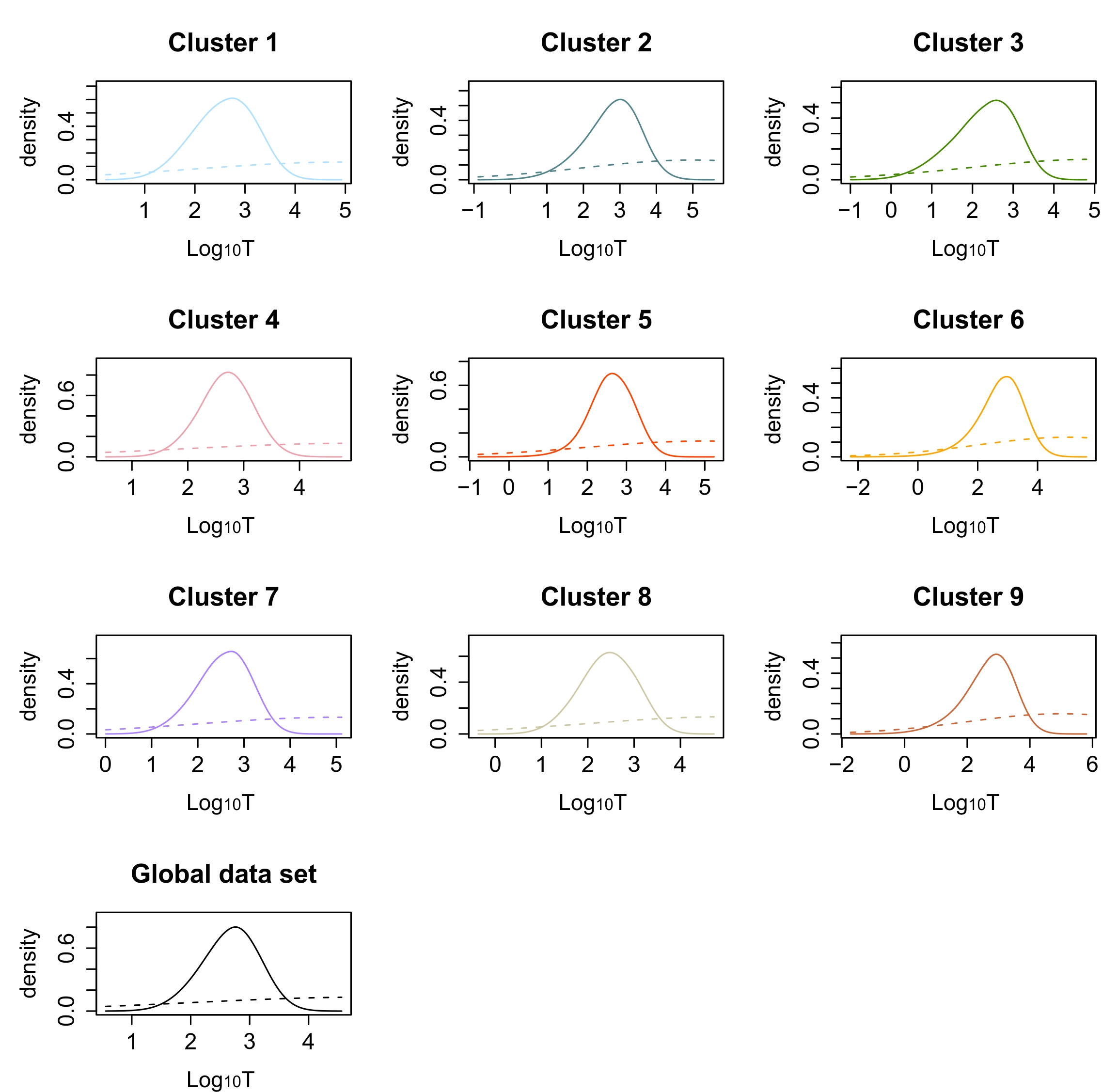


# References
