# Supplementary material for: Microevolution of the noble crayfish (Astacus astacus) in the Southern Balkan Peninsula
Source: BMC Evol Biol. 2017 May 30;17:122. doi: 10.1186/s12862-017-0971-6 (PMC5450353; doi:10.1186/s12862-017-0971-6)
Supplement: Supplementary file 17 — The relative probability of different hypotheses was assessed using approximate Bayes Factors (BF). A generation time of 5.5 for the noble crayfish was used in the analysis. Hypotheses were examined for the past 12,000 and 7000 years. (DOC 2096 kb) [file 12862_2017_971_MOESM17_ESM.doc]

# Additional file 17

The relative probability of different hypotheses (see below) was assessed using approximate Bayes Factors (BF) [80,117]. A generation time of 5.5 for the noble crayfish was used in the analysis. Different levels of BF were considered as in [85]. Hypotheses were examined for the past 12000 (**A**) and 7000 years (**B**-**D**). For Figure A, equally time intervals (50 years) grouped every 1000 years was used in order to produce 11 hypotheses (H1 to H11). For **B**, hypotheses were based on the anthropogenic influence on natural landscape, historical periods of Greece and natural phenomena: 1) H1 (0 to 200 years Before Present or BP) modern Greece with the highest anthropogenic influence (e.g. translocation of crayfish, dam constructions and river degradation), 2) H2 (200 to 1600 years BP) with a high human impact (e.g. intensive agriculture) and climatic variations (Little Ice Age and Medieval Climate Anomaly), 3) H3 (1600 to 2500 years BP) intensification of anthropogenic influence (e.g. gradual increasing complexity of hydraulic technologies), 4) H4 (2500 to 3500 years BP) climatic variation (~3200 years BP) and major Mycenaean hydraulic projects in the mainland, 5) H5 (3500 to 5000 years BP) appearance of urban hydraulic works (e.g. in Minoan and Mycenaean civilizations) and beginning of the Bronze Age (~5200 years BP), and 6) H6 (5000 to 7000 years BP) relatively low human activity (recorded Neolithic settlements and appearance of farming). BFs were computed every 50 years for a time interval between 0 and 7000 years. Additionally, BFs were also computed 1) for a time interval equally split (500 years) producing 14 hypotheses (H01 to H14; **C**); to confirm the results of the previous analysis, and 2) using historical periods of Greece, natural phenomena and smaller time intervals (20 hypothesis produced; **D**) to confirm the consistency of the hypotheses used. A graphical synopsis of the time line used to create the 20 hypothesis used in **D**, can been viewed in **E**. Each genetic cluster is represented by a different color (same as population structure analysis). Horizontal dotted line corresponds to BF value of 3. R scripts used for the Additional file 12 can be provided upon request.

**A)** Bayes Factors (BF) values for the eleven hypotheses (H1 to H11) and nine noble crayfish genetic clusters (cluster 1 to 9) for the last 12000 years.


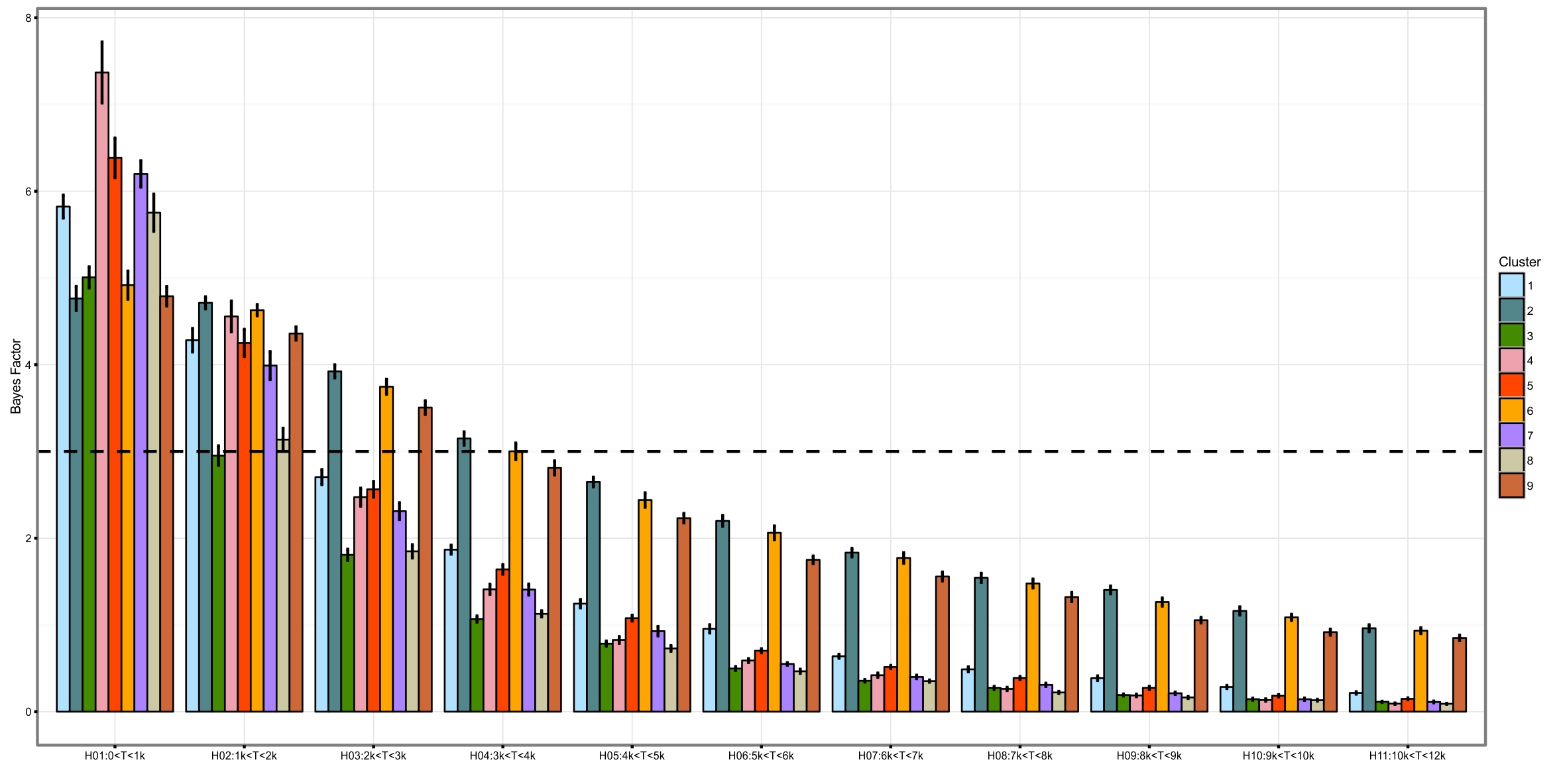


**B)** Bayes Factors (BF) values for the six hypotheses (H1 to H6) and nine noble crayfish genetic clusters (cluster 1 to 9). Hypotheses follow the division of the last 7000 years (details are provided in beginning of the document).


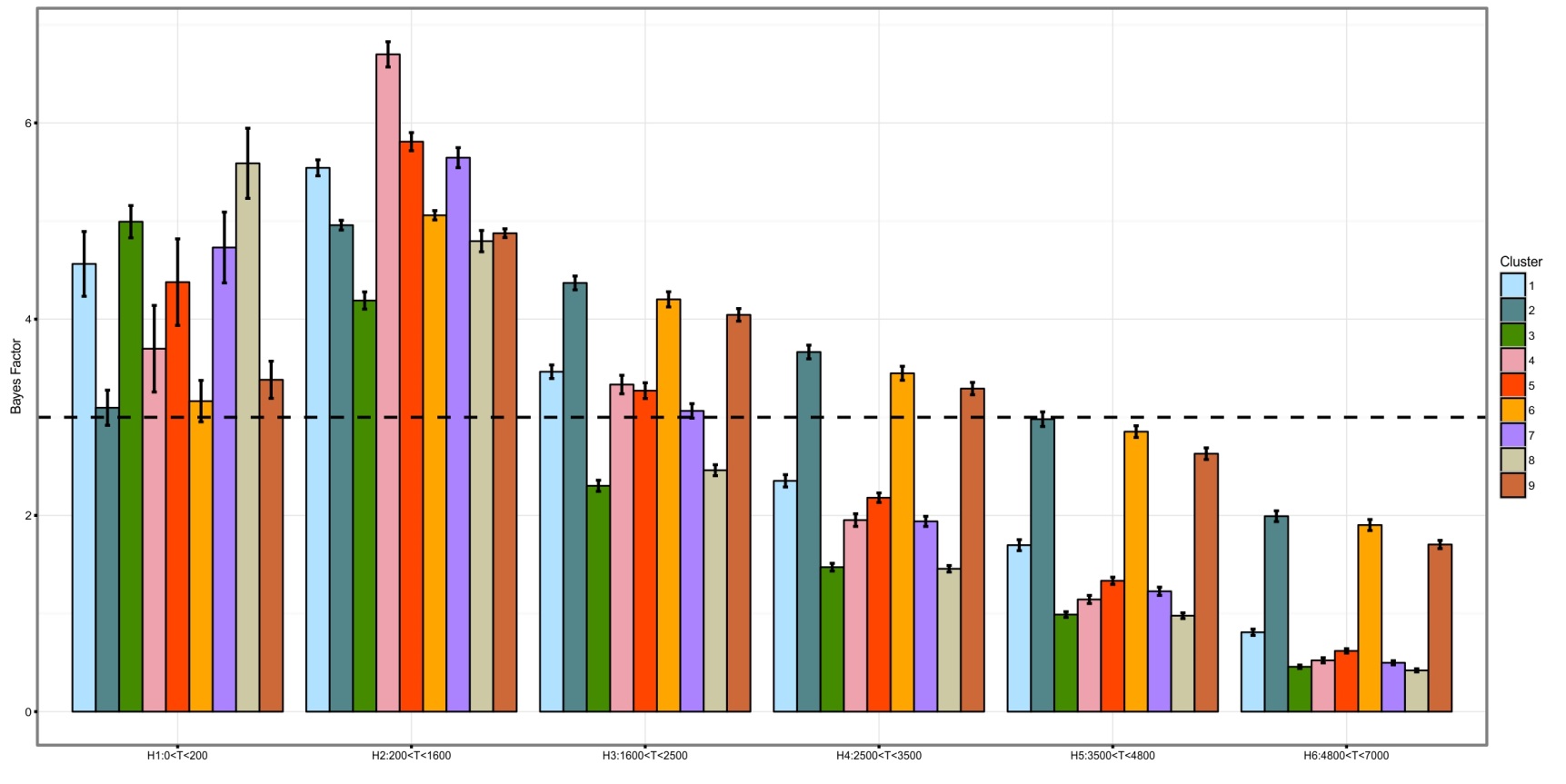


**C**) Bayes Factors (BF) values for the fourteen hypotheses (H1 to H14) and nine noble crayfish genetic clusters (cluster 1 to 9) for the last 7000 years.


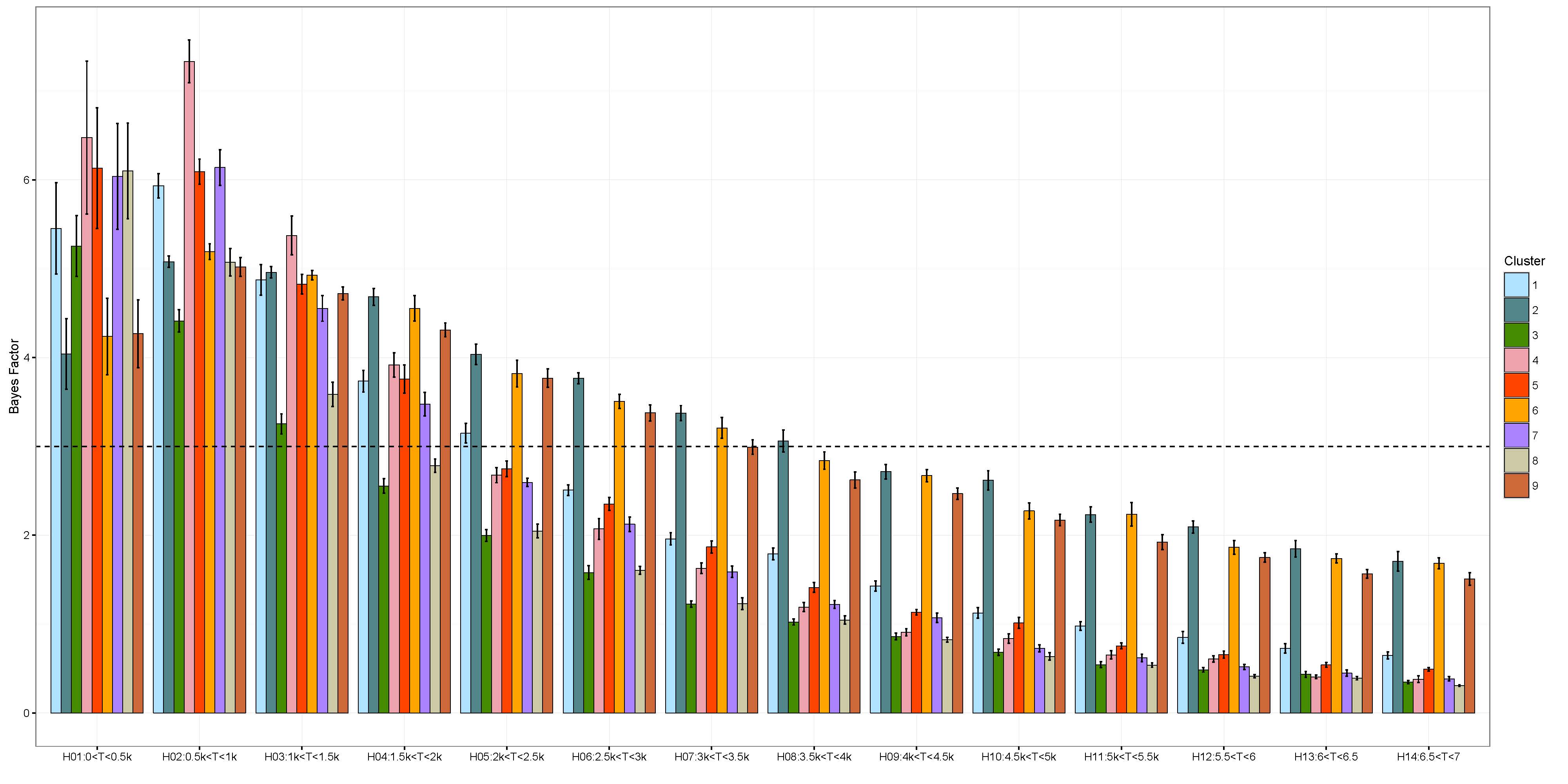


**D**) Bayes Factors (BF) values for the twenty hypotheses (H1 to H20) and nine noble crayfish genetic clusters (cluster 1 to 9) for the last 7000 years.


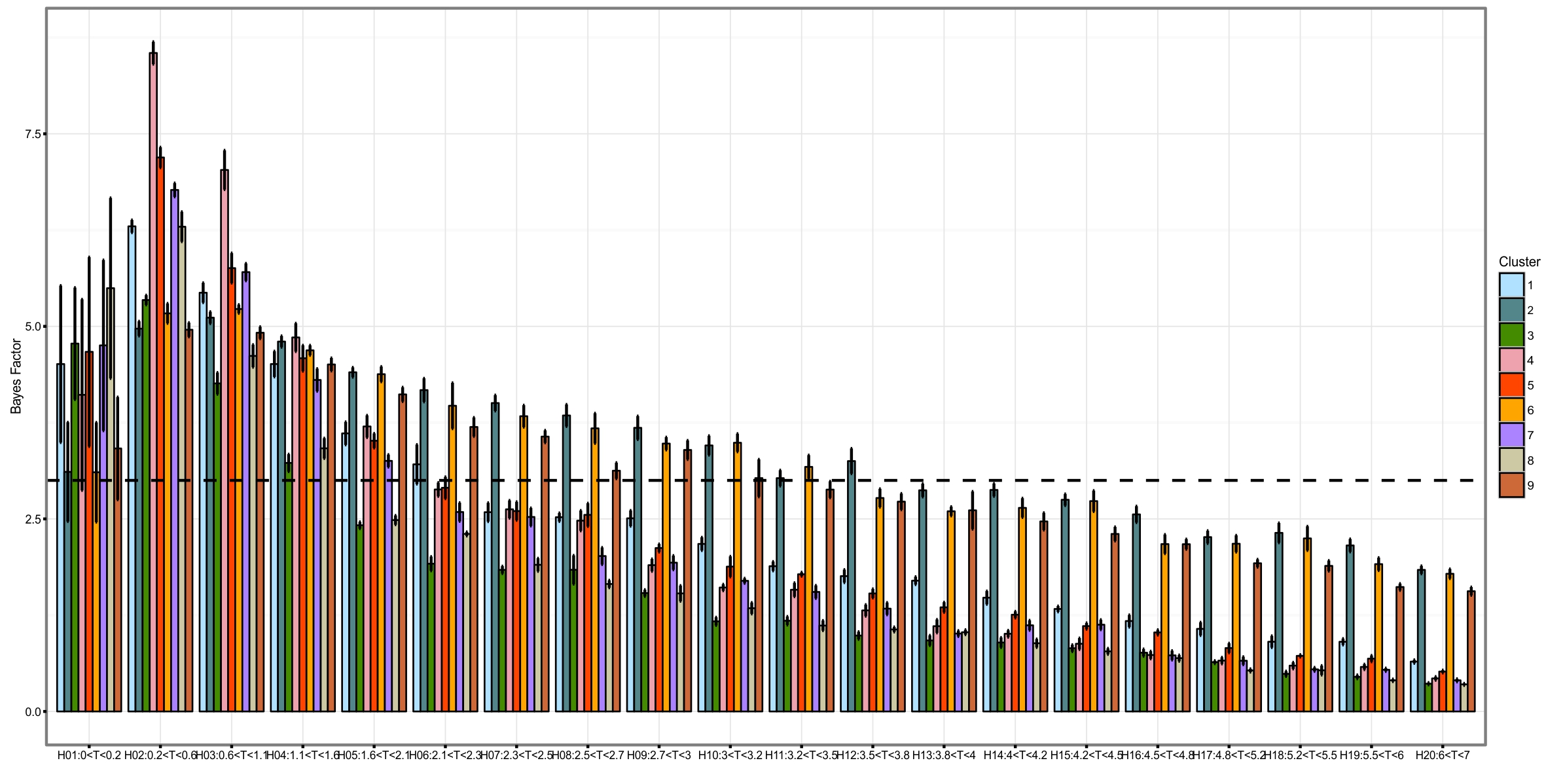


**E)** Time line of the last 7 kyr BP for twenty distinct hypotheses (H01 to H20). The time line is based on historical records (Historical Ages and Periods), anthropogenic impact on land and natural phenomena of Greece.


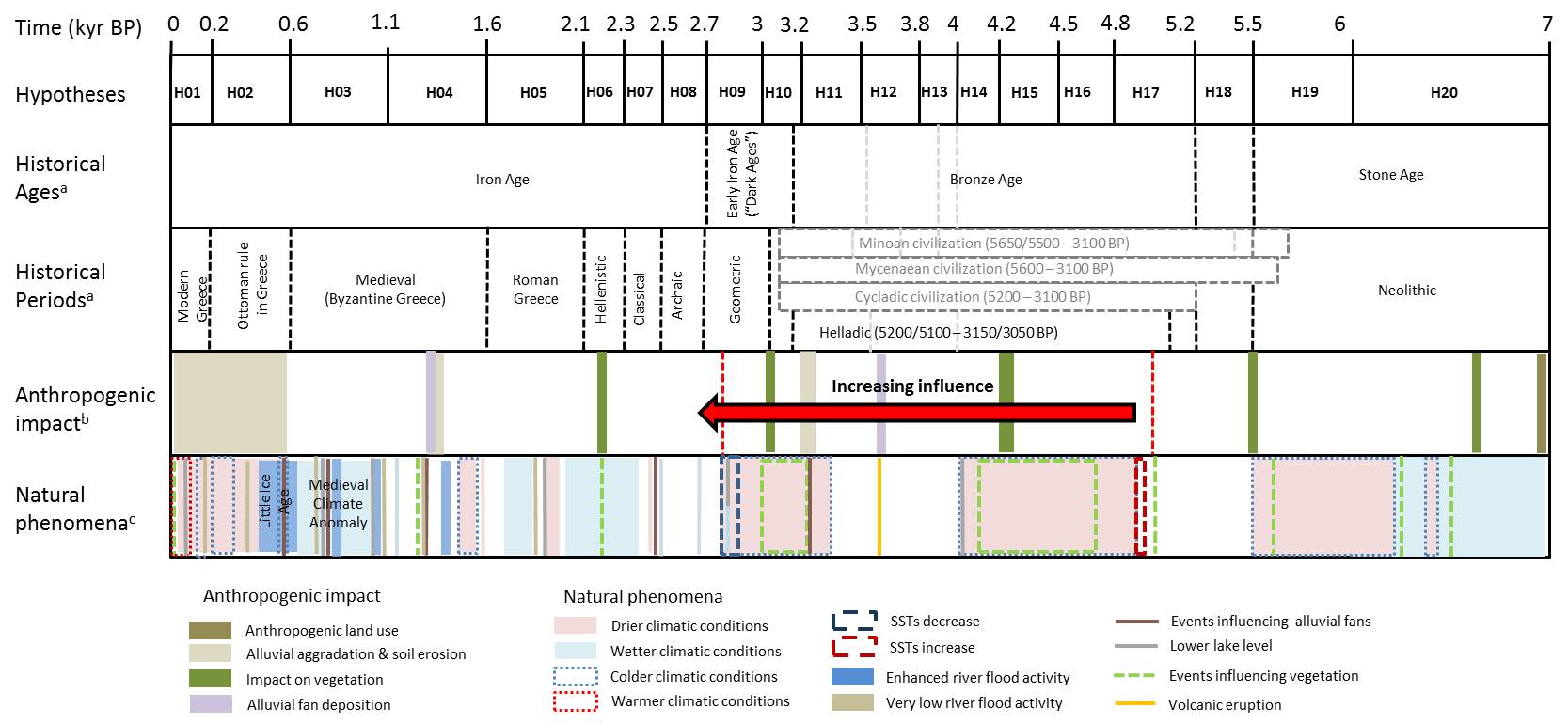


^a^ based on [110,111,118–120] and references therein.

^b^ based on [109,121–127].

^c^ based on [105,106,109,121,122,124,125,128–135].

# References

80. Storz JF, Beaumont MA. Testing for genetic evidence of population expansion and contraction: an empirical analysis of microsatellite DNA variation using a hierarchical Bayesian model. Evolution. 2002;56:154–66.

105. Büntgen U, Myglan VS, Ljungqvist FC, McCormick M, Di Cosmo N, Sigl M, et al. Cooling and societal change during the Late Antique Little Ice Age from 536 to around 660 AD. Nat. Geosci. 2016;9:231–6.

106. Luterbacher J, García-Herrera R, Akcer-On S, Allan R, Alvarez-Castro MC, Benito G, et al. A review of 2000 years of paleoclimatic evidence in the mediterranean. In: Lionello P, editor. Clim. Mediterr. Reg. - From past to Futur. Amsterdam: Elsevier; 2012. p. 87–185.

109. Lespez L. Geomorphic responses to long-term land use changes in Eastern Macedonia (Greece). CATENA. 2003;51:181–208.

110. Viollet P-L. Water engineering in ancient civilizations: 5,000 years of history. Boca Raton: CRC Press; 2007.

111. Bintliff J. The complete archaeology of Greece: from hunter-gatherers to the 20th Century A.D. 1st ed. Chichester: Wiley-Blackwell; 2012.

117. Quéméré E, Amelot X, Pierson J, Crouau-Roy B, Chikhi L. Genetic data suggest a natural prehuman origin of open habitats in northern Madagascar and question the deforestation narrative in this region. Proc. Natl. Acad. Sci. U. S. A. 2012;109:13028–33.

118. Angelakis AN, Mays LW, Koutsoyiannis D, Mamassis N. Evolution of water supply through the millennia. London: IWA Publishing; 2012.

118. Angelakis AN, Mays LW, Koutsoyiannis D, Mamassis N. Evolution of water supply through the millennia. London: IWA Publishing; 2012.

119. Harris W V. Defining and detecting Mediterranean deforestation, 800 BCE to 700 CE. In: Harris W V, editor. The Ancient Mediterranean environment between science and history. between Sci. Hist. Leiden, Boston: Brill; 2013. p. 173–325.

120. Pomeroy SB, Donlan W, Burstein SM, Tolbert Roberts J. A brief history of ancient Greece: politics, society, and culture. New York and Oxford: Oxford University Press; 2004.

121. Willis KJ. The late Quaternary vegetational history of northwest Greece. II. Rezina marsh. New Phytol. 1992;121:119–38.

122. Willis KJ. The late Quaternary vegetational history of northwest Greece. III. A comparative study of two contrasting sites. New Phytol. 1992;121:139–55.

123. Jahns S. On the Holocene vegetation history of the Argive Plain (Peloponnese, southern Greece). Veg. Hist. Archaeobot. 1993;2:187–203.

124. Pope RJJ, Wilkinson KN. Reconciling the roles of climate and tectonics in Late Quaternary fan development on the Spartan piedmont, Greece. Geol. Soc. London. 2005;251:133–52.

125. Kotthoff U, Müller UC, Pross J, Schmiedl G, Lawson IT, van de Schootbrugge B, et al. Lateglacial and Holocene vegetation dynamics in the Aegean region: an integrated view based on pollen data from marine and terrestrial archives. The Holocene. 2008;18:1019–32.

126. Gerasimidis A, Panajiotidis S, Fotiadis G, Korakis G. Review of the Late Quaternary vegetation history of Epirus (NW Greece). Phytol. Balc. 2009;15:29–37.

127. Psomiadis D, Ghilardi M, Demory F, Delanghe-Sabatier D, Bloemendal J, Yiu C. Late Pleistocene to Mid Holocene landscape reconstruction in the western part of the Thessaloniki Plain, Greece: evidence for environmental changes, and implications for human occupation. Z. Geomorphol. 2014;58:67–87.

128. Repapis CC, Schuurmans CJE, Zerefos CS, Ziomas J. A note on the frequency of occurrence of severe winters as evidenced in monastery and historical records from Greece during the period 1200-1900 A.D. Theor. Appl. Climatol. 1989;39:213–7.

129. Emeis KC, Struck U, Schulz HM, Rosenberg R, Bernasconi S, Erlenkeuser H, et al. Temperature and salinity variations of Mediterranean Sea surface waters over the last 16,000 years from records of planktonic stable oxygen isotopes and alkenone unsaturation ratios. Palaeogeogr. Palaeoclimatol. Palaeoecol. 2000;158:259–80.

130. Rohling EJ, Mayewski PA, Abu-Zied RH, Casford JSL, Hayes A. Holocene atmosphere-ocean interactions: records from Greenland and the Aegean sea. Clim. Dyn. 2002;18:587–93.

131. Dormoy I, Peyron O, Combourieu-Neboutb N, Goring S, Kotthoff U, Magny M, et al. Terrestrial climate variability and seasonality changes in the Mediterranean region between 15000 and 4000 years BP deduced from marine pollen records. Clim. Past Discuss. 2009;5:735–70.

132. Drake BL. The influence of climatic change on the Late Bronze Age Collapse and the Greek Dark Ages. J. Archaeol. Sci. 2012;39:1862–70.

133. Cvetkoska A, Levkov Z, Reed JM, Wagner B. Late glacial to holocene climate change and human impact in the Mediterranean: The last ca. 17ka diatom record of lake Prespa (Macedonia/Albania/Greece). Palaeogeogr. Palaeoclimatol. Palaeoecol. 2014;406:22–32.

134. Finné M. Climate in the eastern Mediterranean during the Holocene and beyond – A Peloponnesian perspective. Stockholm University; 2014.

135. Xoplaki E, Fleitmann D, Luterbacher J, Wagner S, Haldon JF, Zorita E, et al. The Medieval Climate Anomaly and Byzantium: A review of the evidence on climatic fluctuations, economic performance and societal change. Quaternary Sci. Rev. 2016;136:229–52.
